# The *Tetracentron* genome provides insight into the early evolution of eudicots and the formation of vessel elements

**DOI:** 10.1186/s13059-020-02198-7

**Published:** 2020-12-02

**Authors:** Ping-Li Liu, Xi Zhang, Jian-Feng Mao, Yan-Ming Hong, Ren-Gang Zhang, Yilan E, Shuai Nie, Kaihua Jia, Chen-Kun Jiang, Jian He, Weiwei Shen, Qizouhong He, Wenqing Zheng, Samar Abbas, Pawan Kumar Jewaria, Xuechan Tian, Chang-jun Liu, Xiaomei Jiang, Yafang Yin, Bo Liu, Li Wang, Biao Jin, Yongpeng Ma, Zongbo Qiu, František Baluška, Jozef Šamaj, Xinqiang He, Shihui Niu, Jianbo Xie, Lei Xie, Huimin Xu, Hongzhi Kong, Song Ge, Richard A. Dixon, Yuannian Jiao, Jinxing Lin

**Affiliations:** 1 Beijing Advanced Innovation Center for Tree Breeding by Molecular Design, Beijing Forestry University, Beijing, 100083 China; 2 College of Biological Sciences & Biotechnology, Beijing Forestry University, Beijing, 100083 China; 3 Beijing Ori-Gene Science and Technology Co., Ltd, Beijing, 102206 China; 4 College of Life Sciences, Peking University, Beijing, 100871 China; 5 School of Ecology and Nature conservation, Beijing Forestry University, Beijing, 100083 China; 6 Biology Department, Brookhaven National Laboratory, Upton, NY 11973 USA; 7 Wood Collections (WOODPEDIA), Chinese Academy of Forestry, Beijing, 100091 China; 8 Department of Wood Anatomy and Utilization, Research Institute of Wood Industry, Chinese Academy of Forestry, Beijing, 100091 China; 9 College of Horticulture and Plant Protection, Yangzhou University, Yangzhou, 225009 China; 10 Yunnan Key Laboratory for Integrative Conservation of Plant Species with Extremely Small Populations, Kunming Institute of Botany, Chinese Academy of Sciences, Kunming, 650201 China; 11 College of Life Science, Henan Normal University, Xinxiang, 453007 China; 12 Institute of Cellular and Molecular Botany (IZMB), University of Bonn, Kirschallee 1, 53115 Bonn, Germany; 13 Centre of the Region Haná for Biotechnological and Agricultural Research, Faculty of Science, Palacký University, 78301 Olomouc, Czech Republic; 14 State Key Laboratory of Protein and Plant Gene Research, College of Life Sciences, Peking University, Beijing, 100871 China; 15 College of Biological Sciences, China Agricultural University, Beijing, 100193 China; 16 Institute of Botany, Chinese Academy of Sciences, Beijing, 100093 China; 17 BioDiscovery Institute and Department of Biological Sciences, University of North Texas, Denton, TX 76203 USA

**Keywords:** *Tetracentron sinense*, Vessel, Phylogenomic, Whole genome duplication, VND7, Resequencing, Genetic diversity

## Abstract

**Background:**

*Tetracentron sinense* is an endemic and endangered deciduous tree. It belongs to the Trochodendrales, one of four early diverging lineages of eudicots known for having vesselless secondary wood. Sequencing and resequencing of the *T. sinense* genome will help us understand eudicot evolution, the genetic basis of tracheary element development, and the genetic diversity of this relict species.

**Results:**

Here, we report a chromosome-scale assembly of the *T. sinense* genome. We assemble the 1.07 Gb genome sequence into 24 chromosomes and annotate 32,690 protein-coding genes. Phylogenomic analyses verify that the Trochodendrales and core eudicots are sister lineages and showed that two whole-genome duplications occurred in the Trochodendrales approximately 82 and 59 million years ago. Synteny analyses suggest that the γ event, resulting in paleohexaploidy, may have only happened in core eudicots. Interestingly, we find that vessel elements are present in *T. sinense*, which has two orthologs of *AtVND7*, the master regulator of vessel formation. *T. sinense* also has several key genes regulated by or regulating *TsVND7.2* and their regulatory relationship resembles that in *Arabidopsis thaliana.* Resequencing and population genomics reveals high levels of genetic diversity of *T. sinense* and identifies four refugia in China.

**Conclusions:**

The *T. sinense* genome provides a unique reference for inferring the early evolution of eudicots and the mechanisms underlying vessel element formation. Population genomics analysis of *T. sinense* reveals its genetic diversity and geographic structure with implications for conservation.

**Supplementary information:**

The online version contains supplementary material available at 10.1186/s13059-020-02198-7.

## Background

Eudicots are the largest and most diverse group of angiosperms, containing 75% of all angiosperm species [1]. Recent phylogenetic studies identified several early diverging eudicot branches and a strongly supported clade called the core eudicots [2, 3] comprising the majority of eudicot species. The rise and eventual dominance of the core eudicots may have benefited from the well-known γ hexaploidization event in their most recent common ancestor [4]. However, the timing and nature of the γ event are still controversial, and a recent study proposed a two-step model for the γ event, with the first whole-genome duplication occurring in the common ancestor of all eudicots [5]. Sequencing more early diverging eudicots can help us understand the nature of the γ event, as well as other genomic changes that occurred during the early diversification of eudicots.

Within eudicots, the four extant eudicot lineages that diverged the earliest are the Ranunculales, Proteales, Trochodendrales, and Buxales [6, 7]. The order Trochodendrales consists of one family (Trochodendraceae) and two species (*Tetracentron sinense* and *Trochodendron aralioides*). Although there has been controversy about the phylogenetic position of the Trochodendrales [8, 9], most phylogenetic analyses using organellar and nuclear genes strongly support the placement of Trochodendrales as early diverging eudicots [6, 7, 10, 11]. Whole-genome sequences from several Ranunculales and Proteales are available [5, 12, 13], genomes from Trochodendrales such as *T. sinense* will facilitate the comparative genomic analyses and shed deeper sight into the early evolution of eudicots.

Xylem, the water-conducting tissue in vascular plants, is composed of different cell types. The tracheids and vessel elements are conducting cells of the xylem [14]. Vessel elements are one of the most important features of angiosperms and differentiate them from other vascular plants such as gymnosperms (except gnetophytes [15]), which lack vessel elements. As they differentiate, vessel elements produce secondary cell walls and eventually undergo programmed cell death (PCD), forming a hollow tube that conducts water from the roots to the apical parts of the plant [14]. For almost 150 years, *T. sinense* was considered to produce secondary xylem that has tracheids but lacks vessel elements [16–18]. However, some studies suggest that vessel elements are present in the secondary xylem of *T. sinense* [19, 20]. Therefore, whether the secondary xylem of *T. sinense* contains vessel elements remains an outstanding question that can be addressed by morphological studies, and examination of the genetic network that specifies vessel elements.

VASCULAR RELATED NAC-DOMAIN PROTEIN 7 (VND7) functions as a master regulator of vessel element formation [21]. VND7 directly regulates many genes such as the transcription factor genes *MYB46* and *MYB83*, the cellulose synthase (CES) genes *CESA4* and *CESA8*, and the xylem-specific papain-like cysteine peptidase (XCP) genes *XCP1* and *XCP2* [22–26]. These genes mediate secondary cell wall formation and PCD, the two essential processes of vessel element differentiation. *VND7* is positively regulated by several factors, including the LOB DOMAIN-CONTAINING PROTEINs (LBDs) LBD18 and LBD30, and VND1–VND5 [14, 27, 28]. Whether an ortholog of *VND7* is present in *T. sinense* and whether the regulatory network of *T. sinense VND7* parallels the *Arabidopsis thaliana* regulatory network remain unknown.

*T. sinense* (2n = 48 [29]) is a tertiary relict species that is mainly found in eastern Asia, including eastern Nepal and southwestern and central China [30–32]. The climate change during the Pleistocene ice age and human disturbances in recent years resulted in a sharp decline in *T. sinense* population size [32]. *T. sinense* has been documented as an endangered species in China and is currently listed in Appendix III of the Convention of International Trade of Endangered Species [31]. Although the evolutionary history of *T. sinense* has been investigated by examination of a few plastid regions and inter-simple sequence repeat analysis [31, 32], controversies remain, including the identification of potential refugia. In addition, little is known about the genome-scale genetic diversity and the population dynamics of this species in response to climate change, from the Pleistocene to the present. Genome-wide resequencing of *T. sinense* accessions from different populations would help us assess the levels and patterns of genetic variation and the evolutionary history of this endangered species, providing a practical strategy for its conservation.

Here we report the sequencing, assembly, and annotation of the *T. sinense* genome and its resequencing in key accessions. Our major objectives were (1) to explore the genomic changes that occurred during the early evolution of eudicots and core eudicots, (2) to investigate whether *T. sinense* has vessel elements in its secondary xylem and whether it has VND7 (and if so, whether the VND7 regulatory network is as in *A. thaliana*), and (3) to investigate the genetic diversity and evolutionary history of *T. sinense*. Our results reveal the role of polyploidization in the early evolution of eudicots and the nature of the known γ event, as well as exploring other genomic changes that occurred during eudicot diversification. Our genome-wide resequencing provided further insight into the evolutionary history of *T. sinense*. Moreover, light microscopy and the scanning electron microscopy (SEM) observations of slices of *T. sinense* wood and analyses of X-ray computed microtomography (microCT) support the idea that vessel elements are present in the secondary xylem of *T. sinense*. We also investigated the genetic basis of the development of vessel elements by identifying *T. sinense* orthologs of *VND7* and exploring their regulation. Therefore, our results provide genomic insight on the evolution of this intriguing species and on the evolution of xylem vessels.

## Results

### SMRT sequence and assembly of the *T. sinense* genome

To analyze the genome of *T. sinense*, we first generated 82 G Illumina paired-end reads (150 bp). The genome size of the sequenced individual was estimated to be ~ 1.12 Gb, and its heterozygosity rates were estimated to be 0.22%, based on *k*-mer analysis (*k*-mer frequency distributions) (Additional file 1: Fig. S1). We then used a combination of single-molecule real-time (SMRT) sequencing technology from Pacific Biosciences (PacBio) and high-throughput chromosome conformation capture (Hi-C) to produce the final sequenced and assembled genome of *T. sinense*. In total, we generated 14.8 M PacBio single-molecule long reads (average read length 8 kb, longest read 84 kb), for 118 Gb total sequence, giving ~ 100× coverage of the assembled genome. De novo assembly yielded 3389 contigs, with a contig N50 length of 1.99 Mb, which was further improved to 2.8 Mb with Gapcloser software [33]. The total assembly size was 1.17 Gb, which is consistent with the genome size estimated by *k*-mer analysis (Additional file 1: Fig. S1).

To further refine the *T. sinense* assembly, Hi-C libraries were constructed and sequenced. The Hi-C read pairs were mapped onto the draft assembly (Additional file 1: Fig. S2), yielding an assembly with a scaffold N50 of 45 Mb. The final reference assembly comprised 24 chromosome-scale pseudomolecules hereafter referred to as chromosomes (Additional file 1: Fig. S2b), with maximum and minimum lengths of 65 Mb and 33 Mb, respectively. The total length of the chromosomes accounted for 92.24% (1.07 Gb) of the assembled genome size of 1.17 Gb. The assembled chromosome number is equal to previous estimates of the haploid chromosome number of *T. sinense* (2n = 48). BUSCO [34] assessment showed that 93.4% complete genes were captured.

### Repeat and gene annotation

We identified 787 Mb of repetitive sequences in the genome of *T. sinense*, accounting for 67.8% of the genomic assembly. Transposable elements were the predominant component (65.9%), with the long terminal repeat (LTR) family being the largest part (32.2%) of these transposons. Within the LTR family, the *Gypsy* subfamily was the most abundant, making up 20.5% of the genome, followed by the *Copia* subfamily (11%) (Additional file 1: Table S1).

Based on the remaining repeat-masked *T. sinense* genome, we annotated 32,690 protein-coding gene models by combining ab initio gene predictions and evidence-based gene annotations using the MAKER pipeline. These gene models had an average transcript length of 1492 bp and an average coding-sequence length of 1304 bp. The gene models contained an average of 5.4 exons per gene with average exon and intron lengths of 153 bp and 283 bp, respectively. In addition, we identified 166 rRNAs, 811 tRNAs, and 1062 non-coding RNAs (ncRNAs). A BUSCO assessment based on conserved plant gene models identified 93.8% complete gene models.

### Phylogenomic and gene family evolution analyses

**Fig. 1:**
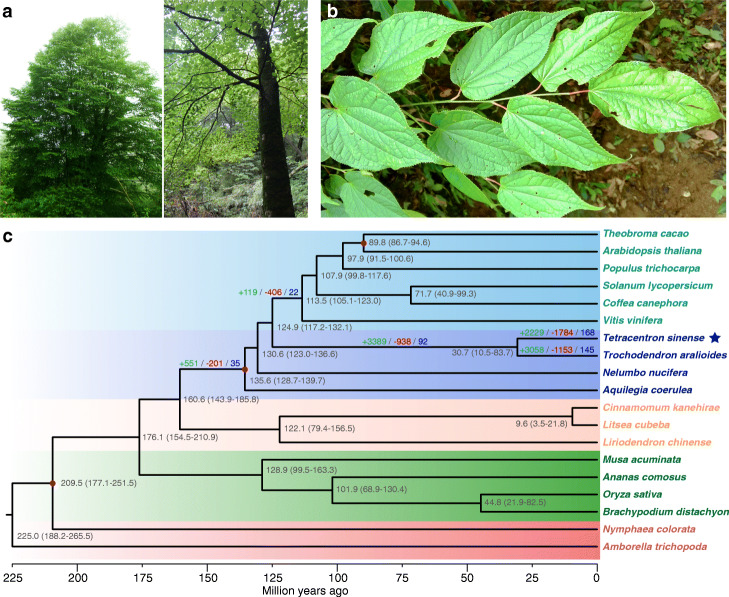
Tree and phylogenetic tree. **a**, **b** A typical mature tree of *Tetracentron sinense.* **c** Phylogenetic tree of nineteen species including divergence times based on 214 single-copy nuclear genes. Divergence times are indicated by the numbers around each node with 95% highest posterior density. The red dot represents a calibration point. All nodes have 100% bootstrap support in the Maximum Likelihood analysis. Light blue indicates core eudicots, dark blue indicates early diverging eudicots, orange indicates magnoliids, green indicates monocots, and light red indicates basal angiosperms. Green, red, and blue numbers above branches indicate the number of gene families that have expanded, contracted, and rapidly evolved (*p* value ≤ 0.01), respectively

To ascertain the phylogenetic position of *T. sinense* and the Trochodendrales, we performed phylogenomic analysis using 214 single-copy gene families (orthogroups) from 19 sequenced angiosperm genomes identified by OrthoFinder v2.3.1 [35]. These orthologous groups with single-copy genes were concatenated to construct a maximum likelihood (ML) phylogenetic tree using IQ-TREE. As shown in the ML tree (Fig. 1), the major lineages of angiosperms (monocots, magnoliids, and eudicots) were all recovered as monophyletic groups with 100% support. In eudicots, the core eudicot species formed a strongly supported monophyletic clade (100% bootstrap support). The Ranunculales, Proteales, and Trochodendrales formed successive sister clades to the core eudicots with 100% support, consistent with previous analyses [6, 7, 36].

We further estimated the divergence times of eudicot lineages using the MCMCTree [37] and BEAST 2 [38] programs; these two methods yielded consistent divergence times with only small differences (Fig. 1; Additional file 1: Fig. S3). According to the MCMCTree analysis (Fig. 1), crown-group eudicots arose 136 million years ago (MYA; 129–140 MYA, 95% highest posterior density). Apart from the Ranunculales, the earliest divergences within the eudicots based on our analyses were the split of the Proteales and Trochodendrales from the rest of the eudicots at ~ 131 (123–137) MYA and ~ 125 (117–132) MYA, respectively. In our analysis, clades corresponding to the core eudicots originated at ~ 113 (105–123) MYA. Within the Trochodendrales, the divergence of its two species was estimated to have occurred at 31 (11–84) MYA.

We also investigated gene family evolution during the early diversification of eudicots. According to a CAFE analysis [39], five times as many gene families expanded in the lineage leading to all eudicots (551), as compared to core eudicots (110). However, twice as many gene families contracted in the lineage leading to core eudicots compared with the lineage leading to all eudicots (Fig. 1; Additional file 1: Fig. S4), consistent with a number of characteristics shared by eudicots. We found that in the lineages leading to eudicots, core eudicots, and the most recent ancestor of Trochodendrales, some defense gene families evolved rapidly. In the lineage leading to core eudicots, the TM3 MAD-box gene family that includes the important flowering time gene *SUPPRESSOR OF OVEREXPRESSION OF CONSTANS1* has expanded (Additional file 1: Fig. S5a).

In the lineage leading to the common ancestor of the Trochodendrales, there was much more gene family expansion (3524) compared to gene family contraction (938). To the genome of *T. sinense*, 2229 and 1784 gene families had undergone expansion and contraction, respectively. Among these families, 168 evolved rapidly. In contrast, more gene families expanded (3058) and fewer gene families (1153) contracted in *T. trochodendron.* The rapidly evolving gene families of *T. sinense* include many genes enriched in functions related to defense, toxin catabolic process, response to salicylic acid, and oxidative stress (Table S2). These genes mainly belong to gene families, such as the defense-related *NBS-LRR* (nucleotide-binding site leucine-rich repeat), lectin *RLK* (receptor-like kinase), and *GST* (glutathione transferase) gene families (Additional file 1: Fig. S5b). The gene families involved in terpenoid biosynthesis also evolved rapidly (Additional file 1: Table S2). Furthermore, the *ALG24* MAD-box genes expanded rapidly in *T. sinense* (Additional file 1: Fig. S5c). In agreement with the enrichment of GO terms, the KEGG enrichment suggested that genes involved in plant–pathogen interaction, glutathione metabolism, and terpenoid biosynthesis evolved rapidly in the *T. sinense* genome (Additional file 1: Fig. S6).

### Whole-genome duplications

**Fig. 2:**
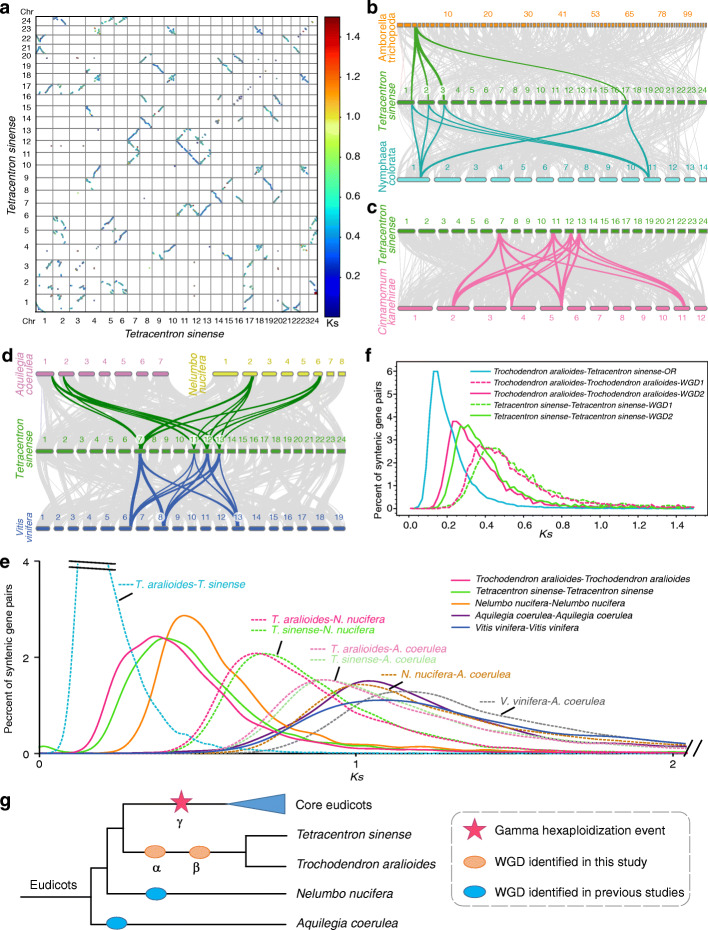
Two WGDs occurred in the Trochodendrales. **a** Dot plot showing the DNA sequence alignment of 24 chromosomes in *T. sinense*. Dots representing the position of paralogous gene-pairs were colored according to the *Ks* color scale. **b** Syntenic pattern among genomic regions in *A. trichopoda*, *T. sinense*, and *N. colorata*. Each *Amborella* region aligns with up to with four regions in *Tetracentron* that resulted from two WGDs in the early Trochodendrales. Four homologous *Tetracentron* regions derived from two WGDs align with two distinct *N. colorata* regions. Examples are highlighted in color in plots **b**, **c**, and **d**. **c** Syntenic pattern among genomic regions in *T. sinense* and *C. kanehirae*. **d** Syntenic pattern among genomic regions in *A. coerulea*, *N. nucifera*, *T. sinense*, and *V. vinifera*. **e** Synonymous substitution rate (*Ks*) distributions of syntenic blocks for the *T. sinense* paralogs and orthologs with other species. **f** *Ks* distributions of syntenic blocks for paralogs attributed to the α and β WGDs in the Trochodendrales, and orthologs between the two species of Trochodendrales without out-paralogs. **g** Summary of polyploidy events in the history of eudicots

Whole-genome duplications (WGDs, polyploidization) have played a major role in the evolutionary history of the angiosperms. Intragenomic syntenic analysis of *T. sinense* showed strong collinearity between large segments of chromosomes, such as 2, 5, 6, and 24; 4, 9, 15, and 19; 4, 10, 18, and 23; 7, 11, 12, and 13; and 8, 14, 16, and 20 (Fig. 2a). The widespread occurrence of one-versus-three syntenic blocks suggested that two WGD events (named α and β) have occurred in the genome of *T. sinense*.

*T. aralioides* is a sister of *T. sinense*. To investigate whether *T. aralioides* also experienced WGDs, we performed an intragenomic analysis of this species. Indeed, two WGDs also occurred in *T. aralioides* since intragenome comparisons showed a 1:3 syntenic depth ratio (Additional file 1: Fig. S7a)*.* To explore if the WGDs are common or lineage-specific, we performed intergenomic syntenic analyses between *T. sinense* and *T. aralioides.* The results showed a 1:1 syntenic relationship between *T. sinense* and *T. aralioides* (Additional file 1: Fig. S7b), suggesting that the two WGDs are common to the Trochodendrales.

Furthermore, we assessed the synteny between *T. sinense* or *T. aralioides* and *Amborella trichopoda*, which is the only living representative of the sister lineage to all other extant flowering plants and did not experience lineage-specific WGD events [40]. We identified a 1:4 syntenic depth ratio in the *A. trichopoda*–*T. sinense* (Fig. 2b; Additional file 1: Fig. S8a) and *A. trichopoda*–*T. aralioides* (Additional file 1: Fig. S8b) comparisons. We also performed intergenomic comparisons between *T. sinense*/*T. aralioides* and several species including the basal angiosperm *Nymphaea colorata*, the magnoliid *Cinnamomum kanehirae*, two early diverging eudicots (*Aquilegia coerulea* and *Nelumbo nucifera*), and the core eudicot *Vitis vinifera.* Genomic comparisons of *T. sinense*/*T. aralioides* and *N. colorata* showed a 4:2 syntenic pattern, and comparisons of *T. sinense*/*T. aralioides* and *C. kanehirae* showed a 4:4 syntenic pattern (Fig. 2b, c; Additional file 1: Fig. S8c-f). Given the known lineage-specific WGD in *N. colorata* and two lineage-specific WGDs in *C. kanehirae* [41, 42], our results suggested that the two WGDs in Trochodendrales occurred after the divergence of eudicots and magnoliids.

In addition, the synteny relationship between *N. nucifera* and *N. colorata* and between *N. nucifera* and *C. kanehirae* is 2:2 and 2:4, respectively (Additional file 1: Fig. S8g, h), indicating one WGD in *N. nucifera* after the divergence from *N. colorata* and *C. kanehirae.* Several earlier studies showed that the WGD in *N. nucifera* is lineage-specific rather than eudicot-wide, rejecting the possibility that any WGD occurred in the common ancestor of all extant eudicots [13, 43, 44]. The collinear relationships between *T. sinense*/*T. aralioides* and *A. coerulea*, *N. nucifera*, and *V. vinifera* are 4: 2, 4:2, and 4:3 (Fig. 2d; Additional file 1: Fig. S9), respectively, consistent with independent WGDs in the respective lineages of the Trochodendrales, *A. coerulea*, *N. nucifera*, and *V. vinifera.*

Distributions of synonymous substitutions per synonymous site (*Ks*) were further analyzed to confirm the WGDs in Trochodendrales and other early diverging lineages of eudicots. The *Ks* distributions of *T. sinense* paralogs and *T. aralioides* paralogs in syntenic regions showed two overlapping peaks supporting two WGDs in a common ancestor of *T. sinense* and *T. aralioid*es (Fig. 2e). As shown in Fig. 2e, the peaks of *Ks* distribution of *T. sinense/T. aralioides* paralogs (retained from two WGD events) are much smaller than those of orthologs comparing *T. sinense/T. aralioides* to *A. coerulea* and to *N. nucifera*, strongly suggesting the two WGDs in *T. sinense/T. aralioides* occurred after their divergence from *A. coerulea* and *N. nucifera*. In addition, the *N. nucifera* paralogs displayed lower peak *Ks* value than orthologs comparing *N. nucifera* to *T. sinense/T. aralioides* and to *A. coerulea*, further confirming that the WGD in *N. nucifera* is also lineage-specific.

According to the synteny analysis, we investigated the *Ks* distributions for paralogous gene pairs that originated from the two WGD events (Fig. 2f). Using divergence time and median *Ks* values of syntenic blocks between *T. sinense* and *T. aralioides*, we estimated the synonymous substitutions per site per year as 3.25e−9 for the Trochodendrales (Additional file 1). Using this rate, we estimated that the α and β WGD events of Trochodendrales occurred at around 82 MYA and 59 MYA according to the median *Ks* of *T. sinense* paralogs, and 78 MYA and 54 MYA according to the median *Ks* of *T. aralioides* paralogs. This dating again suggests that the two WGDs occurred after the divergence of Trochodendrales from Proteales (*N. nucifera*).

Lastly, we performed integrated syntenic and phylogenomic analyses to confirm the timing of these WGDs in early diverging eudicots. We obtained anchor genes from the inter-genomic synteny blocks with ratio of 1:2:4 in *A. trichopoda*, *N. nucifera*, and *T. sinense*. All genes in the same sets of syntenic block were concatenated and used to construct phylogenetic trees. In total, we obtained 31 groups of concatenated genes (a total of 94 genes) and 87% of the trees based on the concatenated genes (Additional file 1: Fig. S10) supported the hypothesis that the WGDs in *N. nucifera* and *T. sinense* are lineage-specific.

### Occurrence of vessel elements in secondary xylem

**Fig. 3:**
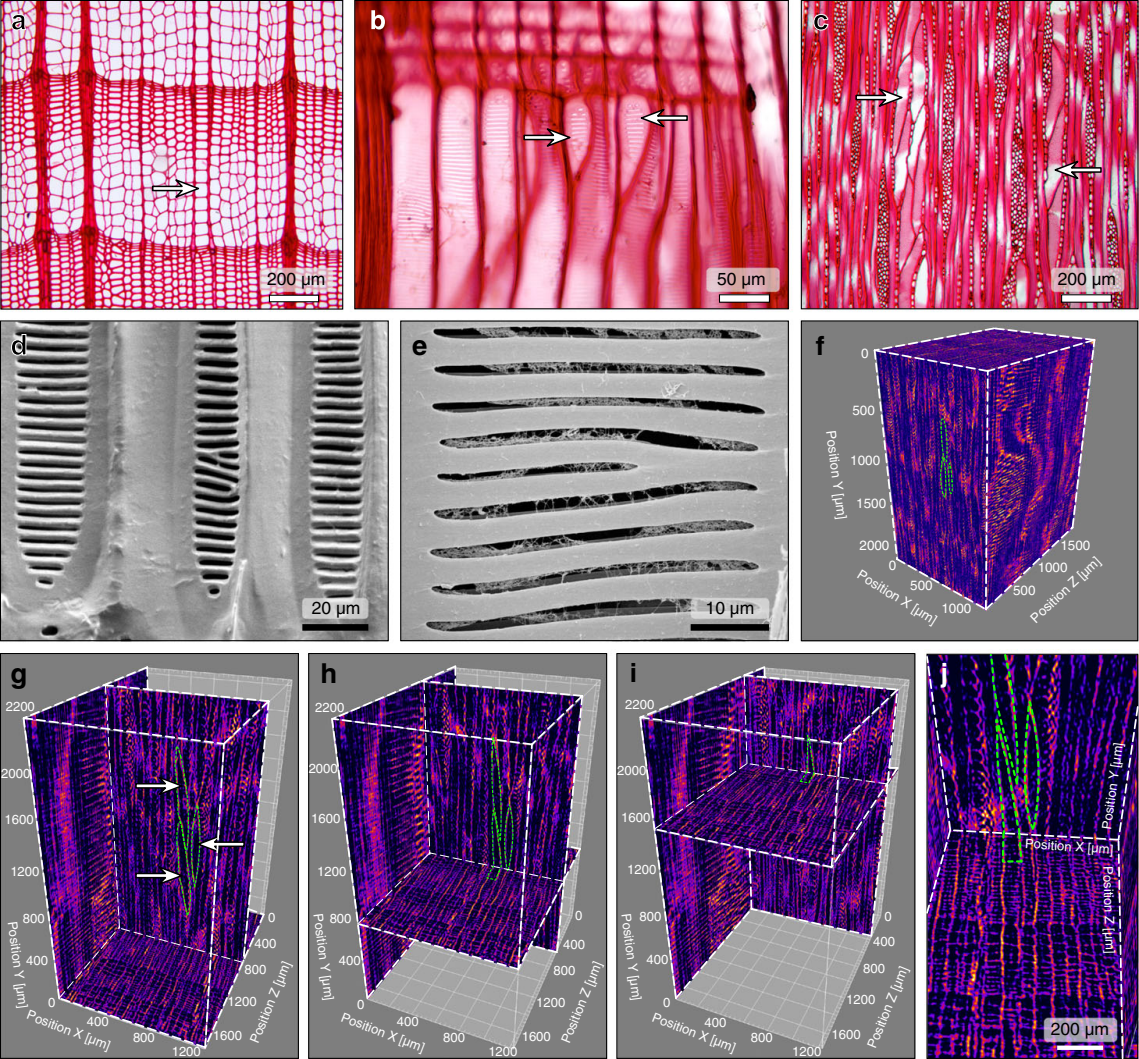
Occurrence of vessel elements in secondary xylem. **a**–**c** Light micrographs of wood sections from transverse, radial, and tangential sections of *T. sinense*, respectively. In the transverse sections, radial files of the peculiar cells are indicated by the arrows. Bar = 200 and 50 μm in **a**, **c**, and **b**. **d** Scanning electron micrographs of the radial surface in *T. sinense*, showing scalariform pits. Scale bar = 20 μm. **e** Scalariform pits with fibrillar mesh membrane remnants. Scale bar = 10 μm. **f**–**i** Three-dimensional (3D) images of X-ray computed microtomography (microCT). **f** Original rendering of the 3D microCT image in *T. sinense*. **g**–**i** Images of radial (*y*–*z*) and tangential (*x*–*y*) sections of 3D microCT image at 3 different longitudinal positions of transverse sections (*x*–*z*). Arrows in **g** indicate the peculiar cells appearing in the tangential section. **j** Enlargement of **h** showing the short and fusiform peculiar cells. Light green dotted lines represent these peculiar cells. Scale bars are indicated by axis labels in **f**–**h** and 200 μm in **j**

Under a light microscope, we found there was a distinct growth ring delineated by a band of latewood in the cross-section. Earlywood cells were larger and thin-walled, while those in latewood were smaller and thick-walled as reported by the previous study [17]. Occasionally, we noticed some peculiar cells occurred through the growth ring boundaries with much larger diameter (56.0 ± 0.82 μm ±, *n* = 35) than normal cells (44.7 ± 0.98 μm, *n* = 35) (Fig. 3a). In the radial section, the peculiar cells had oblique end-walls (Fig. 3b). In the tangential section, we can clearly observe there are two types of tracheary elements. Most of the cells appeared as fibrous with 3.0–4.8 mm in length while the peculiar cells were much shorter ranging from 308 to 597 μm and present as fusiform cells with many pits (Fig. 3c). Under scanning electron microscope (SEM), we observed the normal cells often have scalariform bordered pits on radial walls, sometimes with pit membrane remnants that are like a fibrillar mesh as reported previously (Fig. 3d, e). Using X-ray computed microtomography (microCT), we made a 3-dimensional reconstruction of different cells based on 1032 serial slice images and found the short and fusiform peculiar cells are sporadically present in *T. sinense* wood (Fig. 3f–j). Therefore, we considered the larger and shorter peculiar cells as vessel elements, supporting the idea that vessel elements are present in *T. sinense*, although its vessel elements are primitive [19, 20].

### The evolution of *VNS*, *LBD*, *R2R3-MYB*, *CesA*, and *PLCP* gene families which are involved in the formation of vessel elements

*AtVND7*, *AtLBD18/AtLBD30*, *AtMYB46*/*AtMYB83*, *AtCESA4* and *AtCESA8*, and *AtXCP1*/*AtXCP2*, belonging to the *VNS*, *LBD*, *R2R3-MYB*, *CesA*, and *PLCP* gene families [25, 45–48], respectively, are key genes involved in the development of vessel elements in *A. thaliana* [21, 49]*.* To identify the *T. sinense* genes orthologous to these genes, we explored the evolutionary history of *VNS*, *LBD*, *R2R3-MYB*, *CesA*, and *PLCP* gene families.

**Fig. 4:**
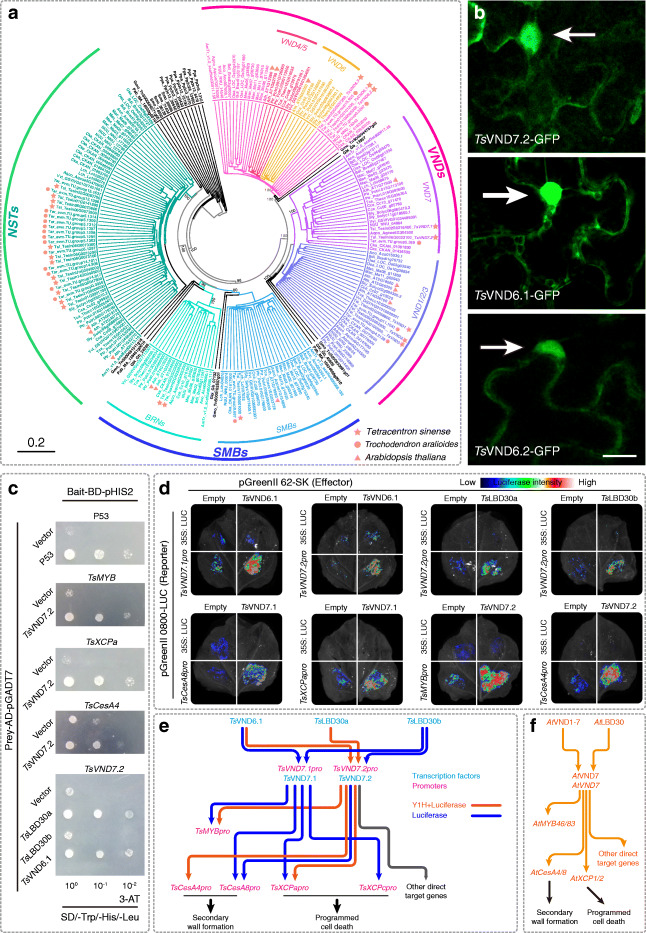
Regulatory relationships between TsVND7 and vessel-related genes in *T. sinense.* **a** Maximum likelihood tree of genes from the *VNS* gene family. Bootstrap percentages for the large clades are shown next to the nodes. Species name abbreviations given in Fig. S11 in additional file 1. Angiosperm sequences of different subfamilies are shown in different colors and the gymnosperm sequences are shown in black. **b** Confocal images of *Nicotiana benthamiana* leaves transiently transformed with *TsVND7.2-GFP*, *TsVND6.1-GFP*, and *TsVND6.2-GFP* under the control of the 35S promoter, bar = 10 μm. **c** Yeast one-hybrid assay showing that *Ts*VND6.1 and *Ts*LBD30a interact with the *TsVND7.2* promotor and *Ts*VND7.2 interacts with the *TsCesA4*, *TsMYB*, and *TsXCPa* promoters. **d** Luciferase activity assay showing that *Ts*VND6.1 interacts with promoters of *TsVND7.1* and *TsVND7.2*; *Ts*LBD30a and *Ts*LBD30b interact with the *TsVND7.2* promoter; *Ts*VND7.1 interacts with the *TsCesA8* and *TsXCPa* promoters; and *Ts*VND7.2 interacts with the *TsMYB* and *TsCesA4* promoters. **e** Diagram showing the interaction relationship determined in *Arabidopsis*. **f** Diagram showing the interaction relationship from the results of Y1H and luciferase activity assay in **c** and **d** and supplementary Fig. S13 b–d. The orange lines show the interactions evidenced by Y1H and luciferase activity assays, the green line shows the interactions evidenced by Y1H, and blue lines show the interactions evidenced by luciferase activity assay

The *VNS* gene family contains *VND*, *NST*, and *SMB* subfamilies [45]. As shown in the phylogenetic tree, genes homologous to *VND*, *NST*, and *SMB* each fell into different clades with high bootstrap values and each clade did not contain sequences from *Physcomitrella patens* (also known as *Physcomitrium patens*) and *Selaginella moellendorffii*, suggesting that the origin of these subfamilies occurred after the divergence of lycophytes. In the phylogenetic tree (Fig. 4a; Additional file 1: Fig. S11a), genes from the *VND* group clustered into two large clades: one including *A. thaliana VND4*–*VND6* and the other including *VND1*–*VND3* and *VND7*. Each of these clades contains sequences from gymnosperms (in black in the tree, Fig. 4a) and angiosperms, suggesting that the duplication leading to the ancestors of *VND4*–*VND6*, and *VND1*–*VND3* and *VND7* occurred before the divergence of gymnosperms and angiosperms. In the clade containing *VND1*–*VND3* and *VND7*, genes homologous to *VND1*–*VND3* and *VND7* each formed a subclade and each subclade only contained sequences from angiosperms, suggesting that the duplication leading to the origin of the *VND7* ancestor occurred after the divergence of angiosperms and gymnosperms. Sequences from the *VND7* clade were aligned and two diagnostic domains of *VND7* subfamily were found in all these sequences (Additional file 1: Fig. S11b), suggesting that they are bona fide *VND7* genes.

The transcription factors ASL19/LBD30 and ASL20/LBD18 are involved in a positive feedback loop to regulate *VND7* expression [27]. We identified 27 *LBD18/LBD30* genes in angiosperms. However, no *LBD18/LBD30* genes were identified in genomes of three gymnosperm species, one lycophyte, and one moss, suggesting that the origin of the *LBD18* subfamily may have occurred after the divergence of angiosperms and gymnosperms. In the ML tree (Additional file 1: Fig. S12a), the two genes (*AtLBD18* and *AtLBD30*) from *A. thaliana* formed one clade, suggesting that they originated from lineage-specific duplications. The two copies in *T. sinense* (Tesin01G0033700 and Tesin21G0059700) are putative orthologs to *A. thaliana AtLBD18* and *AtLBD30*.

In total, 87 *R2R3-MYB* genes were identified in the *T. sinense* genome. These sequences together with sequences from *A. thaliana* and *O. sativa* were subjected to phylogenetic analysis. As shown in the ML tree (Additional file 1: Fig. S12b), sequences from the three species fell into different clades. All subgroups (except for 2 subgroups only including sequences from *A. thaliana*) contain sequences from *A. thaliana/T. sinense* and *O. sativa*, suggesting these subgroups originated before the splitting of monocots and dicots. In subgroup MYB46 and MYB83, there are three *T. sinense* sequences (Tesin13G0114100, Tesin11G0137400, Tesin07G144300).

In the ML tree (Additional file 1: Fig. S12c), the *CesA* sequences of *P. patens* formed their own clade. CesAs in seed plants fell into six major clades, which were designated as six subfamilies: *CesA1*/*10*, *CesA3*, *CesA6*, *CesA4*, *CesA7*, and *CesA8*. Each subfamily included sequences from angiosperms and gymnosperms, indicating the occurrence of ancient gene duplication events before the divergence of angiosperms and gymnosperms. In the phylogenetic tree, subfamilies *CesA4*, *CesA7*, and *CesA8* clustered together and the remaining subfamilies clustered together. *CesA* sequences of *S. moellendorffii* fell into each of these two clades. All subfamilies, including subfamilies *CesA4* and *CesA8*, also contained only one *T. sinense* gene except for subfamily *CesA1/10*, which contained three *T. sinense* genes.

Congruent with previous studies [48], most *PLCP* genes fell into nine subfamilies (*SAG12*, *THI*, *CEP*, *XCP*, *RD21*, *XBCP3*, *RD19*, *ALP*, and *CTB*). SAG12, THI, CEP, XCP, RD21, and XBCP3 have been classified as L-like cathepsins, and RD19, ALP, and CTB have been classified as F, H, and B-like cathepsins [48], respectively. In the phylogenetic tree (Additional file 1: Fig. S12d, e), all subfamilies of L-like cathepsins clustered together while F, H, and B-like cathepsins were relatively distant from them. L, F, H, and B-like proteins appeared in the moss and lycophyte, suggesting that ancient duplications led to the origin of these subfamilies. As shown in the ML tree, *XCP* genes originated before the divergence of gymnosperms from *S. moellendorffii.*

### Nuclear localization and transcriptional regulatory network of *VND* genes

To investigate the intrinsic function of VNDs in *T. sinense*, we first analyzed their subcellular localization by transient expression. Like *Arabidopsis VND6* and *VND7*, *Ts*VND7.2-GFP, *Ts*VND6.1-GFP, and *Ts*VND6.2-GFP predominantly localize to the nucleus, as seen by co-localization assays with GPF fluorescence merged with a bright-field image (Fig. 4b; Additional file 1: Fig. S13a).

Yeast one-hybrid (Y1H) analysis was subsequently performed to determine the interaction between *Ts*VND7.1 and *Ts*VND7.2 and other genes. The yeast-carrying Bait-BD-pHIS2 vectors expressing the promoter of *TsVND7.1*, *TsVND7.2*, *TsMYB*, *TsCesA4*, *TsCesA8*, *TsXCPa*, or *TsXCPc* grew normally on selective medium without 3-AT but failed to grow with 3-AT, showing that no self-activation occurred (Additional file 1: Fig. S13b). The following tests were carried out by promoters fused to Bait-BD-pHIS2 co-transformed with transcription factors fused to Prey-AD-pGADT7. The results showed that *Ts*VND7.2 interacted with the promoters of *TsMYB*, *TsCesA4* and *TsXCPa*, and *TsVND7.2* can be regulated by *Ts*VND6.1 and *Ts*LBD30a (Fig. 4c). Conversely, *Ts*VND7.1 did not interact with the *TsMYB*, *TsCesA4*, *TsCesA8*, *TsXCPa*, *TsXCPb*, or *TsXCPc* promoters and was not regulated by *Ts*LBD30a. *Ts*VND7.2 also did not bind to the promoters of *TsCesA8*, *TsXCPb*, and *TsXCPc* (Additional file 1: Fig. S13c).

The interaction between *TsVND7.1/TsVND7.2* and other genes was further assessed using luciferase activity assays (Fig. 4d; Additional file 1: Fig. S13d). In these assays, *Ts*VND7.2 interacted with promoters of *TsMYB*, *TsCesA4*, *TsCesA8*, *TsXCPa* and *TsXCPc*, and *TsVND7.2* can be regulated by *Ts*VND6.1, *Ts*LBD30a, and *Ts*LBD30a. *TsVND7.1* also showed similar interactions with other genes except *TsLBD30a*, *TsCesA4*, and *TsXCPc*.

### Demographic history analysis of *T. sinense*

**Fig. 5:**
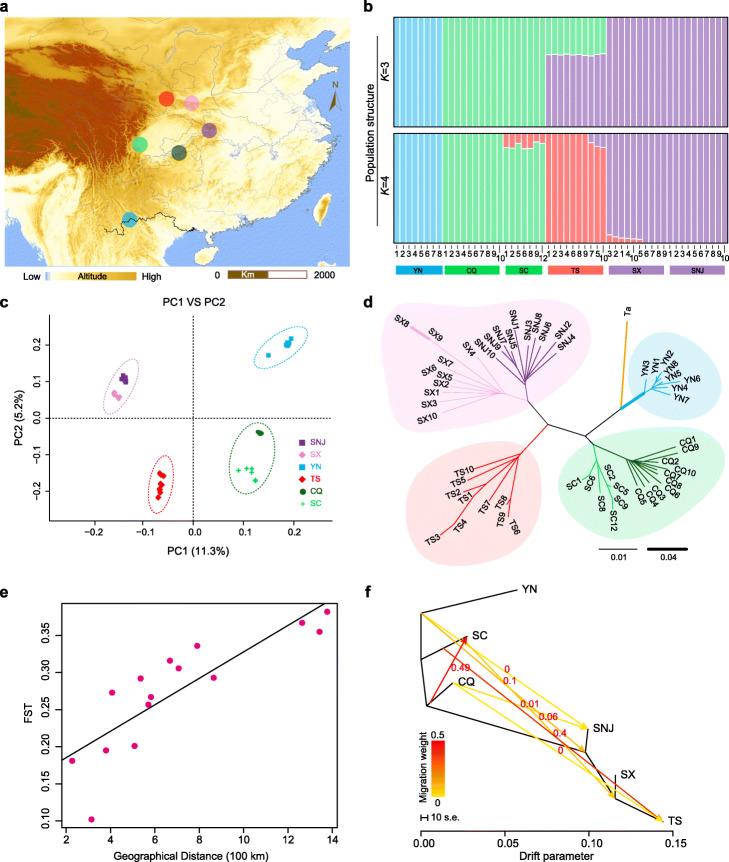
Phylogenetic relationships and population structure of *Tetracentron* populations. **a** Geographic distribution of the sampling locations. **b** Results of STRUCTURE analysis, showing four significant genetic clusters. **c** Principal component analysis (PCA), with the proportion of the variance explained being 11.3% for PC1 and 5.20% for PC2. **d** A neighbor-joining (NJ) phylogenetic tree. e Correlation between *F*st and geographic distance by Mantel test (*r* = 0.8417, *p* = 0.004). f Detection of gene flow between *Tetracentron* populations. Lines with arrows indicates gene flow and direction of gene flow. The color scale: red indicates strong gene flow and yellow indicates weak gene flow. The horizontal scale bar at the bottom (drift parameter) shows a tenfold average standard error of the entries in the sample covariance matrix

To explore the historical demographic fluctuations and present-day genetic diversity within this endemic species, we resequenced 55 individuals of *T. sinense* from a range-wide sampling from China (Fig. 5a). ADMIXTURE analysis [50] of Chinese samples revealed that the deepest splits occurred among the southwestern population (population YN), the populations near the Sichuan Basin in central China (represented by populations CQ from the Jinfo Mountains and SC from the Emei Mountains), and the populations from the North and East parts of central China (represented by populations SNJ from the Shennong Mountains and populations SX and TS from the Qingling Mountains) (Fig. 5b; Additional file 1: Fig. S14). When *K* = 4, the TS population from the Qinling Mountains in north-central China diverged from the third clusters and four ancestries of *T. sinense* occurred in China (Fig. 5b). Principal component analysis (PCA) and phylogenetic analysis also revealed four major clusters (Fig. 5c, d; Additional file 1: Fig. S14b, c).

*T. sinense* exhibited higher within-species genetic diversity than within-population level genetic diversity. The within-population genetic diversity of *T. sinense* (6.30–11.58e−3 for Watterson’s estimator (θ_w_) and 6.48–11.25e−3 for nucleotide diversity (π), Additional file 1: Table S3) was obviously low compared to the species level diversity. The genetic differentiation statistics (fixation index; *F*st) further showed that genetic differentiation between YN population and all other populations (0.293–0.382) was higher than those between others (most < 0.293), and the genetic differentiation between SX and SNJ population (0.102) was the lowest (Additional file 1: Table S4).

The Mantel test revealed a significant positive correlation between the genetic and geographic distance (*r* = 0.8417, *p* = 0.004, Fig. 5e). To estimate gene flow between populations, we performed Treemix v1.13. analysis [51], which suggested that the TS population from the Qinling Mountains in north-central China is the result of admixture between the SC population the near Sichuan Basin and the SX population from the Qinling Mountains in the north and east parts of central China (Fig. 5f). Gene flow is also observed in population SC and CQ. It is consistent with that they were in the same group according to phylogenetic, PCA, and structure analysis.

**Fig. 6:**
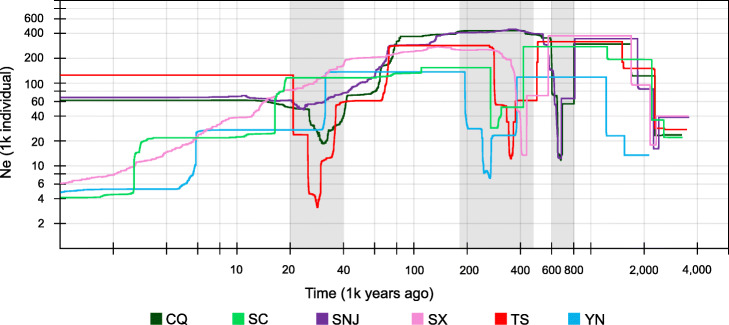
Demographic history of *T. sinense*. Stairway plot showing historical changes in effective population size (*y*-axis) for six populations (CQ, SC, SNJ, SX, TS, YN) with a generation time of 15 years. Gray shadows highlight bottlenecks

An analysis of demographic history using stairway plots revealed bottlenecks the populations may have undergone (Fig. 6), including one ranging 0.3–0.67 MYA, and a recent bottleneck around 0.03 MYA. For three populations (SC, SX, and YN), their effective population size is declining since they were affected by the Last Glacial Maximum and their extinction is underway.

## Discussion

### Polyploidization in the early evolution of eudicots

Previous studies suggested that Ranunculales, Proteales, Trochodendrales, and Buxales formed the grade of early diverging eudicots [6, 10, 11]. However, these analyses mainly relied on chloroplast and mitochondrial genes as well as a few nuclear ribosomal genes. It is particularly necessary to compare organellar gene-based phylogenetic estimates with topologies derived from analyses of low-copy nuclear genes [1]. The *T. sinense* genome provides an opportunity to resolve the relationships between lineages based on genome-wide low-copy nuclear genes. Our analysis based on 214 single-copy nuclear gene families (orthogroups) provides strong support for the conclusion that the Ranunculales, Proteales, and Trochodendrales are as successive sisters to the core eudicots. Within eudicots, the Ranunculales diverged at ~ 136 (129–140) MYA, followed by the Proteales ~ 131 (123–137) MYA, the Trochodendrales ~ 125 (117–132) MYA, and the core eudicots ~ 113 (105–123) MYA (Fig. 1). This estimate is generally consistent with several estimates from other relaxed molecular clock analyses based on DNA and/or genomic data [36, 52]. The narrow range of ages suggests a rapid diversification of the early eudicots. In Trochodendrales, the divergence time of *Tetracentron*–*Trochodendron* is approximately 31 MYA (Fig. 1; Additional file 1: Fig. S3). This is generally congruent with the age of the earliest reliable fossils of *Tetracentron* and *Trochodendron*, which date to the early middle Eocene epoch [30, 53].

Prevalent polyploidization events have been identified during the evolutionary history of angiosperms [54]. Previous studies demonstrated that the rise and eventual dominance of core eudicots might have benefited from the γ hexaploidization event in the most recent common ancestor of core eudicots [4]. Based on genomic investigations of *V. vinifera* [55] and *N. nucifera*, Ming et al. [13] proposed that the triplication event involved an initial tetraploidization event after the divergence of early diverging eudicots, then involved hybridization with a now-extinct species that branched off around the same time as, or earlier than *N. nucifera*. By contrast, a recent study using the *A. coerulea* genome [5] also proposed a hybrid origin of core eudicots, involving a tetraploidization event that occurred in the ancestor of all eudicots, then a hexaploidization in the ancestor of core eudicots. In this study, we performed comprehensive analyses including synteny, *Ks*, and phylogenomic analysis to clarify the nature of the γ event. Our results strongly suggested that the WGDs that occurred in *A. coerulea*, *N. nucifera*, and the two species of Trochodendrales are lineage-specific, and no WGD occurred in the common ancestor of all eudicots (Fig. 2g). The ancient WGD (α) in Trochodendrales dates to about 59–54 MYA, which coincides with the Cretaceous–Tertiary (KT) mass extinction event [56]. In fact, many independent WGDs have been identified in many lineages and have been proposed to have contributed to survival during this period of dramatic environmental changes [54, 56].

It is also worth noting that after the two successive WGDs in *T. sinense*, the genome structure remained well conserved. *Ks* analyses for orthologs between *T. sinense/T. aralioides* and *V. vinifera* showed the Trochodendrales lineage has the lowest nucleotide substitution rate among the studied species (Fig. 2e)*.* The lineage nucleotide substitution rate in *N. nucifera* is about 30% lower than that of *V. vinifera* [13]. We found that the nucleotide substitution rate of *T. sinense* is much lower than *N. nucifera*, since the peak of the *Ks* distribution of orthologs between *T. sinense* and *A. coerulea* is smaller than that of *N. nucifera* and *A. coerulea* (representing the same divergence event) (Fig. 2e). Together, these results indicate that the genome of *T. sinense* could serve as an exceptional comparative reference genome for investigating early evolution of eudicots.

Genomic analysis of gene families can shed light on the morphological characteristics shared by different clades. The early diverging lineages of eudicots tend to show some ancestral characteristics that differentiate them from the core eudicots. For example, the flowers of core eudicots are organized in a predictable manner with a stable number of parts (e.g., flowers with parts in five or multiples of five, a clear differentiation of sepals and petals), but in the Trochodendrales (like in some early angiosperms), the perianth is not differentiated into typical sepals and petals [8]. MADS-box (ABCE) genes are among the most important regulators of flower development. Analysis of the *Amborella* genome revealed that duplication and diversification of floral MADS-box genes likely occurred before the origin of extant angiosperms [40]. Our results showed that A-, B-, C-, E-function genes did not significantly expand or contract in the lineages leading to eudicots and core eudicots. The differences in flower structure between eudicots and core eudicots may be due to the differences between broadly overlapping ABCE gene expression patterns in early diverging eudicots and angiosperms, and strict spatially restricted ABCE gene expression in core eudicots, as suggested by some studies in Nymphaeales, Magnoliids, and Ranunculales [41, 57, 58]. Notably, in the lineage leading to core eudicots, the TM3 MADS-box gene family that includes the important flowering time gene *SOC1* [59] has expanded. In the genome of *T. sinense*, the *SVP* gene family also expanded rapidly. This family includes the MAD-box gene *AGL24*, which is not only involved in the flowering transition through activation of *SOC1* [59, 60] but also in flower development together with class A and E genes [61, 62]. The expanded *SVP* clades could be involved in the complex networks regulating flowering in *T. sinense*.

### Vessel elements in secondary xylem of *T. sinense* and the transcriptional regulation of *VND7*-related genes

The presence of vessels in the xylem of angiosperms and their absence in the xylem of gymnosperms (except for gnetophytes [15]) is one of the striking differences between these two large groups of plants. The Tetracentraceae (also as the Trochodendraceae), however, is one of several angiosperm families that have been described as lacking vessels. Carlquist [63] found porous pit membranes on the end walls of early wood tracheary elements and considered that tracheary elements in *T. sinense* are transitional. According to the study of Ren et al., vessel elements exist in *T. sinense*, but they are of a primitive type [19, 20]. Our light microscopy and SEM observations of slices of *T. sinense* wood and analyses of X-ray computed microtomography (microCT) (Fig. 3) provide strong evidence that there are two types of tracheary elements and some of the peculiar fusiform tracheary elements with obvious end-wall perforation plates in *T. sinense* are vessels.

*VND7* belongs to the *VND* subfamily of the *VNS* gene family and is thought to be the master regulator of vessel differentiation [21], as *VND7* can induce various cells to trans-differentiate into vessel elements in *Arabidopsis* and other species. Gene family analyses showed that *VND7* occurred in angiosperms including in *T. sinense* and *T. aralioides*, but not in gymnosperms, indicating that the duplication event leading to the origin of the *VND7* ancestor occurred after the divergence of angiosperms and gymnosperms and before the split of angiosperm lineages (Fig. 4a; Additional file 1: Fig. S11a). This is consistent with a previous study that found no orthologs of *VND7* or *VND1–3* in gymnosperms [15]. The gnetophyte *Gnetum montanum* contains vessel-like water-conducting cells. However, the lack of *VND7* in *G. montanum* suggested that different molecular mechanisms underpin the origin and development of vessels in *Gnetum* and angiosperms, and the vessels in *Gnetum* and angiosperms are convergent characters [15]. By contrast, based on the phylogenetic analysis of the *VNS* gene family, we identified *TsVND7.1* and *TsVND7.2* of *T. sinense* as orthologs of *A. thaliana VND7*. Multiple sequence alignments showed that TsVND7.1 and TsVND7.2 have the same seven conserved subdomains as other VNDs and the conserved sites important for the functions of VNDs (Additional file 1: Fig. S11b). A transient expression assay showed that VND-GFP fluorescence localized to the nuclei of plant cells (Fig. 4b; Additional file 1: Fig. S13a) implying that VNDs in *T. sinense* act as nuclear transcription factors, as in *A. thaliana*.

Xylem vessel formation is regulated by a complex transcriptional network. *VND7* transcription factors directly regulate many genes including *MYB46*/*MYB83*, *CESA4* and *CESA8*, and *XCP1*/*XCP2* [21–26]*.* These genes are involved in vessel element differentiation, specifically in secondary cell wall formation and PCD. *VND7* is also positively regulated by several genes including *LBD18/LBD30* and *VND1*–*VND5* [14, 27, 28]. Gene family analyses suggested that all subfamilies that these genes belong to originated before the divergence of the angiosperms. We found that two genes, *TsLBD30a* (Tesin01G0033700) and *TsLBD3b* (Tesin21G0059700), are orthologs of the *AtLBD18*/*30* gene pair; three *TsMYBs* (Tesin13G0114100, Tesin11G0137400 and Tesin07G0144300) were found to be orthologous to *MYB46*/*MYB83*; *TsCesA4* (Tesin16G0055000) and *TsCesA8* (Tesin01G0177100) are orthologs of *AtCesA4* and *AtCesA8*, respectively; and *TsXCPa/b/c* (Tesin02G0006000, Tesin24G0110100, and Tesin06G0166500) are orthologous to *AtXCP1* and *AtXCP2* (Additional file 1: Supplementary Notes and Fig. S12). To ascertain whether these genes can regulate or are regulated by TsVND7.1 and TsVND7.2, we performed yeast one-hybrid analysis and luciferase activity assay. Yeast one-hybrid analysis showed that TsVND6.1 and TsLBD30a proteins can bind to the *TsVND7*.2 promoter and TsVND7.2 can interact with the promoters of *TsMYB*, *TsCESA4*, and *TsXCPa*. Luciferase activity assays not only confirmed the interactions between *TsVND7.2* and relevant genes as shown by yeast one-hybrid, but also identified the interaction between TsVND7.2 and *TsLAB30b*/*TsCesA8* (Fig. 4e). Although the two techniques showed some differences, the main results of both techniques demonstrated that the regulatory relationships between *TsVND7* and vessel-related genes are similar to those in *A. thaliana* (Fig. 4f), supporting the anatomical observations that vessel elements are present in *T. sinense*.

### Demographic history of *T. sinense*

*T. sinense* was widespread in many parts of the Northern Hemisphere until the Pliocene epoch of the Quarternary period [30, 64–67], indicating that the present-day endemism of *T. sinense* in east Asian is refugial. Previous studies identified five or three refugia in China [31, 32]. Our ADMIXTURE analysis of whole genomic SNPs of Chinese samples revealed four major groups (Fig. 5b; Additional file 1: Fig. S14). In good agreement with the ADMIXTURE analysis, principal component analysis (PCA) and phylogenetic analysis also revealed the same four major clusters (Fig. 5c, d; Additional file 1: Fig. S14). Hence, our results suggested that in addition to the Southwestern China refuge [31], there were three refugia in central China: one in the Sichuan Basin corresponding to the area covered by the Jinfo and Emei Mountains, one in the east part including the Shennong Mountains, and one in the Qinling Mountains. These areas have long been regarded as important global Pleistocene glacial refugia and the areas around the Sichuan Basin of central China and the Shennong Mountains in the east of central China were also likely refugia for many other relict tree species such as *Cathaya*, *Cercidiphyllum*, *Ginkgo*, and *Taxus* [68–70].

The genetic diversity of species is the result of historical evolution and also the basis of evolution; moreover, sufficient genetic variability is required for endangered species to survive and adapt to changing environments. Nucleotide diversity analysis of *T. sinense* showed high within-species genetic diversity (Additional file 1: Table S3) and its genetic diversity is higher than those reported for the relict species *A. trichopoda* [40] and *Ginkgo biloba* [69]*.* The high genetic diversity detected in this tertiary relict species might reflect the accumulation of mutations over long evolutionary time scales and in populations experiencing geographical isolation and heterogeneous habitats. This high level of genetic diversity indicated that this endangered species maintains a relatively high evolutionary potential. Congruent with the previous study [32], we observed a low genetic diversity within populations and high genetic differentiation among populations of *T. sinense* (Additional file 1: Table S4). The small population effect and geographical isolation were proposed to the main factors [32]. A population graph inferred by Treemix indicated similar relationships between populations to those revealed in the NJ tree and low levels of gene flow except between population SC and TS (0.4). Gene flow was fewer between distant populations, as confirmed by the Mantel test between the genetic and geographic distance (*r* = 0.8417, *p* = 0.004).

An analysis of demographic history using the stairway plot method revealed that all populations have undergone multiple cycles of expansion and contraction. During the early Quaternary, all populations underwent contraction, followed by expansion. For all populations, two significant bottlenecks were identified (Fig. 6). The time of the more ancient bottleneck identified for two populations (CQ and SC) corresponds to the Naynayxungla glaciation (0.5–0.72 MYA, the most extensive glaciation in China [71]) and the time of the more ancient bottleneck of the other four population corresponds to the Guixiang glaciation (0.13–0.3 MYA [71]). The most recent bottleneck of all six populations occurred around 0.03 MYA, corresponding to the Last Glacial Maximum (0.001–0.007 MYA). For three populations (SC, SX, and YN), their effective population size is declining since they were affected by the Last Glacial Maximum and their extinction is likely underway. As ongoing effects of an expanding human population threaten *T. sinense* populations, conservation efforts should focus on preserving the genetic diversity of endemic *T. sinense* trees.

## Conclusions

*Tetracentron sinense* belongs to the angiosperm order Trochodendrales, which consists of only one family (Trochodendraceae) and two species (*Tetracentron sinense* and *Trochodendron aralioides*). Based on the *Tetracentron sinense* reference genome and the previously published genomes from other species, our phylogenomic analyses support that the Trochodendrales is the sister lineage to the core eudicots. The genomes of the two species of Trochodendrales also revealed two WGD events (α and β) in Trochodendrales at approximately 82 and 59 MYA. Based on our comprehensive analysis including syntenic, *Ks* distribution and phylogenomic analysis, we conclude that the Trochodendrales did not experience the γ event and the event may have only happened in core eudicots. Through light microscopy and SEM of slices of *T. sinense* wood and microCT, we found that vessel elements are present in *T. sinense.* Consistent with the anatomical results, the *T. sinense* genome contains two putative orthologs of *VND7*, a master regulator of angiosperm vessel formation, which appears to function in a similar transcriptional network in *T. sinense* and *Arabidopsis*. Population genomics analysis of individuals across the native range of *T. sinense* in China revealed that all the populations have undergone two bottlenecks and there is a high level of genetic diversity in the species. As *Tetracentron sinense* is a basal eudicot with unique tracheary elements, its genome provides a unique reference for inferring the early evolution of the eudicots and the mechanisms underlying the formation of vessel elements. The population genomics analysis facilitates the interpretation of their genetic diversity and geographic structure, thus providing genetic implications for future conservation.

## Methods

### Illumina short-read sequencing

The sequenced individual of *Tetracentron sinense* is growing in the Kunming Botanical Garden, Kunming, China. Genomic DNA was extracted from fresh leaves and purified using the Tiangen Isolation/Extraction/Purification Kit (Tiangen Biotech (Beijing) Co., Ltd.). Then, it was fragmented with a Covaris M220 Focused-ultrasonicator Instrument. Illumina PCR-free libraries with insert sizes of 300–500 bp were constructed using the NEBNext Ultra DNA library Pre Kit for Illumina sequencing. Then, 150-bp paired-end sequencing was performed using the Illumina HiSeq X Ten platform.

### Genome survey

Using Illumina short reads, *k*-mer distribution was estimated using JELLYFISH v2 [72]. The overall characteristics of the genome such as genome size, repeat contents, and heterozygosity rate were estimated using GCE software [73].

### PacBio library construction and sequencing

SMRTbell libraries were constructed according to the manufacturer’s protocol of SMRTbell Express Template Preparation Kit (Pacific Biosciences). Briefly, high-quality and high-molecular-weight genomic DNA was purified using the Mobio PowerClean Pro DNA Clean-Up Kit, and DNA quality was assessed by agarose gel electrophoresis and Thermo Fisher Scientific Qubit Fluorometry. Genomic DNA was further sheared to a size range of 15–50 kb using AMPure beads (Beckman Coulte) or g-TUBE (Covaris) and enzymatically repaired and converted into SMRTbell template libraries. Then, hairpin adapters were ligated after exonuclease digestion. The resulting SMRTbell templates were size-selected by Blue Pippin electrophoresis (Sage Sciences). The size-selected SMRTbell DNA fragments ranging from 15 to 50 kb were sequenced on a PacBio Sequel instrument using S/P2-C2 sequencing chemistry (21 cells, 14.7 million reads, ~ 100×).

### Hi-C library construction and sequencing

Hi-C was performed according to the following procedure. Young leaves were fixed with formaldehyde and lysed, and then the cross-linked DNA was digested with the HindIII restriction enzyme and the 5′ overhangs were biotinylated. After labeling with biotin-14-dCTP, the resulting free blunt ends were ligated again. Then, the purified DNA was treated to remove biotin from non-ligated DNA ends. For the fragmentation, DNA was then sheared with a Covaris M220 Focused-ultrasonicator Instrument. The sheared DNA was then repaired and biotin-containing fragments were isolated using streptavidin beads. A-tailing and adapters were ligated subsequently, and sequencing libraries were generated and sequenced on an Illumina HiSeq X Ten instrument with 150-bp paired-end reads.

### Genome assembly and pseudomolecule building

The primary assembly was performed with PacBio long reads using six different approaches, CANU (v0.1) [74], SMARTdenovo (v 0.2) (https://github.com/ruanjue/smartdenovo), WTDBG (v0.3) (https://github.com/ruanjue/wtdbg), SMARTdenovo after CANU correction (v0.4), and WTDBG2 after CANU correction (v0.6). Then, based on the assembled genome size, number of contigs, average contig size, and N50, assemblies from SMARTdenovo (v 0.2), and WTDBG2 after CANU correction (v0.6) were selected for merging using quickmerge [75] to improve the contiguity. Finally, the draft assembly was polished using PacBio long reads with Arrow software (https://github.com/PacificBiosciences/GenomicConsensus) and corrected using Illumina paired-end reads with Pilon software [76].

Hi-C read pairs were aligned to the draft assembly v1.0 using Juicer software [77]. The resulting contact matrices and draft assembly v1.0 were used for further Hi-C scaffolding with the 3D (3d-dna) pipeline [78]. This procedure includes correcting the mis-joins, scaffolding the input contigs, polishing the megascaffold, splitting the megascaffold into raw chromosomal scaffolds, and then sealing raw chromosomal scaffolds to yield chromosome-length scaffolds. After the first round of 3d-dna, we manually adjusted the assembly with Juicebox^54^. Furthermore, to improve the chromosome-scale assembly, we performed 3d-dna within each chromosome [78]. The chromosome-length assembly was further improved by manual adjustment with Juicebox [79], including correcting mis-joins, changing the order and orientation of draft scaffolds with translocation and inversion errors, and adjusting chromosome boundaries (Fig. S2). Finally, we performed LR-Gapcloser [33] with PacBio long reads to close gaps (in the assembly) two times, and pilon [76] with Illumina paired-end short reads five times to improve the assembly. Redundans was used to remove redundancy in the unplaced contigs [80].

To evaluate the quality of the assembled genome, we mapped the Illumina paired-end reads, PacBio long reads, and RNA-seq reads to the genome using BWA-MEM [81], Minimap2 [82], and hisat2, respectively. We also used BUSCO to examine the completeness of gene content with the Embryophyta odb9 database and default parameters [34]. The LAI program was used to assess the quality of the assembly [83].

### RNA library construction and sequencing

We performed RNA sequencing (RNA-seq) to improve the prediction of gene models. Different plant tissues from *T. sinense*, including bud, stem, leaf, fruit, phloem, and xylem, were collected and frozen in liquid nitrogen. Total RNA was extracted with the RNAprep Pure Plant Kit (Tiangen Biotech Co., Ltd.) according to the manufacturer’s instructions. Transcriptome libraries were constructed using NEBNext Ultra RNA Library Prep Kit for Illumina (New England Biolabs Inc.) following the manufacturer’s procedure. Then, 150-bp paired-end sequencing was performed using the Illumina HiSeq X Ten platform.

### Transcriptome assembly

Three approaches were used to assemble the transcriptome of *T. sinense*. First, RNA-seq reads from six selected tissues were de novo assembled into transcripts with Trinity [84]. In the second and third approaches, RNA-seq reads were first mapped to the assembled genome of *T. sinense* using hisat2 [85]. Then, they were assembled into transcripts using the genome-guided strategy by Trinity and StringTie [86]. Transcripts obtained from different approaches were then merged and redundant transcripts were removed with a cutoff of 95% in both identity and coverage with CD-HIT [87].

### Genome annotation

The library of repeat families was generated from our assembly using RepeatModeler [88]. Then with this repeat library, RepeatMasker was used to identify repetitive elements in the genomic sequences. Protein-coding gene prediction of the *T. sinense* genome was conducted by the MAKER genome annotation pipeline [89], integrating ab initio prediction with de novo assembled transcripts and protein homology data from *Nelumbo nucifera* and *Arabidopsis thaliana*. Gene functional annotation was performed using BLAT by searching against the Swiss-Prot, TrEMBL, NR, Pfam and eggNOG databases [90], and using InterProScan [91] by searching against protein databases of InterPro. The gene ontology and KEGG information for each gene model was extracted from Swiss-Prot, TrEMBL.

The tRNA genes were identified by tRNAscan-SE [92], rRNA genes were identified by RNAMMER [93], and non-coding RNAs (ncRNAs) genes were identified by searching against the Rfam database [94].

### Phylogenetic analysis and estimation of the divergence time of species

OrthoFinder v2.3.1 [35] was used to generate gene family (orthogroups) classifications from 19 species including two basal angiosperms (*Amborella trichopoda* and *Nymphaea colorata*), three magnoliids (*Liriodendron chinense*, *Cinnamomum kanehirae*, and *Litsea cubeba*), four monocots (*Musa acuminata*, *Ananas comosus*, *Oryza sativa*, and *Brachypodium distachyon*), four basal eudicots (*Aquilegia coerulea*, *Nelumbo nucifera*, *T. sinense*, and *Trochodendron aralioides*), and six eudicots (*Arabidopsis thaliana*, *Populus trichocarpa*, *Vitis vinifera*, *Theobroma cacao*, *Solanum lycopersicum*, and *Coffea canephora*). Orthologous groups (OGs) with single-copy genes were used in the phylogenetic analysis. Amino acid sequences from each OG were first aligned with MAFFT [95]; the alignments were then adjusted manually and ambiguous positions were deleted with TrimAI [96]. Then, the sequence alignments were concatenated. Finally, the concatenated alignment was used to construct a maximum likelihood (ML) phylogenetic tree using IQ-TREE software [97].

We estimated the divergence time of species using MCMCTree [37] and BEAST2 [38]. For the age estimation using MCMCTree, the tree was calibrated with angiosperm crown age (209 million years ago, MYA) [36], eudicot crown age range (127.9–139.4 MYA), and *Theobroma cacao–Arabidopsis thaliana* divergence range (87–95 MYA). The latter two calibrated times were obtained from TreeTime [98]. For the age estimation using BEAST [38], the earliest tricolpate pollen (~ 125 MYA) fossil associated with eudicots and the fossil (100.5 MYA) assigned to the Pentapetalae were used as calibration points. The angiosperm crown age of 209 MYA [36] was also used as a calibration point.

Expansion and contractions of gene families were determined using CAFE 2.2 (Computational Analysis of Gene Family Evolution) [39]. The program uses a birth and death process to model gene gain and loss over a phylogeny. Enrichment of KEGG and Gene Ontology terms for *T. sinense* or *T. aralioides* gene families that rapidly evolved (expanded or contracted rapidly) were performed using goseq [99].

### Whole-genome duplication analysis

We performed intragenomic comparisons for the two species of Trochodendraceae, *Aquilegia coerulea*, *Nelumbo nucifera*, and *Vitis vinifera*. We also performed intergenomic comparisons between *T. sinense* or *T. aralioides* and several species including two basal angiosperms (*Amborella trichopoda* and *Nymphaea colorata*), one magnoliid (*Cinnamomum kanehirae*), two early diverging eudicots (*A. coerulea*, *N. nucifera*), and one core eudicot (*Vitis vinifera*)*.* OrthoFinder v2.3.1 [35] was used to identify paralogous gene pairs within species and orthologs between species. Based on these paralogous and orthologous gene pairs, collinear blocks were identified using MCsanX [100]. Protein sequences of paralogous and orthologous gene pairs in collinear blocks were first aligned by MUSCLE [101]. The protein alignments were then converted to codon alignments by PAL2NAL [102]. Finally, *Ks* values were calculated using the Nei-Gojobori algorithm implemented in the PAML package [103].

To confirm the timing of these WGDs in early diverging eudicots, we further performed integrated syntenic and phylogenomic analyses. We obtained anchor genes from the inter-genomic synteny blocks present in ratios of 1:2:4 in *A. trichopoda*, *N. nucifera*, and *T. sinense*. All genes in the same sets of syntenic blocks were concatenated. These concatenated datasets were used to perform phylogenetic analysis. We then calculated the percentage of trees that supported the idea that the WGDs in *N. nucifera* and *T. sinense* are lineage-specific.

### Timing of WGD events

We first calculated the *Ks* value of the orthologs in syntenic regions between *T. sinense* and *T. aralioides* using KaKs_Caculator [104] based on the YN model. Given the median *Ks* value (0.2) of *T. sinense* and *T. aralioides* and their divergence date T (30.7 MYA), we calculated the synonymous substitutions per site per year (*r*) for Trochodendraceae as equaling 3.25e−9 (*r* = *Ks*/2 T).

We then applied the *r* value (3.25e−9) to estimate the time of the Trochodendraceae WGD events (*T* = *Ks*/2r). Since the median *Ks* values of paralogous pairs of *T. sinense* corresponding to the two WGDs were 0.532 and 0.382, we estimated that the two WGDs occurred around 82 and 59 MYA. Based on the median *Ks* of paralogous pairs of *T. aralioides* corresponding the two WGDs (0.507 and 0.348), we dated the two WGDs to around 78 and 54 MYA. The small difference in time of the two WGDs in the Trochodendraceae inferred from these two species may be due to a difference in their evolution rates.

### Light microscopy

Sectioning blocks of *T. sinense* [1 cm (*L*) × 1 cm (*R*) × 1 cm (*T*)] were cut with razor blades and then softened in 2% ethylenediamine at 60 °C for 2 days. Fifteen-micrometer-thick transverse, radial, and tangential sections were cut with a sliding microtome. Thereafter, sections were stained sequentially with 1% aqueous safranin solution, rinsed, and finally mounted beneath cover slides. Images were captured by a light microscope (Olympus BX50, Japan).

### Examination of secondary xylem with SEM

For scanning electron microscope (SEM) analysis, the stem materials from *T. sinense* were collected from the upper slope of Fenshuiling, Yunnan province, China. Stem samples were boiled in water and sectioned on a sliding microtome, then cut into pieces about 1 cm in length and fixed overnight in 2.5% glutaraldehyde, followed by washing with 0.1 M PBS (pH 7.2) three times. After sequential dehydration in 30%, 60%, 80%, 90%, 95%, and 100% ethyl alcohol (15 min each), the samples were incubated in liquid carbon dioxide at 0 °C and 20 °C sequentially (20 min each), then subjected to critical point drying at 35 °C for 15 min using a Tousimis autosamdri-825 critical point dryer. Vacuum coating was performed using a HITACHI MC 10003 Sputter Coater under 10 Pa and 1000 V for 2 min. The images were taken with an accelerating voltage of 5–10 kV using a Hitachi SU80202 SEM.

### MicroCT imaging

Wood blocks of *T. sinense* were imaged by X-ray microtomography using a Bruker Skyscan microCT system 1172 (Skyscan, Belgium) with a 20–100 kV, 10 W X-ray source. The microCT device was set to operate at a voltage of 33 kV and power of 7 W. The sample was scanned at a voxel size resolution of 1.792 μm. The software Skyscan’s NRecon program (Skyscan, Belgium) was used to perform the reconstruction of the projection images, resulting in a volume of 3304 × 3304 × 1806 μm from 1032 slice images with a resolution of 1888 × 1888 × 1086 pixels each. Imaris volume rendering and analysis software (Imaris × 64 9.0.1, BITPLANE, Oxford Instruments company) was used to render images from the three-dimensional data set.

### Gene family analysis

*AtVND7*, *AtLBD18/AtLBD30*, *AtMYB46*/*AtMYB83*, *AtCESA4*, *AtCESA8*, and *AtXCP1*/*AtXCP2* are key genes involved in the development of vessel elements in *A. thaliana* [21, 49]*.* These genes belong to the *VNS*, *LBD*, *R2R3-MYB*, *CesA*, and *PLCP* gene families [45–48], respectively. To investigate the evolutionary history of these gene families and identify the *T. sinense* genes orthologs, genes of *VNS*, *LBD*, *CesA*, and *PLCP* gene families from 22 representatives of major plant lineages, including 19 species of angiosperms used in the phylogenetic analysis of species, 3 gymnosperms (*Ginkgo biloba*, *Gnetum montanum*, and *Picea abies*), 1 lycophyte (*Selaginella moellendorffii*), and 1 moss (*Physcomitrella patens/ Physcomitrium patens*), were obtained using the hmmsearch command in the HMMER package [105] with their specific Pfam models (Extended Data Information). *R2R3-MYB* transcription factor homologs were obtained by a BLASTp search with e-value 1e−5 (Extended Data Information). Then, identical and defective sequences were identified and manually eliminated in BioEdit. Protein sequences or nucleotide sequences were aligned using MAFFT [95] and the alignment was manually adjusted in BioEdit. Maximum likelihood trees were constructed using IQ-TREE software [97].

### Plasmid construction and transient infiltration

To investigate the subcellular localization of the VNDs, we created transgenic plants expressing *At*VND6, *At*VND7, *Ts*VND6.1, *Ts*VND6.2, and *Ts*VND7.2 fusions with GFP. To this end, the *AtVND6*, *AtVND7*, *TsVND6.1*, *TsVND6.2*, and *TsVND7.2* coding region fragments were amplified from wild-type (WT) complementary DNA (cDNA) of *A. thaliana* and *T. sinense* by using PCR with gene-specific primer sets (Supplementary Table 4). After that, the cDNAs were each subcloned into a modified pCAMBIA 2300-GFP vector under the control of the 35S promoter to generate the *35S::AtVND6-GFP*, *35S::AtVND7-GFP*, *35S::TsVND6.1-GFP*, *35S::TsVND6.2-GFP*, and *35S::TsVND7.2-GFP* constructs. Transient infiltration of *Nicotiana benthamiana* was carried out using an *Agrobacterium tumefaciens* (GV3101). Images were captured using a Zeiss LSM 880 Airyscan (Carl Zeiss, Germany) for confocal microscopy. An argon laser (488-nm line) was used for GFP excitation and the emission was captured at 510–550 nm (green).

### Yeast one-hybrid analysis

Yeast one-hybrid analysis was performed to investigate the interactions between *Ts*VND6.1, *Ts*VND7.1, *Ts*VND7.2 or *Ts*LBD30a transcription factors and the promoters of *TsVND7.2*, *TsMYB*, *TsCesA4*, *TsCesA8*, *TsXCPa*, *TsXCPb*, and *TsXCPc* (about 500 bp in length from the translation start site). All the promoters and transcription factor genes were amplified by PCR using TransStart FastPfu DNA polymerase (TransGenBiotech, Beijing, China). Using BamHI/EcoRI or EcoRI/SacI digestion and T4 ligation reactions, the transcription factor genes were inserted into the AD vector (pGADT7, Takara Clontech Co., Ltd.) and the promoters were inserted into the BD vector (pHIS2, Takara Clontech Co., Ltd.). Then, the vectors were transformed into yeast strain Y187 and the transformed yeast was cultured for 2–3 days on SD/-Trp/-His double deficiency medium. Single clones harboring different vector combinations were transferred to SD/-THL medium containing 10, 20, 30, 40, 50, and 60 mM 3-AT and incubated at 30 °C for 0.5–8 h, and the color changes in the yeast cells were observed. All primers are shown in Table S5 in Additional file 1.

### Luciferase activity assay

To assay the activating activity of *Ts*VND6.1, *Ts*VND7.1, *Ts*VND7.2, *Ts*LBD30a, and *Ts*LBD30b to the *TsVND7.1, TsVND7.2*, *TsMYB*, *TsCesA4*, *TsCesA8*, *TsXCPa*, *TsXCPb*, and *TsXCPc* promoters, the pGreenII 0800-LUC reporter vector and pGreenII 62-SK effector vector (Biovector Science Lab) were used. The *TsVND7.1*, *TsVND7.2*, *TsMYB*, *TsCesA4*, *TsCesA8*, *TsXCPa*, *TsXCPb*, and *TsXCPc* promoters were cloned into pGreenII 0800 at the HindIII and BamHI sites to fuse them with the *LUC* reporter gene (*TsVND7.1*, *TsVND7.2*, *TsMYB*, *TsCesA4*, *TsCesA8*, *TsXCPa*, *TsXCPb*, or *TsXCPc* pro-LUC). The coding sequences for *Ts*VND6.1, *Ts*VND7.1, *Ts*VND7.2, *Ts*LBD30a, and *Ts*LBD30b were cloned into the pGreenII 62-SK vector at the EcoRI and HindIII sites. The recombinant vectors were co-transformed into *A. tumefaciens* GV3101. After centrifugation, the bacteria were collected and resuspended at A_600_ = 0.6 in infection solution (10 mM MES, 10 mM MgCl_2_, and 200 μM acetosyringone) for infiltration. The prepared suspensions were infiltrated into *N. benthamiana* leaves and grown in the dark for 48 h. The leaves were then sprayed with luciferin and kept in the dark for 10 min to quench the fluorescence. A CCD imaging apparatus (NightOWL LB 983 NC100, Berthold Technologies GmbH & Co. KG, Bad Wildbad) was used for image capture.

### Resequencing

DNA of 55 individuals from 6 populations, which represent most of the known distribution of *T. sinense* trees, was extracted and sequenced using Illumina technology. Raw data were filtered using Fastq [106]. Clean reads were aligned to the *T. sinense* genome using BWA-MEM [81]. Duplicate reads were marked with sambamba-markdup [107]. FreeBayes [108] was used to conduct SNP calling.

Ancestry estimation of individuals was inferred using ADMIXTURE [50] with different values of *K* (*K* = 1 to 10). We performed 5-fold cross-validation to determine the most likely number of clusters. The GCTA tool [109] was used to generate genetic relationship matrix (GRM) files and the GRM file was used to compute principal components (PCs). We constructed a neighbor-joining (NJ) phylogenetic tree using MEGA7 [110] based on the whole-genome SNPs of the 55 individuals. Branch support was estimated from 1000 bootstrap replicates. *T. aralioides* was used as an outgroup.

Population genetic parameters, including nucleotide diversity (π) [111] and the Watterson estimator (θw) [112], were used to measure the degree of variability within a population or species and Fst (fixation index) was used to measure the degree of genetic differentiation, which was calculated using the thetaStat command of ANGSD softpackage [113] over non-overlapping 20-kb windows. To estimate gene flow between populations, we performed Treemix v1.13 on the SNPs with the settings -se -bootstrap -k 500 -m, and values for (-m) ranging from 1 to 10. We applied the stairway plot [114] to the whole-genome sequences of nine populations to infer the historical effective population size. The estimated generation time and mutation rate were set to 15 and 2.745e−9, respectively.

## Supplementary Information

**Additional file 1. **Supplementary Notes, Supplementary Figs. S1–S14, and Supplementary Tables S1–S5.

**Additional file 2.** Review history.

## Data Availability

All raw sequencing reads generated in this study have been deposited to the NCBI Sequence Read Archive (https://www.ncbi.nlm.nih.gov/sra) with Bioproject accession number PRJNA625382 (https://www.ncbi.nlm.nih.gov/bioproject/PRJNA625382) [115]. The genome assembly sequences and gene annotations have been deposited at DDBJ/ENA/GenBank under the accession JABCRI010000000. Original images of *T. sinense* captured from field and original figures captured from microCT used for cell parameters analysis have been deposited to the figshare online database (10.6084/m9.figshare.13159991.v2) [116].
