# Supplementary material for: The Tetracentron genome provides insight into the early evolution of eudicots and the formation of vessel elements
Source: Genome Biol. 2020 Dec 2;21:291. doi: 10.1186/s13059-020-02198-7 (PMC7709256; doi:10.1186/s13059-020-02198-7)
Supplement: Supplementary file 1 — Additional file 1. Supplementary Notes, Supplementary Figs. S1–S14, and Supplementary Tables S1–S5. [file 13059_2020_2198_MOESM1_ESM.docx]

Supplementary Information for

**The *Tetracentron* genome provides insight into the** **early evolution of eudicots and the formation of** **vessel elements**

***This file includes:***

**Supplementary Methods and Notes**

**Full Reference List**

**Supplementary Figures**

**Fig. S1** The 17-mer distribution of Illumina short reads in *T. sinense*.

**Fig. S2** Hi-C Interaction heatmap of *T. sinense* reference genome.

**Fig. S3** Time tree of 214 single-copy orthologs in nineteen species.

**Fig. S4** Estimation of gene family expansion and contraction on each evolutionary branch.

**Fig. S5** Gene family expansion and contraction.

**Fig. S6** The enrichment of KEGG for the *T. sinense* rapidly expanded gene families.

**Fig. S7** Synteny analysis within *T. aralioides* and between *T. aralioides* and *T. sinense*.

**Fig. S8** Synteny analysis *T. sinense*/*T. aralioides* and *A. trichopoda*, *N. colorata* and *C. kanehirae.*

**Fig. S9** Synteny analyses between *T. sinense*/*T. aralioides* and *A. coerulea*, *N. nucifera* and *V. vinifera*.

**Fig. S10** Phylogenomic analysis of anchor genes.

**Fig. S11** Phylogenetic analysis of VNS gene family and the amino-acid sequence alignment of VND7 proteins.

**Fig. S12** Maximum likelihood tree of genes from the *LBD*, *MYB*, *CesA* and *PLCP* gene families.

**Fig. S13** The cellular localization and function of *VND* genes

**Fig. S14** ADMIXTURE and principal component analysis of *Tetracentron sinense*.

**Supplementary Tables**

**Table S1:** The statistics of repetitive sequences

**Table S2:** GO term enrichment of *T. sinense* rapid evolved gene families.

**Table S3:** Sample size and genetic diversity of different populations of *T. sinense*.

**Table S4:** Matrix of pairwise *Fst* value of six *T. sinense* populations.

**Table S5:** The primer used in this study.

**Supplementary Notes**

**Table of Contents**

1. [Genome size and heterozygosity estimation 4](#_Toc49608570)

[2. Genome annotation 5](#_Toc49608571)

[3. Estimation of the divergence time of species 6](#_Toc49608572)

[4. Timing of WGD events. 7](#_Toc49608573)

[5. Resequencing 8](#_Toc49608574)

[5.1 Samples used for resequencing 8](#_Toc49608575)

[5.2 SNP calling and quality control of SNPs 8](#_Toc49608576)

[5.3 Population genetic structure analysis and phylogenetic analysis 9](#_Toc49608577)

[5.4 Inference of demographic history using stairway plot. 10](#_Toc49608578)

[6. Gene families involved in the formation of tracheary elements 10](#_Toc49608579)

[6.1 *VNS* genes 12](#_Toc49608580)

[5.1.1 Identification and phylogenetic analysis of the VNS gene family 12](#_Toc49608581)

[6.1.2 VNS genes in T. sinense and gene family evolution 13](#_Toc49608582)

[6.2 *LBD* genes 14](#_Toc49608583)

[6.2.1 Identification and phylogenetic analysis of LBD genes 14](#_Toc49608584)

[6.2.2 LBD genes in T. sinense and gene family evolution 15](#_Toc49608585)

[6.3 Homologous genes to *A*. *thaliana* *Myb46* and *Myb83* 15](#_Toc49608586)

[6.3.1 Identification and phylogenetic analysis of MYB genes 16](#_Toc49608587)

[6.3.2 MYB genes in T. sinense and gene family evolution 17](#_Toc49608588)

[6.4 Cellulose synthase (CesA) family 18](#_Toc49608589)

[6.4.1 Identification and phylogenetic analysis of CesAs 18](#_Toc49608590)

[6.4.2 CesA genes in T. sinense and gene family evolution 18](#_Toc49608591)

[6.5 Papain-like cysteine protease (PLCP) gene family 19](#_Toc49608592)

[6.5.1 Identification and phylogenetic analysis of the PLCP gene family 20](#_Toc49608593)

[6.5.2 PLCP genes in T. sinense and gene family evolution 20](#_Toc49608594)

**1. Genome size and heterozygosity estimation**

A k-mer refers to an artificial sequence division of K nucleotides iteratively from sequencing reads. With the assumption that k-mers are uniquely in the ideal reference genome, they should be present only once in the genome sequence. If a large fraction of k-mers occur c times, we can estimate the sequencing coverage to be approximately c [1]. Therefore, k-mers frequencies can reflect the coverage of the genome. Such k-mer frequencies from sequencing data are used to estimate the genome size as well as other genomic characteristics such as repeat structure and heterozygous rate [2]. Here, Jellyfish v2 [1] was first used to count the number of the occurrence of k-mers based on Illumina paired-end reads. The results showed that the total number of 17-nucleotide k-mer is 62,427,300,786. By plotting the frequency of a given k-mer (k-mer depth) against the total number of k-mer with a given frequency, we found that the peak depth was 58 (Fig. S1) which can be used as the expected k-mer coverage depth. To estimate the expected k-mer coverage depth more accurately, we used the float precision estimation developed by Liu et al. (2012) which was performed in GCE software [2]. Then the genome size can be estimated to be approximately 1.12 Gb according to the formula that Genome_size = the total number of k-mer/ the expected k-mer coverage depth. In addition to the major peak observed from the distribution of k-mer occurrence, a secondary peak was also detected around half of the depth of the major peak. This peak reflected heterozygous genomic regions of the *T. sinense*. Since k-mers of two separate alleles in heterozygous regions are not identical, the heterozygous k-mers have only half of the expected coverage depth. This secondary peak corresponds to a peak depth of 29. The heterozygosity was estimated to be approximately 0.22% according to the formula that heterozygosity = a_1/2_/K (2- a_1/2_) (where the K is the k-mer size, and the a_1/2_ is the ratio of k-mer species with genomic frequency 1/2). Both genome size and heterozygous rate were calculated using GCE software [2]. In the GCE software, float precision estimation and proper treatment of sequencing error and coverage bias were used to improve the estimation accuracy.

2. Genome annotation

The library of repeat families was generated from our assembly using RepeatModeler [3]. Then with this repeat library, RepeatMasker was used to identify repetitive elements in the genomic sequences. The MAKER genome annotation pipeline was used for protein-coding gene prediction of the *T. sinense* genome [4]. This pipeline used a combined strategy of *ab initio* prediction, protein homology-based prediction, and transcriptome-based prediction. Gene annotation was performed on the repetitive elements masked genome. For *ab initio* gene prediction, AUGUSTUS was used for gene model prediction [5]. For the protein homology-based prediction, protein sequences from *Nelumbo nucifera* and *Arabidopsis thaliana* were downloaded and redundant sequences with > 95% identity and coverage were removed. A total of 54,731 protein sequences were aligned to the repeat-masked *T. sinense* genome with BLASTX. For the RNA-seq-based prediction, *de novo* assembled transcripts (233,285) were mapped to the *T. sinense* genome with BLASTN. After filtering and clustering predictions and alignments, Exonerate v2.4.0 was used to realign and polish matching and highly similar mRNAs and proteins to the input genomic sequence. Then, MAKER synthesized information from the polished and clustered transcripts and protein alignments to produce a hints file for annotations. Finally, information from three approaches was integrated by AUGUSTUS to generate the final gene models and AED (Annotation Edit Distance) scores were calculated for each of the predicted genes as part of the MAKER pipeline to assess the quality of the gene annotation. Gene functional annotation was performed using BLAT by searching against the Swiss-Prot, TrEMBL, NR, Pfam and eggNOG databases [6], and using InterProScan [7] by searching against protein databases of InterPro. The gene ontology and KEGG information for each gene model was extracted from Swiss-Prot, TrEMBL.

3. Estimation of the divergence time of species

We estimated the divergence time of species using the MCMCTree program [8] and the BEAST2 program [9]. MCMCTree performs Bayesian estimation of species divergence times using soft fossil constraints under various molecular clock models. The input tree of the program was the ML tree estimated with 308 single-copy genes generated by IQ-TREE software [10]. The tree was calibrated with angiosperm crown age (209 million years ago, MYA) [11], eudicot crown age range (127.9–139.4 MYA), and *Theobroma cacao–Arabidopsis thaliana* divergence range (87–95 MYA). The latter two calibrated times were obtained from TreeTime [12]. In addition, a sequence alignment used to construct the ML tree and a control file (called mcmctree.ctl) that contains the instructions for the program were provided for the program. The MCMCTree program discarded the first 100,000 iterations as burn-in, and then sampled every 200 iterations until it has gathered 10,000 samples. For the age estimation using BEAST, the data were the nucleotide sequences of the 308 single-copy genes used in the phylogeny construction concatenated in a single alignment. We used a calibrated Yule model with a relaxed molecular clock and nucleotide substitution model GTR+I+Г. The earliest tricolpate pollen (~125 MYA) fossil associated with eudicots and the fossil (100.5 MYA) assigned to the Pentapetalae were used as calibration points. The angiosperm crown age of 209 MYA^9^ was also used as a calibration point. Two independent replications each with 80,000,000 generations were run with sampling every 1,000 generations. The stationarity of the chains and convergence of the two runs was monitored by Tracer v. 1.5. LogCombiner was used to combine sampled trees from two independent runs, with the first 20,000 trees being discarded as burn-in. TreeAnnotator was used to calculate clade posterior probabilities and 95% credible intervals.

The estimated age of the *Tetracentron*–*Trochodendron* divergence based on MCMCTREE (30 MYA, 12–88 MYA 95% highest posterior density) is generally congruent with that based on BEAST (31 MYA). Both methods estimated that the divergence time of *Tetracentron* and *Trochodendron* is 31 MYA (Fig. 1 and Fig. S3).

4. Timing of WGD events

To estimate the time of the WGD events in *T. sinense* and *T. aralioides*, *Ks* values of *T. sinense* or *T. aralioides* syntenic block genes were calculated using YN model in KaKs_calculator v2.0. We then plotted the ortholog *Ks* distributions between *T. sinense* or *T. aralioides* and *V. vinifera.* We observed that *T. sinense* and *T. aralioides* had a slower substitution rate than that of *V. vinifera*, because the *Ks* values between genome-wide *T. sinense–V. vinifera* or *T. aralioides–V. vinifera* syntelog pairs are smaller than those among triplicated *V. vinifera* genes. A previous study demonstrated that *Nelumbo nucifera*, belonging to the basal eudicots, has a much slower nucleotide substitution rate than that of *V. vinifera* [13]. Therefore, we concluded that species from other lineages are not appropriate for estimating the substitution rate in the *T. sinense* lineage. Instead, we estimated the average evolutionary rate for the Trochodendraceae of the Trochodendrales using *T. sinense* and *T. aralioides*. We first obtained the orthologs at syntenic regions between *T. sinense* and *T. aralioides* and then calculated the *Ks* value of these orthologs using KaKs_Caculator [14] based on the YN model. The divergence time of 30.7 MYA between *T. sinense* and *T. aralioides* was obtained based on our divergence estimation using BEAST. Given the median *Ks* value (0.2) of *T. sinense* and *T. aralioides* and their divergence date T (30.7 MYA), we calculated the synonymous substitutions per site per year (r) for Trochodendraceae as equaling 3.25e-9 (r = *Ks* / 2T). This substitution rate was similar to that of many woody plants, such as the *Liriodendron* synonymous substitution rate of 3.02e-9 and *Populus* synonymous substitution rate of 2.5e-9 [15].

We then applied the r value (3.25e-9) to estimate the time of the Trochodendraceae WGD events (T = *Ks* / 2r). Since the median *Ks* values of paralogous pairs of *T. sinense* corresponding to the two WGDs were 0.532 and 0.382, we estimated that the two WGDs occurred around 82 and 59 MYA. Based on the median *Ks* of paralogous pairs of *T. aralioides* corresponding the two WGDs (0.507 and 0.348), we dated the *T. aralioides* WGDs to around 78 and 54 MYA. The small difference of the two WGDs in the Trochodendraceae inferred from these two species may be due to the difference in their evolution rates. Based on the fact that the *Ks* between genome-wide *T. aralioides–V. vinifera* syntelog pairs are smaller than that of *T. sinense–V. vinifera*, we can conclude that the evolution rate of *T. aralioides* is lower than that of *T. sinense*.

5. Resequencing

5.1 Samples used for resequencing

According to the fossil records, *T. sinense* was once widely distributed in the Northern Hemisphere before the Quaternary period. However, it is now currently restricted to East Asia from China to Northeastern India and is found in disjunct populations. In China, it mainly occurs in southwestern and central China in Yunnan, Sichuan, Chongqing, Gansu, Hubei, Shangxi, Hunan, and Guizhou provinces. We sampled one population from each of six provinces, which represent most of the known distribution of *T. sinense* trees. In each population, we sampled 7–12 trees with a diameter at breast height (DBH) larger than 30 cm.

5.2 SNP calling and quality control of SNPs

To generate high-quality clean reads, raw data were filtered using Fastp to remove adapters, reads with more than one unidentified base (N) and reads shorter than 60; the filtering also removed bases in the sliding window with mean quality below the threshold (default is Q20) (fastp --cut_by_quality3 --cut_by_quality5 --n_base_limit 1 --length_required 60 --correction). In total, whole-genome resequencing of 55 plants generated from 20 Gb to 30 Gb nucleotides of sequences with an average depth of 20x, ranging from 17- to 25-fold for each sample. Clean reads were aligned to the *T. sinense* genome using BWA-MEM [16]. Samples could be aligned to the reference genome with an average mapping ratio more than 91% (reads). Duplicate reads were marked with sambamba-markdup [17]. In all of the subsequent analyses, bases with quality below 20 and reads with mapping quality lower than 30, sites with depth lower than 300 and higher than 2000 were excluded and non-chromosomal sequences were also excluded, The FreeBayes [18] was used to conduct SNP calling (freebayes --standard-filters --min-repeat-entropy 0 --min-alternate-count 2 --ploidy 2 --use-best-n-alleles 10 --genotype-qualities --max-coverage 9000). In total, we obtained 82,036,751 raw SNPs. Then, we filtered the raw SNP set using vcftools (vcftools --minGQ 20 --minDP 3 --min-alleles 2 --max-alleles 2 --positions $sites --remove-indels; vcftools --max-missing 0.8; vcftools --maf 0.05). Sites with genotype quality lower than 20 and depth lower than 3 were defined as missing sites. Sites of non-SNP, nonbialleles, missing rate over 20%, and minor allele frequency (MAF) less than 0.05 were removed from subsequent analyses. Finally, we identified a total of 11,800,064 high-quality SNPs.

5.3 Population genetic structure analysis and phylogenetic analysis

Linkage sites were filtered with Plink [19] (--indep-pairwise 50 10 0.08). Plink was also used to transform the variant call format (VCF) file into bed format file. Ancestry estimation of individuals was inferred using ADMIXTURE [20] with different values of K (K=1 to 10). A good value of K will exhibit a low cross-validation (CV) error compared to other K values. We performed 5-fold cross-validation to determine the most likely number of clusters. The results showed that the CV value for K=4 is the lowest. So, K=4 is a sensible modeling choice. Principal component analysis (PCA) was conducted to study the relatedness and clustering among populations or samples. We performed PCA according to the following procedures. The GCTA tool [21] was used to generate genetic relationship matrix (GRM) files and the GRM file was used to compute principal components (PCs). We constructed a neighbor-joining (NJ) phylogenetic tree using MEGA7 [22] based on the whole genome SNPs of the 55 individuals. Branch support was estimated from 1000 bootstrap replicates. *T. aralioides* was used as an outgroup.

Population genetic parameters, including nucleotide diversity (π) [23] and the Watterson estimator (θw) [24] were used to measure the degree of variability within a population or species and Fst (fixation index) was used to measure the degree of genetic differentiation, which was calculated using the thetaStat command of ANGSD softpackage [23] over non-overlapping 20-kb windows.

5.4 Inference of demographic history using stairway plot

We restricted our demographic analysis to genomic regions that were at least 5 kb away from any coding region. Ancestral sequences used to estimate the unfold site frequency spectrum (SFS) were constructed by IQ-TREE. The realSFS command of ANGSD software [25] was used to estimate the unfold site frequency spectrum (SFS) from the sample allele frequency likelihoods for each site. Using unfold SFS, we applied the stairway plot [26] to the whole-genome sequences of nine populations to infer the historical effective population size. The estimated generation time and mutation rate were set to 15 and 2.74e-9, respectively, which we obtained based on r=*Ks*/2t (the median *Ks* value of orthologs between *T. sinense* and *T. aralioides* and (0.187) and the divergence time between them (31MYA).

6. Gene families involved in the formation of tracheary elements

NAC proteins belong to a large transcription factor family and have a characteristic, conserved NAC domain in the N-terminal. In *Arabidopsis thaliana*, about 108 NAC genes have been identified [27]. The *VNS* (*VND*-, *NST*/*SND*-, *SMB*-related proteins) gene family belongs to the large *NAC* gene superfamily and contains three subfamilies (*VND*, *NST* and *SMB*), each containing genes homologous to the *VND*, *NST* and *SMB* genes of *A. thaliana* with different functions in many plant species [28]. There are seven *VND* genes (*VND1*–*VND7*), three *NST*/*SND* genes (*NST1*, *NST2*, and *AtNST3*/*SND1*), and three SMB genes (*SMB*, *BRN1*, and *BRN2*) in the *A. thaliana* genome [27].

Xylem vessel formation is regulated by a complex transcriptional network and the *VND* subfamily members plays critical roles in regulating vessel element formation [29]. Overexpression of *VND6* and *VND7* can induce differentiation of various types of cells into metaxylem and protoxylem-like vessel elements [29], suggesting that these genes are transcriptional switches of vessel formation [29, 30]. VND6 is a direct regulator of genes related to secondary wall formation and programmed cell death (PCD) [31]. Studies further suggested that *VND7* directly regulates the expression of a broad range of genes involved in xylem vessel element formation, such as *MYB46* and *MYB83*, transcription factor genes that function as master regulators of secondary wall formation-related genes [32, 33], *IRX1/CesA4* and *IRX5*/*CesA8,* gene*s* that participate the biosynthesis of cellulose of xylem secondary wall, and *XCP1* and *XCP2*, genes related to PCD [34]. The expression of *VND7* is also tightly regulated by other transcription factors. Electrophoretic mobility shift assays revealed that *VND1*–*VND7* bound to the *VND7* promoter region and *VND7* is the direct target of these transcription factors. The transcription factors *ASL19/LBD30* and *ASL20/LBD18* are involved in a positive feedback loop to regulate VND7 expression [35].

The *NST* subfamily is phylogenetically closest to the *VND* subfamily [27]. *NST1* is a key transcriptional switch of secondary cell wall biosynthesis in *A.* *thaliana* fiber cells [36]. *SND1* (also named NST3 and ANAC012) and *NST1* function redundantly in the regulation of secondary wall biosynthesis in fibers [37]. Overexpression of *NST1* and *SND1*/*NST3* induced ectopic lignified secondary cell wall thickening in various tissues [38, 39] . Double knockouts mutations of the *nst1* and *snd1*/*nst3*/*anac012* genes resulted in defects of secondary cell wall thickenings of fiber cells [37, 39]. These results indicated that *NST1* and *NST3* function in secondary wall formation of vascular tissues.

The *SMB* subfamily is also phylogenetically closely related to the *VND* gene subfamily. *SMB*, *BRN1*, and *BRN2* of this subfamily redundantly regulate the cellular maturation of the root cap [40, 41]. In single *smb-3* mutants, lateral cap cells fail to detach from the root. In *brn1-1* *brn2-1* double mutants, columella cells fail to detach. In triple mutants of these three genes, cells fail to mature in all parts of the root cap [41].

6.1 *VNS* genes

6.1.1 Identification and phylogenetic analysis of the VNS gene family

NAC proteins are characterized by a conserved N-terminal NAC domain and a divergent C-terminal activation domain [42]. All *VNS* genes contain seven conserved subdomains, five of which are in the conserved NAC domain and two of which are in the divergent C-terminal region [42, 43]. We first obtained all the *NAC* protein sequences using the hmmsearch command in the HMMER package [44], then we obtained all the *VNS* gene sequences on the basis of the phylogenetic tree. We used hmmsearch command to search the NAC-domain profile (PF02365) against the genomes of representatives of major plant lineages, including 17 species of angiosperms used in the phylogenetic analysis of species, 3 gymnosperms (*Ginkgo biloba*, *Gnetum* *montanum*, and *Picea* *abies*), 1 lycophyte (*Selaginella* *moellendorffii*) and 1 moss (*Physcomitrella* *patens*). We constructed a Maximum likelihood (ML) tree based on the amino acid sequences of the NAC domain of all the protein we identified. From this tree, we obtained all genes that clustered with the *VND*, *NST*, and *SMB* genes of *A. thaliana*. To explore the evolutionary history of the VNS gene family, the first and second codons were used to perform phylogenetic analysis. First, multiple codon alignment was constructed from the corresponding aligned protein sequences using PAL2NAL [45]. Second, using BioEdit, we deleted all positions with ambiguous alignments and only the positions of seven conserved subdomains of *VNS* genes remained. Finally, we used DAMBE5 [46] to obtain the alignment containing the first and second codon of the seven conserved subdomains of *VNS* genes. Based on the alignment, phylogenetic analysis was performed using the IQ-TREE software [10].

6.1.2 VNS genes in T. sinense and gene family evolution

Based on the conserved domains, we reconstructed the phylogenetic tree (Fig. S11a). As shown in the phylogenetic tree, genes homologous to *VND*, *NST*, and *SMB* each fell into different clades. In the *VND* group, two large clades were observed: one including *A.* *thaliana* *VND1*–*VND3* and *VND7*, and the other including *VND4*–*VND6*. Each of the two clades contains sequences from gymnosperms and angiosperms, suggesting that the duplication leading to the ancestors of *VND4*–*VND6*, and *VND1*–*VND3* and *VND7* occurred before the divergence of gymnosperms and angiosperms. In the clade containing *VND1*–*VND3* and *VND7*, genes homologous to *VND1*–*VND3* and *VND7* each formed a subclade and each subclade only contained sequences from angiosperms, suggesting that the duplication leading to the origin of the *VND7* ancestor occurred after the divergence of angiosperms and gymnosperms. This result is consistent with a previous study [47]. According to the phylogenetic tree, we can also infer the evolutionary history of the other two subfamilies of *VNS* genes. *NST* and *SMB* gene subfamilies also originated before the divergence of gymnosperms and angiosperms. After the divergence of some basal eudicots, the *NST* ancestor gene duplicated again, which led to the origin of ancestors of the *SND1* subfamily and the *NST1*–*NST2* subfamily. Of note, we found that in *T. sinense* there are 12 members of the *NST1*–*NST2* subfamily and there are 5 genes in a tandem cluster. *SMB* genes can be classified into two subfamilies, and ancestors of each subfamily originated before the divergence of gymnosperms and angiosperms.

6.2 *LBD* genes

The lateral organ boundaries (LBD) proteins define a family of plant-specific transcription factors and play a crucial role in the development of almost all plant organs, including the root, leaf, inflorescence, embryo, and male gametes [48, 49]. Two of the most studied LBDs are AtLBD6 (AS2) and AtLBD16. AtLBD6 represses cell proliferation in the adaxial domain of *A.* *thaliana* leaves and is critical for the development of a symmetrical, expanded lamina [50, 51], and AtLBD16 is involved in lateral root initiation together with the other related LBD proteins [52, 53]. Previous studies suggested that *ASL19/LBD30* and *ASL20/LBD18* are involved in the differentiation of tracheary elements in *A.* *thaliana.*

6.2.1 Identification and phylogenetic analysis of LBD genes

The LBD family (or AS2/LOB family) of proteins are characterized by the AS2 domain or the ‘‘lateral organ boundary domain’’ (LOB domain) in the N-terminal: this is hereafter designated as AS2/LOB domain. This domain is composed of a highly conserved cysteine rich C-motif (CX_2_CX_6_CX_3_C), an invariant glycine-containing GAS block, and a leucine zipper-like structure (LX_6_LX_3_LX_6_L) [54]. Although the N-terminal sequences of LBD proteins are highly conserved, the C-terminal regions are remarkably variable. For example, only eight groups of genes (total 18 genes), with two or three members each, and high degrees of identity among entire amino acid sequences [49]. Others genes or genes between each group did not exhibit significant similarity in terms of the deduced amino acid sequences of the C-terminal half [49].

Hence, we used two different approaches to identify gene sequences orthologous to *LBD18* and *LBD30* of *A. thaliana*. We first searched for all possible *LBD* genes with Pfam model PF03195, which corresponds to the LOB domain, using the hmmsearch command in the HMMER package [44]. This identify 976 sequences. Second, the C-terminal half sequences of *A. thaliana* *LBD18* and *LBD30* were used as queries for BLASTp searches with default parameters against the protein databases of the 919 sequences obtained by hmmsearch command [44]. Finally, the resulting hits were used for multiple sequence alignments using MAFFT [55]. The conserved regions from *AtLBD18* and *AtLBD30* homologous genes (*AtLBD18* and *AtLBD30* belong to one group of genes with high degrees of identity among the entire amino acid sequences), were subjected to phylogenetic analysis.

6.2.2 LBD genes in T. sinense and gene family evolution

In total, 976 *AS2/LBD* genes were identified from 22 selected plant species. All species from the moss to the angiosperms contained *LBD* genes. The results showed that AS2/LBD proteins belong to a relatively large gene family with copy numbers ranging from 26 in *L. chinense* and 78 in *M. acuminata*, to 108 in *P. abies*. *T. sinense* contains 60 *LBD* genes. Because of the short length of the AS2/LOB domain and the large number of sequences (a total of 976), it is difficult to evaluate the phylogenetic relationships among different groups of the AS2/LBD family based solely on sequences from the conserved AS2/LOB domains. Hence, the C-terminal half sequences of *AtLBD18*, and *AtLBD30* were used as queries to conduct BLASTp searches. Our study identified 27 genes belonging to the *LBD18/AtLBD30* subfamily in angiosperms and no genes belonging to the *LBD18/AtLBD30* subfamily in three gymnosperm species, one lycophyte species, and one moss species. This result suggests that the duplication leading to the origin of the *LBD18* subfamily may have occurred after the divergence of angiosperms and gymnosperms. These sequences were used to construct a phylogenetic tree (Fig. S5a). As shown in the ML tree of the *LBD18* subfamily (Supplementary Fig. S12a), the two genes (*LBD18* and *AtLBD30*) from *A.* *thaliana* formed one clade and two copies from *T. sinense* (Tesin01G0033700 and Tesin21G0059700) were orthologous to them.

6.3 Homologous genes to *A*. *thaliana* *Myb46* and *Myb83*

The MYB family is one of the largest transcription factor families in higher plants. The MYBs are further classified into four types: MYB-related, R2R3-MYB, 3R-MYB and 4R-MYB [56, 57] . R2R3-MYB proteins constitute the largest MYB subfamily. They play central roles in many aspects of plant biology, such as phenylpropanoid metabolism, determination of cell fate, secondary cell wall formation and responses to biotic and abiotic stresses [57, 58].

*A. thaliana* AtMYB46 and AtMYB83 are R2R3-MYB transcription factors. AtMYB46 is a key player in the transcriptional regulation of secondary cell wall biosynthesis [32]. AtMYB83 acts redundantly with AtMYB46 and these genes are capable of activating the entire biosynthetic program of secondary walls, and thus they are considered to be another level of master switch controlling secondary wall biosynthesis [33]. AtMYB46/83 functions in secondary cell wall deposition by activating biosynthetic pathways for cellulose, hemicellulose and lignin [32-34, 59]. The expression of *AtMYB46* and *AtMYB83* is directly regulated by the NAC transcription factor VND7 [32-34].

6.3.1 Identification and phylogenetic analysis of MYB genes

*A. thaliana* contains 126 *R2R3-MYB* genes [33]. Since there are a large number of *R2R3-MYB* genes in plant genomes, only R2R3-MYB proteins from *A. thaliana*, *O. sativa* and *T. sinense* were used in the phylogenetic analysis. R2R3-MYB proteins have a highly conserved N-terminal DNA-binding domain composed of two adjacent MYB repeats (the R2R3-MYB domain) and a variable C-terminal region [58, 60]. When we searched with Pfam model PF00249 using the hmmsearch command in HMMER, we found that the sequences including MYB-related, R2R3-MYB and 3R-MYB proteins were also obtained. Then we used *A. thaliana* R2R3-MYBs as queries to conduct BLASTp searches with e-value 1e-5 and obtained only R2R3-MYBs in *T. sinense*. Proteins were aligned using MAFFT [55] and the alignment was manually adjusted in BioEdit. The amino acids of conserved regions were used to construct a ML tree using IQ-TREE software [10].

6.3.2 MYB genes in T. sinense and gene family evolution

Totally, we identified 87 *R2R3-MYB* genes in the *T. sinense* genome. These sequences together with sequences from *A. thaliana* and *O. sativa* were subjected to phylogenetic analysis. As shown in the ML tree (Fig. S5b), sequences from the three species fell into different clades. We then used the nomenclature of Kranz et al. [61] revised by Stracke et al [56] and Solar et al. [62] to name the subgroups with a few modifications. Totally, R2R3-MYBs from *A. thaliana* and *O. sativa* and *T. sinense* can be classified into 31 subgroups (Supplementary Fig. S12b). Most subgroups except for 9 subgroups contain *T. sinense* sequences, suggesting these subgroups originated before the splitting of monocots and dicots. In subgroup MYB46 and MYB83, there are three *T. sinense* sequences (Tesin13G0114100, Tesin11G0137400, Tesin07G144300).

6.4 Cellulose synthase (CesA) family

A defining characteristic of the plant kingdom is the presence of a rigid cell wall. All plant cells are surrounded by a primary cell wall and some specific plant cells, such as tracheary elements, also form a specialized secondary cell wall. Cellulose is a major component of both primary and secondary cell walls. It is a polysaccharide consisting of glucose linked by β-1,4-glycosidic bonds, and is synthesized by the proteins encoded by the cellulose synthase (*CesA*) gene family [63]. *A.* *thaliana* contains 10 *CesA* genes: *CesAs4*, *7*, and *8* are involved in secondary cell wall biosynthesis [64] and the other *CesAs* are involved in synthesizing primary cell walls [65, 66]. *Ces4* and *Ces8* are regulated by MYB46 [67], which is a master switch for secondary wall formation in *A.* *thaliana* [32], and by VND7 [34], which is a master regulator of vessel element differentiation.

6.4.1 Identification and phylogenetic analysis of CesAs

Using the hmmsearch command in HMMER [44], the annotated proteome of *T. sinense* and other selected plants (the same species used in the *VNS* gene family analysis) was searched with two Pfam models, PF14569 and PF03552 [68], which correspond to the conserved domains localized at the N and C termini of CesA proteins, respectively. The BLASTp program was used to verify sequences, with *A.* *thaliana* CesA proteins serving as queries. Then, identical and defective sequences were identified and eliminated by manual inspection in BioEdit. Proteins were aligned using MAFFT [55] and the alignment was manually adjusted in BioEdit. Maximum likelihood trees were constructed using IQ-TREE software [10].

6.4.2 CesA genes in T. sinense and gene family evolution

In plants, CesAs are encoded by a small gene family. In the present study, 4–15 *CesA* genes were found in the land plant genomes. In the *T. sinense* genome, we identified 9 genes that encode proteins with significant sequence similarity to known *A. thaliana* *CesAs*. All *CesAs* identified in the present study were subjected to phylogenetic analysis. As shown in the ML tree (Fig. S12c), all *CesA* sequences from *P. patens* formed one clade. CesAs in seed plants fell into six major clades, which were designated as six subfamilies: *CesA1*/*10*, *CesA3*, *CesA6*, *CesA4*, *CesA7*, and *CesA8*. Each subfamily included sequences from angiosperms and gymnosperms, indicating that the ancient gene duplication events happened before the divergence of angiosperms and gymnosperms. In the phylogenetic tree, subfamilies *CesA4*, *CesA7*, and *CesA8* clustered together and subfamilies *CesA1*/*10*, *CesA3*, and *CesA6* also clustered together. This is consistent with their functional differentiation, in that the former is involved in secondary cell wall synthesis and the latter is involved in primary cell wall synthesis [64, 65]. *CesA* sequences of *S. moellendorffii* fell into each of these two clades. All subfamilies contain only one *A. thaliana* gene, except for subfamily *CesA1*/*10*, which contains two *A. thaliana* *CesA* genes (*CesA1* and *CesA10*), and subfamily *CesA6*, which contains four *A. thaliana* genes (*CesA2*, *CesA5*, *CesA6*, and *CesA9*). These two and four genes each formed one clade that did not contain *CesA* genes from other species, suggesting that they originated through lineage-specific duplication. The recent duplication of the *A. thaliana* *CesA6* family into *CesA2*, *5*, *6*, and *9* is consistent with the functional redundancy of these genes [66]. All subfamilies, including subfamilies *CesA4* and *CesA8*, also contained only one *T. sinense* gene except for subfamily *CesA1/10*, which contained three *T. sinense* genes. The three *T. sinense* genes clustered together and did not contain *CesA* genes from other species, also suggesting that they originated through lineage-specific duplications.

6.5 Papain-like cysteine protease (PLCP) gene family

The papain-like cysteine proteases (PLCPs), also known as C1A proteases, is a large class of proteolytic enzymes [69]. *A. thaliana* has 31 *PLCP* proteins*,* which can be divided into nine subfamilies: the CTB3-like subfamily, AALP-like subfamily, RD19A-like subfamily, SAG12-like subfamily, THI1-like subfamily, XBCP3-like subfamily, XCP2-like subfamily, CEP1-like subfamily, and RD21A-like subfamily [70]. PLCPs play crucial roles in plant growth and development, organ senescence, immunity, and stress responses [71-74]. Both members of the XCP subfamily of *A. thaliana* are tracheary element cysteine proteases [75], which are regulated by VND7 [34].

6.5.1 Identification and phylogenetic analysis of the PLCP gene family

PLCPs are encoded as preproproteins with a signal sequence that ensures that the proprotease enters the endomembrane system, an autoinhibitory prodomain that prevents premature activation of the protease, and a mature protease domain that contains the catalytic triad Cys, His, and Asn [70]. The pro-domain and the mature protease domain correspond to Pfam domains PF08246 (PF08127) and PF00112. We first searched the annotated proteome of all selected plants (as mentioned above) for potential *PLCP* genes using these two Pfam models. Redundant sequences and defective sequences were removed by manual inspection in BioEdit. The resulting amino acid sequences were aligned with MAFFT [55] and the conserved regions were used to construct the ML tree using IQ-TREE [10].

6.5.2 PLCP genes in T. sinense and gene family evolution

In total, 561 sequences were obtained from the 22 representative species from different major lineages of land plants. The copy number of *PLCP* genes varied from 16 to 39. In the moss *P. patens* and the lycophyte *S. moellendorffii*, 10 and 18 *PLCP* genes were found, respectively. By contrast, *T. sinense* has 37 *PLCP* genes. All sequences identified were aligned and used to construct the phylogenetic tree (Fig. 12d, e). Most *PLCP* genes fell into nine subfamilies (*SAG12*, *THI*, *CEP*, *XCP*, *RD21*, *XBCP3*, *RD19*, *ALP*, and *CTB*), which is in agreement with previous studies [70, 74] . In addition, two *T. sinense PLCP* genes together with several genes from other species did not fall into any subfamily; therefore, we assigned their clade to the group “unclassified L-like cathepsins”. According to their similarity to animal counterparts, *SAG12*, *THI*, *CEP*, *XCP*, *RD21*, and *XBCP3* have been classified as L-like cathepsins, and *RD19*, *ALP*, and *CTB* have been classified as F, H, and B-like cathepsins [70], respectively. In the phylogenetic tree, all subfamilies of L-like cathepsins were more related to each other, while subfamilies *RD19*, *ALP*, and *CTB* are relatively distant from them. L, F, H, and B-like proteins appeared in the moss and lycophyte, suggesting that ancient duplications led to the origin of these subfamilies. However, the moss and lycophyte did not contain genes from the *SAG12*, *THI*, *CEP* (L-like) subfamilies, suggesting that these families arose from relatively more recent duplications in vascular plants. As shown in the ML tree, *XCP* genes originated before the divergence of gymnosperms from *Selaginella moellendorffii.* Three genes in *T. sinense* *TsXCPa* (Tesin02G0006000), *TsXCPb* (Tesin06G0166500), and *TsXCPc* (Tesin24G0110100) are homologous to *A. thaliana* *XCP1* and *XCP2*.

**References**

1. Marcais G, Kingsford CA. fast, lock-free approach for efficient parallel counting of occurrences of k-mers. Bioinformatics 2011;27:764–70.
2. Liu B, Shi Y, Yuan Y, Hu X, Zhang H, Li N, et. al. Estimation of genomic characteristics by analyzing k-mer frequency in *de novo* genome projects. arXiv. 2012;arXiv:1308.2012v2.

3. Price AL, Jones NC, Pevzner PA. *De novo* identification of repeat families in large genomes. Bioinformatics. 2005;21:I351–I58.

4. Holt C, Yandell M. Maker2: An annotation pipeline and genome-database management tool for second-generation genome projects. BMC Bioinformatics. 2011;12:491.

5. Stanke M, Diekhans M, Baertsch R, Haussler D. Using native and syntenically mapped cdna alignments to improve *de novo* gene finding. Bioinformatics. 2008;24:637–44.

6. Kent WJ. BLAT–the BLAST-like alignment tool. Genome Res. 2002;12:656–64.

7. Jones P, Binns D, Chang H-Y, Fraser M, Li W, McAnulla C, et alHunter S. InterProScan 5: Genome-scale protein function classification. Bioinformatics. 2014;30:1236–40.

8. Yang ZH, Rannala B. Bayesian estimation of species divergence times under a molecular clock using multiple fossil calibrations with soft bounds. Mol Biol Evol. 2006;23:212–26.

9. Bouckaert R, Heled J, Kuehnert D, Vaughan T, Wu C-H, Xie D, et al. BEAST 2: A software platform for bayesian evolutionary analysis. PLoS Comput Biol. 2014;10:e1003537.

10. Lam-Tung N, Schmidt HA, von Haeseler A, Bui Quang M. IQ-TREE: A fast and effective stochastic algorithm for estimating maximum-likelihood phylogenies. Mol Biol Evol. 2015;32:268–74.

11. Li H-T, Yi T-S, Gao L-M, Ma P-F, Zhang T, Yang J-B, et al. Li D-Z: Origin of angiosperms and the puzzle of the jurassic gap. Nature Plants. 2019;5:461–70.

12. Kumar S, Stecher G, Suleski M, Hedges SB. Timetree: A resource for timelines, timetrees, and divergence times. Mol Biol Evol. 2017;34:1812–19.

13. Ming R, VanBuren R, Liu Y, Yang M, Han Y, Li L-T, et alShen-Miller J. Genome of the long-living sacred lotus (*Nelumbo nucifera* gaertn.). Genome Biol. 2013;14:R41.

14. Wang D, Zhang Y, Zhang Z, Zhu J, Yu J. KaKs_Calculator 2.0: A toolkit incorporating gamma-series methods and sliding window strategies. Genomics Proteomics Bioinformatics. 2010;8:77–80.

15. Cui L, Wall PK, Leebens-Mack JH, Lindsay BG, Soltis DE, Doyle JJ, et al. Widespread genome duplications throughout the history of flowering plants. Genome Res. 2006;16:738–49.

16. Li H. Aligning sequence reads, clone sequences and assembly contigs with BWA-MEM. arXiv. 2013;arXiv:1303.3997v2.

16. Tarasov A, Vilella AJ, Cuppen E, Nijman IJ, Prins P. Sambamba: Fast processing of NGS alignment formats. Bioinformatics. 2015;31:2032–34.

18. Garrison E, Marth G: Haplotype-based variant detection from short-read sequencing. arXiv. 2019;arXiv:1911.12285v1.

19. Chang CC, Chow CC, Tellier LCAM, Vattikuti S, Purcell SM, Lee JJ. Second-generation PLINK: Rising to the challenge of larger and richer datasets. Gigascience. 2015;4:7.

20. Alexander DH, Novembre J, Lange K. Fast model-based estimation of ancestry in unrelated individuals. Genome Res. 2009;19:1655–64.

21. Yang J, Lee SH, Goddard ME, Visscher PM. GCTA: A tool for genome-wide complex trait analysis. Am J Hum Genet. 2011;88:76–82.

22. Kumar S, Stecher G, Tamura K. Mega7: Molecular evolutionary genetics analysis version 7.0 for bigger datasets. Mol Biol Evol. 2016;33:1870–74.

23. Nei M, Li WH. Mathematical model for studying genetic variation in terms of restriction endonucleases. Proc Natl Acad Sci. 1979;76:5269–73.

24. Watterson GA. On the number of segregating sites in genetical models without recombination. Theor Popul Biol. 1975;7:256–76.

25. Korneliussen TS, Albrechtsen A, Nielsen R. ANGSD: Analysis of next generation sequencing data. BMC Bioinformatics. 2014;15:336.

26. Liu X, Fu Y-X. Exploring population size changes using SNP frequency spectra. Nat Genet. 2015;47:555–59.

27. Jensen MK, Kjaersgaard T, Nielsen MM, Galberg P, Petersen K, O'Shea C, Skriver K. The *Arabidopsis thaliana* NAC transcription factor family: Structure-function relationships and determinants of ANAC019 stress signalling. Biochem J. 2010;426:183–96.

28. Ohtani M, Nishikubo N, Xu B, Yamaguchi M, Mitsuda N, Goue N, et al. A NAC domain protein family contributing to the regulation of wood formation in poplar. Plant J. 2011;67:499–512.

29. Kubo M, Udagawa M, Nishikubo N, Horiguchi G, Yamaguchi M, Ito J, et al. Transcription switches for protoxylem and metaxylem vessel formation. Genes Dev. 2005;19:1855–60.

30. Yamaguchi M, Goue N, Igarashi H, Ohtani M, Nakano Y, Mortimer JC, et al. VASCULAR-RELATED NAC-domain6 and VASCULAR-RELATED NAC-domain7 effectively induce transdifferentiation into xylem vessel elements under control of an induction system. Plant Physiol. 2010;153:906–14.

31. Ohashi-Ito K, Oda Y, Fukuda H. *Arabidopsis* VASCULAR-RELATED NAC-domain6 directly regulates the genes that govern programmed cell death and secondary wall formation during xylem differentiation. Plant Cell. 2010;22:3461–73.

32. Zhong R, Richardson EA, Ye Z-H. The MYB46 transcription factor is a direct target of SND1 and regulates secondary wall biosynthesis in *Arabidopsis*. Plant Cell. 2007;19:2776–92.

33. McCarthy RL, Zhong R, Ye Z-H. MYB83 is a direct target of SND1 and acts redundantly with MYB46 in the regulation of secondary cell wall biosynthesis in *Arabidopsis*. Plant Cell Physiol. 2009;50:1950–64.

34. Yamaguchi M, Mitsuda N, Ohtani M, Ohme-Takagi M, Kato K, Demura T. VASCULAR-RELATED NAC-domain 7 directly regulates the expression of a broad range of genes for xylem vessel formation. Plant J. 2011;66:579–90.

35. Soyano T, Thitamadee S, Machida Y, Chua N-H. ASYMMETRIC LEAVES2-like19/LATERAL ORGAN BOUNDARIES domain30 and ASL20/LBD18 regulate tracheary element differentiation in *Arabidopsis*. Plant Cell. 2008;20:3359–73.

36. Zhong R, Demura T, Ye Z-H. SND1, a NAC domain transcription factor, is a key regulator of secondary wall synthesis in fibers of *Arabidopsis*. Plant Cell. 2006;18:3158–70.

37. Zhong R, Richardson EA, Ye Z-H. Two NAC domain transcription factors, SND1 and NST1, function redundantly in regulation of secondary wall synthesis in fibers of *Arabidopsis*. Planta. 2007;225:1603–11.

38. Mitsuda N, Seki M, Shinozaki K, Ohme-Takagi M. The NAC transcription factors NST1 and NST2 of *Arabidopsis* regulate secondary wall thickenings and are required for anther dehiscence. Plant Cell. 2005;17:2993–3006.

39. Mitsuda N, Iwase A, Yamamoto H, Yoshida M, Seki M, Shinozaki K, et al. NAC transcription factors, NST1 and NST3, are key regulators of the formation of secondary walls in woody tissues of *Arabidopsis*. Plant Cell. 2007;19:270–80.

40. Willemsen V, Bauch M, Bennett T, Campilho A, Wolkenfelt H, Xu J, et al. The NAC domain transcription factors FEZ and SOMBRERO control the orientation of cell division plane in *Arabidopsis* root stem cells. Dev Cell. 2008;15:913–22.

41. Bennett T, van den Toorn A, Sanchez-Perez GF, Campilho A, Willemsen V, Snel B, et al. SOMBRERO, BEARSKIN1, and BEARSKIN2 regulate root cap maturation in *Arabidopsis*. Plant Cell. 2010;22:640–54.

42. Olsen AN, Ernst HA, Lo Leggio L, Skriver K. NAC transcription factors: Structurally distinct, functionally diverse. Trends Plant Sci. 2005;10:79–87.

43. Ko J-H, Yang SH, Park AH, Lerouxel O, Han K-H. ANAC012, a member of the plant-specific NAC transcription factor family, negatively regulates xylary fiber development in *Arabidopsis* *thaliana*. Plant J. 2007;50:1035–48.

44. Finn RD, Clements J, Eddy SR. HMMER web server: Interactive sequence similarity searching. Nucleic Acids Res. 2011;39:W29–W37.

44. Suyama M, Torrents D, Bork P. PAL2NAL: Robust conversion of protein sequence alignments into the corresponding codon alignments. Nucleic Acids Res. 2006;34:W609–W612.

46. Xia X. DAMBE5: A comprehensive software package for data analysis in molecular biology and evolution. Mol Biol Evol. 2013;30:1720–28.

47. Wan T, Liu Z-M, Li L-F, Leitch AR, Leitch IJ, Lohaus R, et al. A genome for gnetophytes and early evolution of seed plants. Nature Plants. 2018;4:82–89.

48. Gombos M, Zombori Z, Szecsenyi M, Sandor G, Kovacs H, Gyorgyey J. Characterization of the LBD gene family in brachypodium: A phylogenetic and transcriptional study. Plant Cell Rep. 2017;36:61–79.

49. Matsumura Y, Iwakawa H, Machida Y, Machida C. Characterization of genes in the *ASYMMETRIC LEAVES2/LATERAL ORGAN BOUNDARIES* (*AS2*/*LOB*) family in *Arabidopsis thaliana*, and functional and molecular comparisons between AS2 and other family members. Plant J. 2009;58:525–37.

50. Guo M, Thomas J, Collins G, Timmermans MCP. Direct repression of KNOX loci by the ASYMMETRIC LEAVES1 complex of *Arabidopsis*. Plant Cell. 2008;20:48–58.

51. Iwakawa H, Iwasaki M, Kojima S, Ueno Y, Soma T, Tanaka H, et al. Expression of the *ASYMMETRIC LEAVES2* gene in the adaxial domain of *Arabidopsis* leaves represses cell proliferation in this domain and is critical for the development of properly expanded leaves. Plant J. 2007;51:173–84.

52. Goh T, Joi S, Mimura T, Fukaki H. The establishment of asymmetry in *Arabidopsis* lateral root founder cells is regulated by LBD16/ASL18 and related LBD/ASL proteins. Development. 2012;139:883–93.

53. Okushima Y, Fukaki H, Onoda M, Theologis A, Tasaka M. ARF7 and ARF19 regulate lateral root formation via direct activation of *LBD*/*ASL* genes in *Arabidopsis*. Plant Cell. 2007;19:118–30.

54. Shuai B, Reynaga-Pena CG, Springer PS. The lateral organ boundaries gene defines a novel, plant-specific gene family. Plant Physiol. 2002;129:747–61.

55. Katoh K, Misawa K, Kuma K-i, Miyata T. MAFFT: A novel method for rapid multiple sequence alignment based on fast fourier transform. Nucleic Acids Res. 2002;30:3059–66.

56. Stracke R, Werber M, Weisshaar B. The *R2R3-MYB* gene family in *Arabidopsis thaliana*. Curr Opin Plant Biol. 2001;4:447–56.

57. Dubos C, Stracke R, Grotewold E, Weisshaar B, Martin C, Lepiniec L. MYB transcription factors in *Arabidopsis*. Trends Plant Sci. 2010;15:573–81.

58. Jin H, Martin C. Multifunctionality and diversity within the plant *MYB*-gene family. Plant Mol Biol. 1999;41:577–85.

59. Ko J-H, Kim W-C, Han K-H. Ectopic expression of MYB46 identifies transcriptional regulatory genes involved in secondary wall biosynthesis in Arabidopsis. Plant J. 2009;60:649–65.

60. Rogers LA, Campbell MM. The genetic control of lignin deposition during plant growth and development. New Phytol. 2004;164:17–30.

61. Kranz HD, Denekamp M, Greco R, Jin H, Leyva A, Meissner RC, et al. Towards functional characterisation of the members of the *R2R3-MYB* gene family from *Arabidopsis thaliana*. Plant J. 1998;16:263–76.

62. Soler M, Camargo ELO, Carocha V, Cassan-Wang H, San Clemente H, Savelli B, et al. The eucalyptus grandis R2R3-MYB transcription factor family: Evidence for woody growth-related evolution and function. New Phytol. 2015;206:1364–77.

63. Kumar M, Turner S. Plant cellulose synthesis: Cesa proteins crossing kingdoms. Phytochemistry. 2015;112:91–99.

64. Taylor NG, Howells RM, Huttly AK, Vickers K, Turner SR. Interactions among three distinct CesA proteins essential for cellulose synthesis. Proc Natl Acad Sci. 2003;100:1450–55.

65. Somerville C. Cellulose synthesis in higher plants. Annu Rev Cell Dev Biol. 2006;22:53–78.

66. Carroll A, Specht CD. Understanding plant cellulose synthases through a comprehensive investigation of the cellulose synthase family sequences. Front in Plant Sci. 2011;2:5.

67. Kim W-C, Ko J-H, Kim J-Y, Kim J, Bae H-J, Han K-H. MYB46 directly regulates the gene expression of secondary wall-associated cellulose synthases in *Arabidopsis*. Plant J. 2013;73:26–36.

68. Zou X, Zhen Z, Ge Q, Fan S, Liu A, Gong W, et al. Genome-wide identification and analysis of the evolution and expression patterns of the cellulose synthase gene superfamily in *Gossypium* species. Gene. 2018;646:28–38.

69. Rawlings ND, Barrett AJ, Bateman A. MEROPS: The peptidase database. Nucleic Acids Res 2010;38:D227–D233.

70. Richau KH, Kaschani F, Verdoes M, Pansuriya TC, Niessen S, Stueber K, et al. Subclassification and biochemical analysis of plant papain-like cysteine proteases displays subfamily-specific characteristics. Plant Physiol. 2012;158:1583–99.

71. Misas-Villamil JC, van der Hoorn RAL, Doehlemann G. Papain-like cysteine proteases as hubs in plant immunity. New Phytol. 2016;212:902–07.

72. Diaz-Mendoza M, Velasco-Arroyo B, Gonzalez-Melendi P, Martinez M, Diaz I. C1A cysteine protease-cystatin interactions in leaf senescence. J Exp Bot. 2014;65:3825–33.

73. Lu H, Chandrasekar B, Oeljeklaus J, Misas-Villamil JC, Wang Z, Shindo T, et al. Subfamily-specific fluorescent probes for cysteine proteases display dynamic protease activities during seed germination. Plant Physiol. 2015;168:1462–75.

74. Zou Z, Xie G, Yang L. Papain-like cysteine protease encoding genes in rubber (*hevea brasiliensis*): Comparative genomics, phylogenetic, and transcriptional profiling analysis. Planta. 2017;246:999–1018.

75. Funk V, Kositsup B, Zhao C, Beers EP. The *Arabidopsis* xylem peptidase XCP1 is a tracheary element vacuolar protein that may be a papain ortholog. Plant Physiol. 2002;128:84–94.

**Supplementary Figures**

**
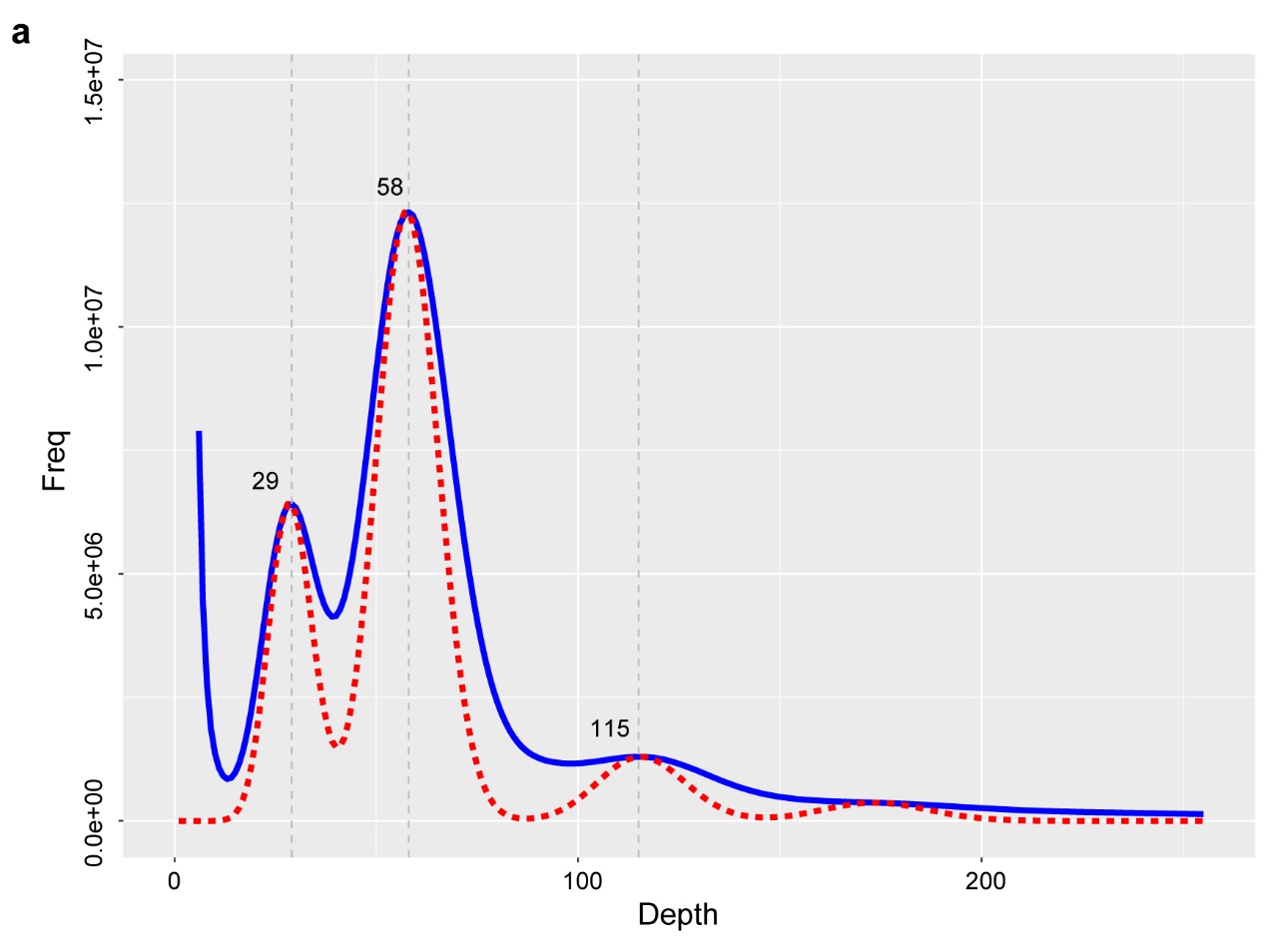
**

**Fig. S1 The 17-mer distribution of Illumina short reads in *T. sinense*.** The x-axis shows the frequency or the number of times a given k-mer (*k*-mer depth). The y-axis shows the total number of k-mers with a given frequency (a given depth). Two peaks (blue line) were observed (at 58x and 29x, respectively) indicating heterozygosity in *T. sinense*. Multiple peaks (such as 115x) indicate that many of the *k*-mers may come from recent genome duplications. The *k*-mer frequency follows a Poisson distribution in a given data set in theory (red dashed line).


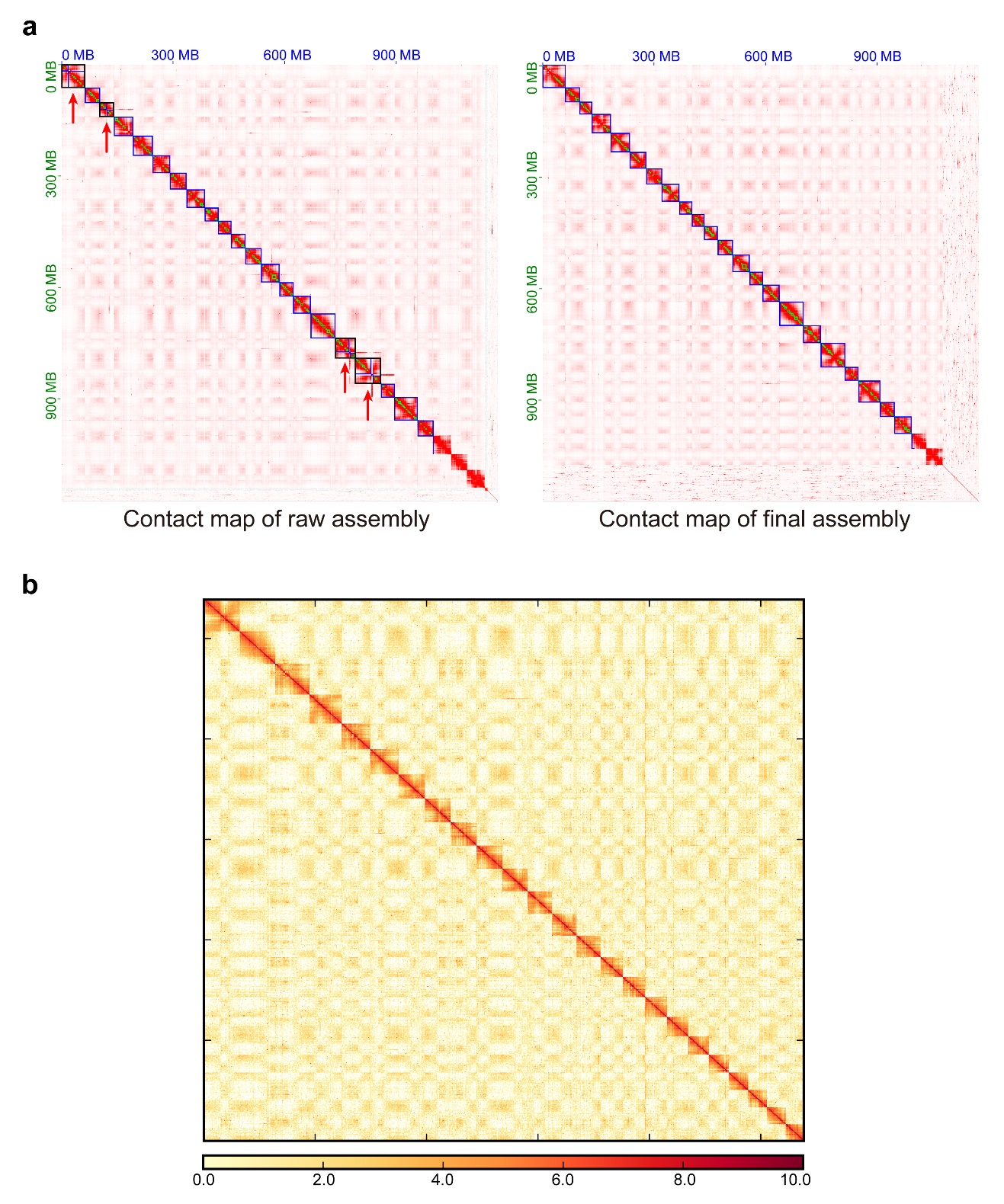


**Fig. S2 Hi-C Interaction heatmap of** ***T. sinense* reference genome.** a Hi-C Interaction heatmap of *T. sinense* scaffolds showing interactions before and after manual adjustments. The arrows indicates examples of misjoins before manual adjustments. b Hi-C interaction heatmap of *T. sinense* reference genome showing interactions between the 24 chromosomes (the chromosomes rank according to their chromosome number). The intensity of pixels represents the count of Hi-C links which indicate the likelihood of loci collocating in the nucleus. Darker red color indicates higher contact probability.


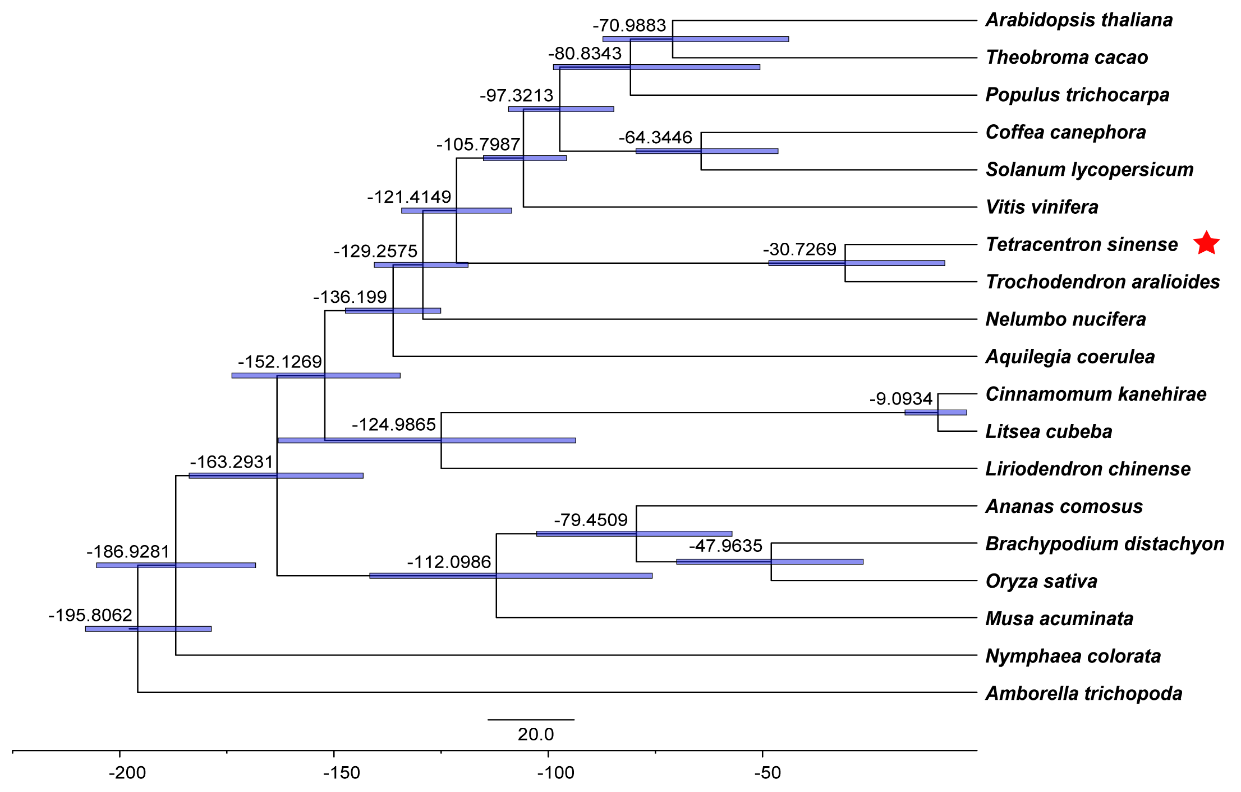


**Fig. S3 Time tree of 214 single-copy orthologs in nineteen species.** Time tree using the uncorrelated lognormal method in BEAST, numbers next to nodes indicate the median age, and blue bars correspond to the 95% highest posterior density (HPD).


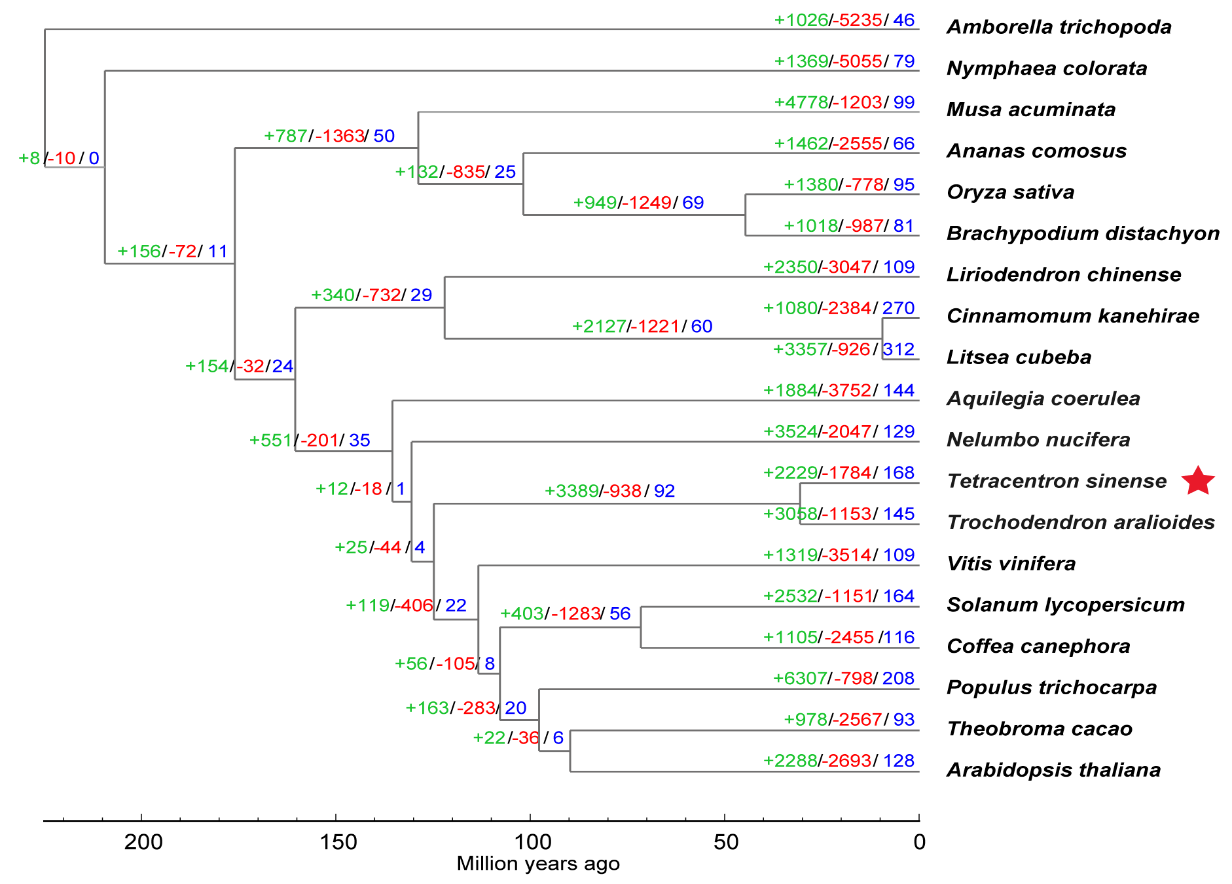


**Fig. S4 Estimation of gene family expansion and contraction on each evolutionary branch.** Green, red and blue numbers on branches indicate the number of expansion, contraction, and rapidly evolved gene families (family-wide p-value ≤ 0.01).


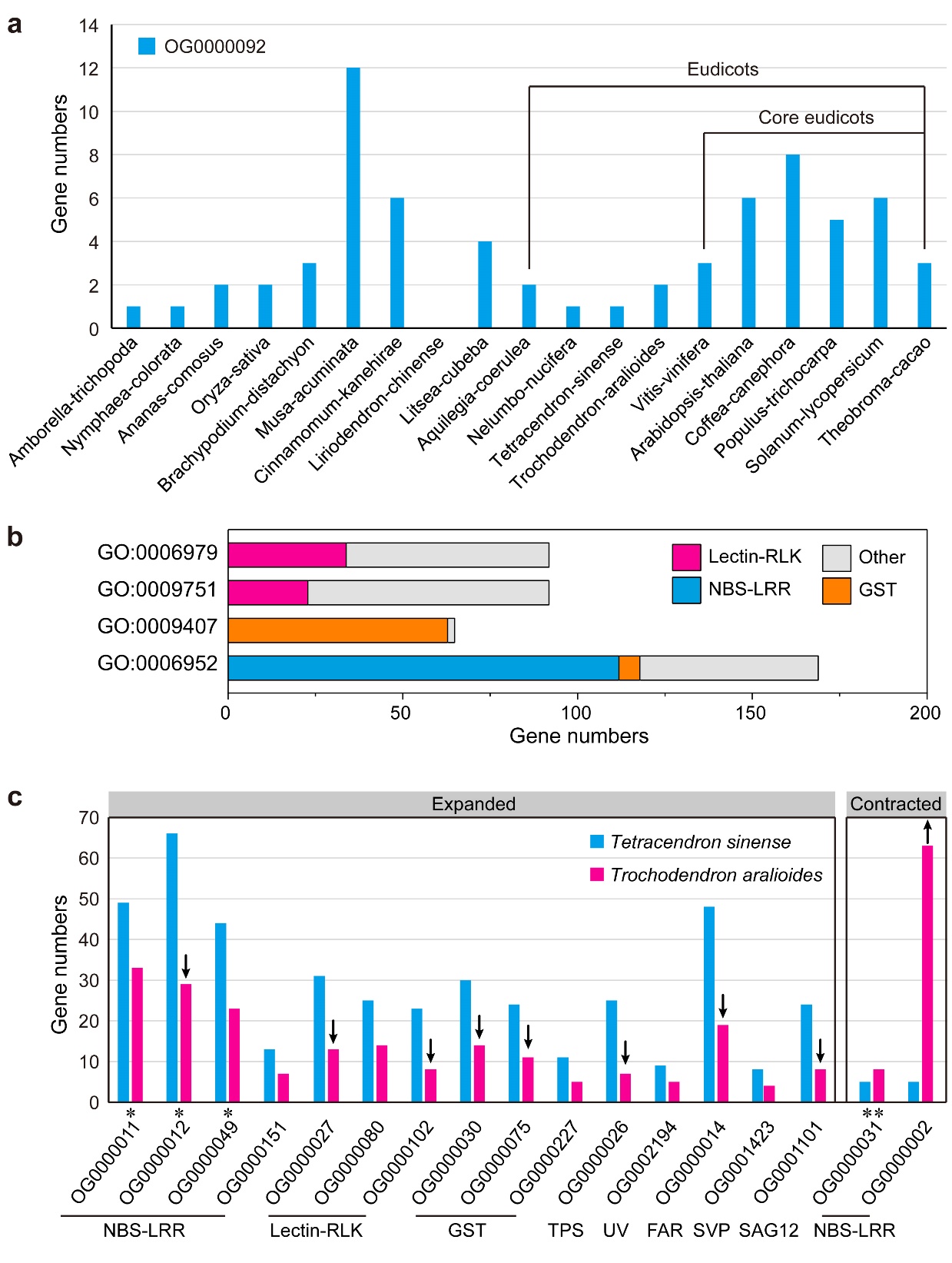


**Fig. S5 Gene family expansion and contraction. a** The copy number of TM3 gene family (OG0000092) in angiosperms. **b** The main composition of gene enriched in defense (OG: 0006952), toxin catabolic process (OG: 0009407), response to salicylic acid (OG: 0009751) and response to oxidative stress (OG: 0006979). **c** Gene family expansion and contraction in *T. sinense*. One single star indicates that the gene family also expanded in the lineage to common ancestor of Trochodendrales. Two stars indicates that the gene family have expanded in the lineages to eudicots, core eudicots and Trochodendrales. Downward arrow indicates that the gene families with expansion in *T. sinense* exhibiting contraction in *T. aralioides*, and upward arrow indicates the opposite pattern.


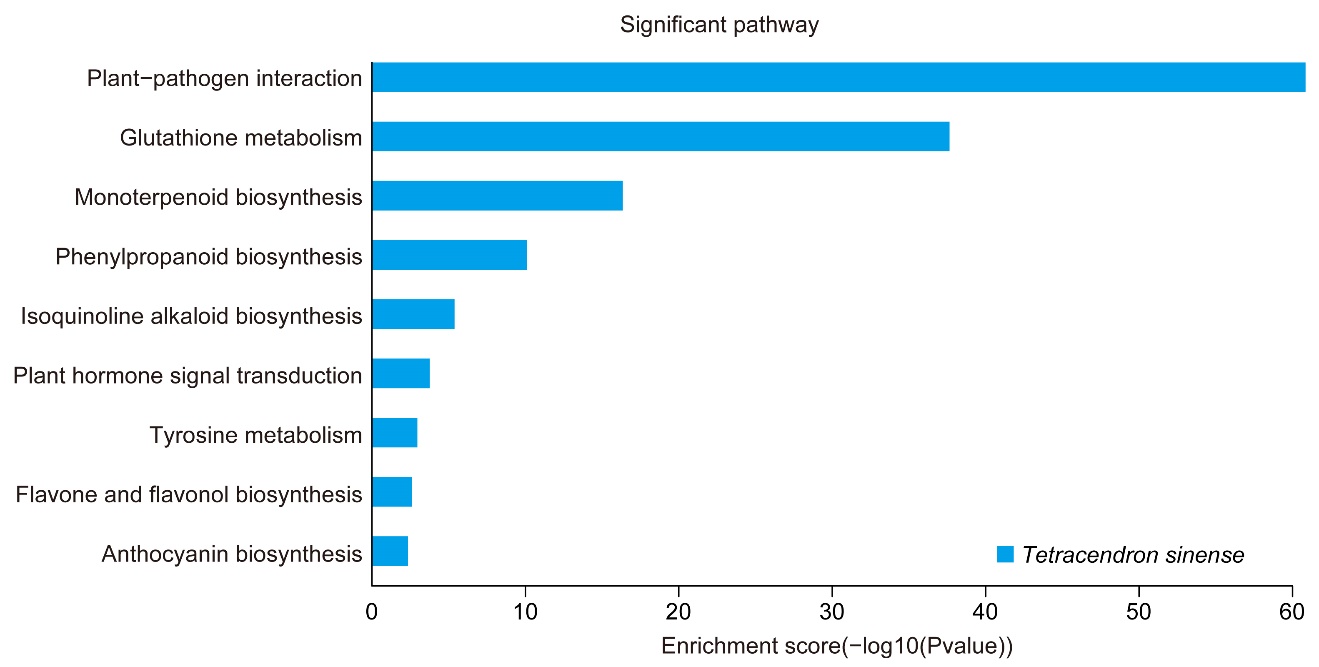


**Fig. S6 The enrichment of KEGG for the *T. sinense* rapidly expanded gene families.**


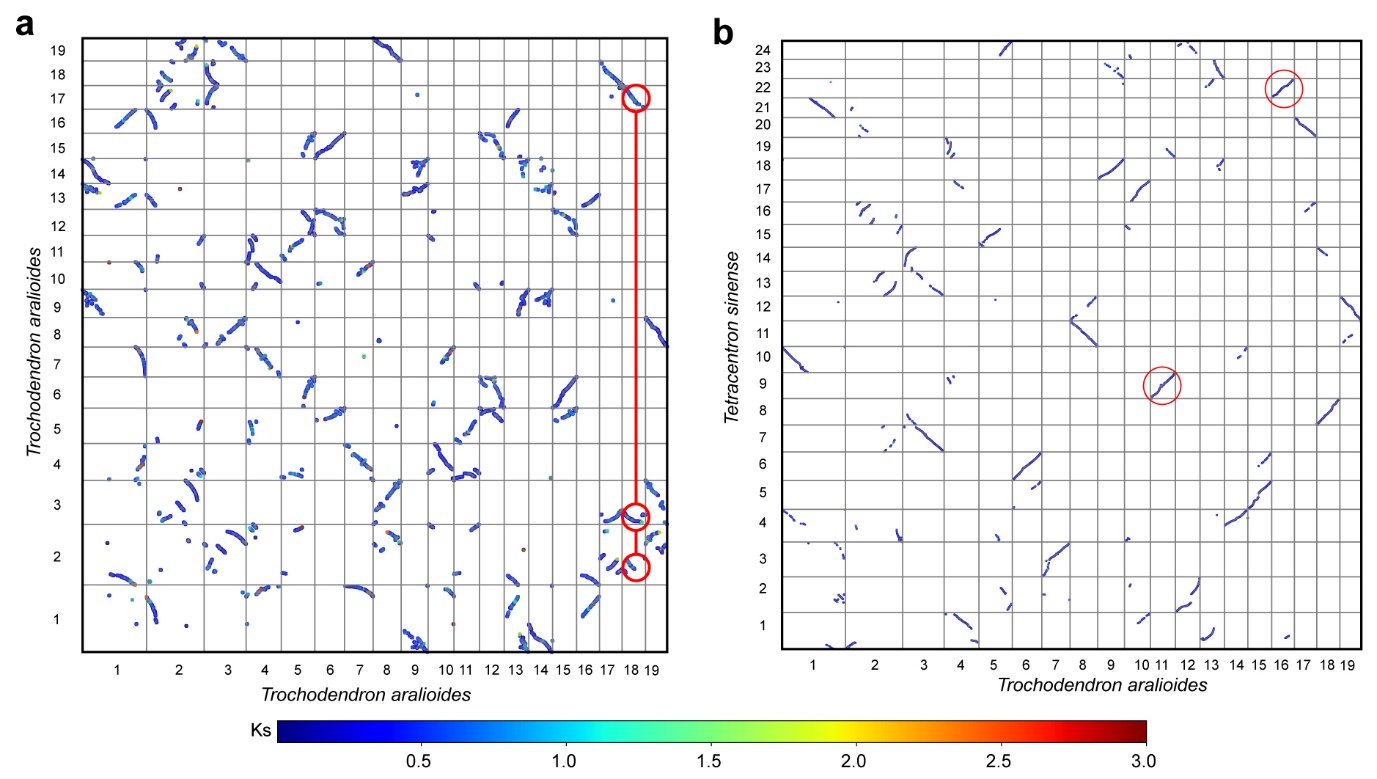


**Fig. S7 Synteny analysis within *T. aralioides* and between *T. aralioides* and *T. sinense*.** a, b Synteny analyses within *Trochodendron aralioides* genomes, and between *Tetracentron sinense* and *T. aralioides* genomes. Note the 1:3 syntenic depth relationship within the *T. aralioides* genome a, and the 1:1 syntenic depth relationship between *T. sinense* and *T. aralioides*


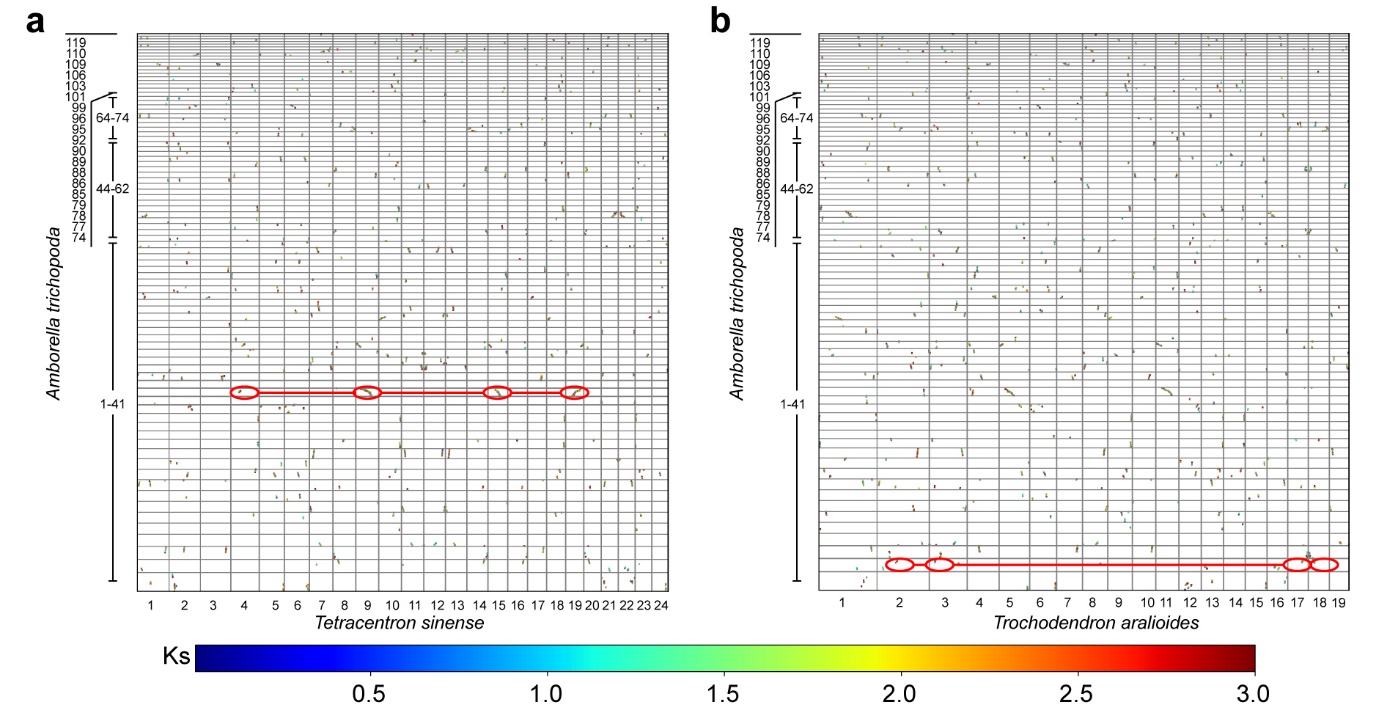


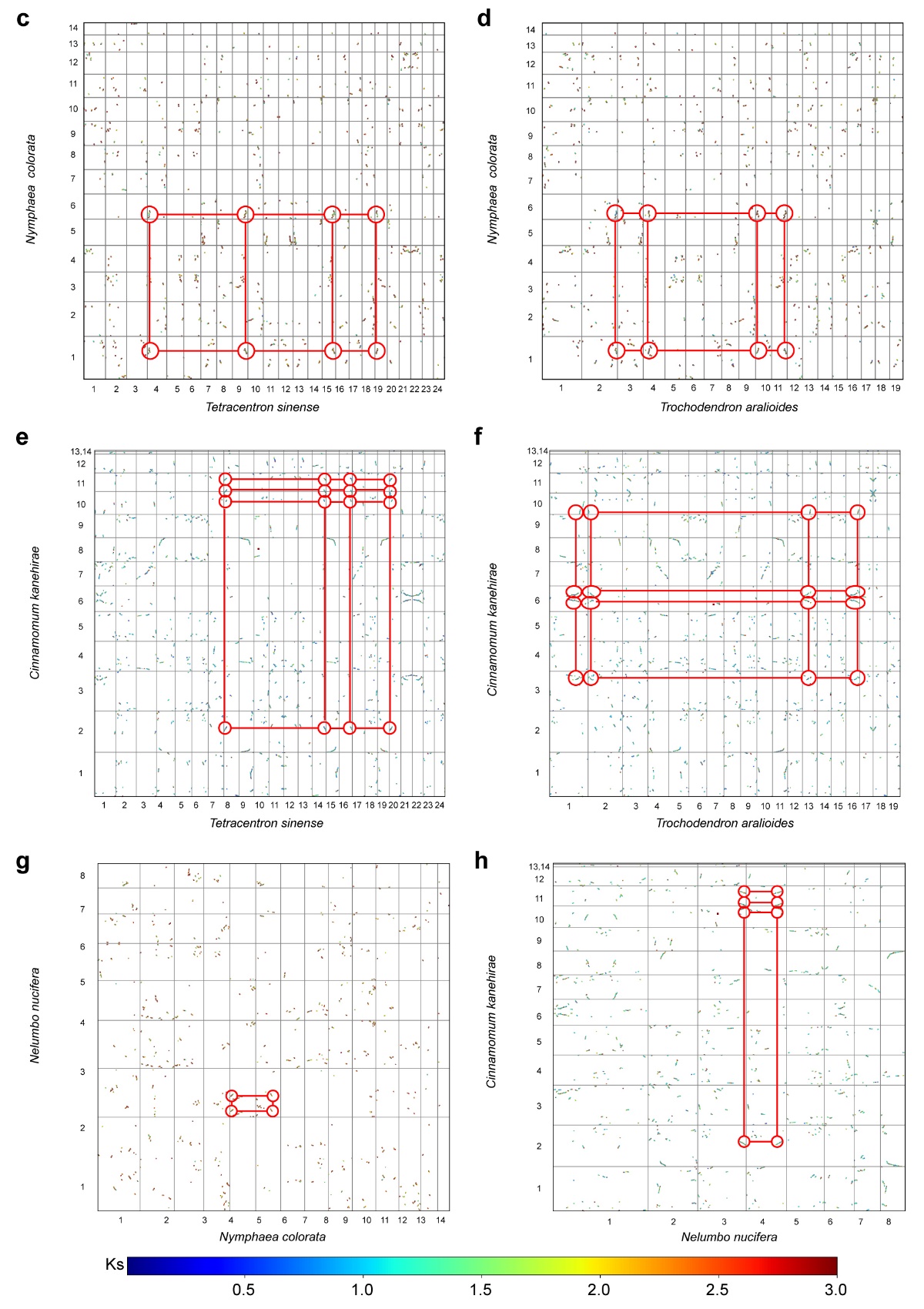


**Fig. S8 Synteny analysis *T. sinense*/*T. aralioides* and *A. trichopoda*, *N. colorata* and *C. kanehirae.* a, b** Synteny analyses between *A. trichopoda* and *T. sinense* and *T. aralioides* genomes, respectively. **c, d** Synteny analyses between *N. colorata* and *T. sinense* and *T. aralioides* genomes, respectively. **e, f** Synteny analyses between *C. kanehirae* and *T. sinense* and *T. aralioides* genomes, respectively. g**, h** Synteny analyses between *N.* *nucifera* and *N. colorata* and *C.* *kanehirae* genomes, respectively.


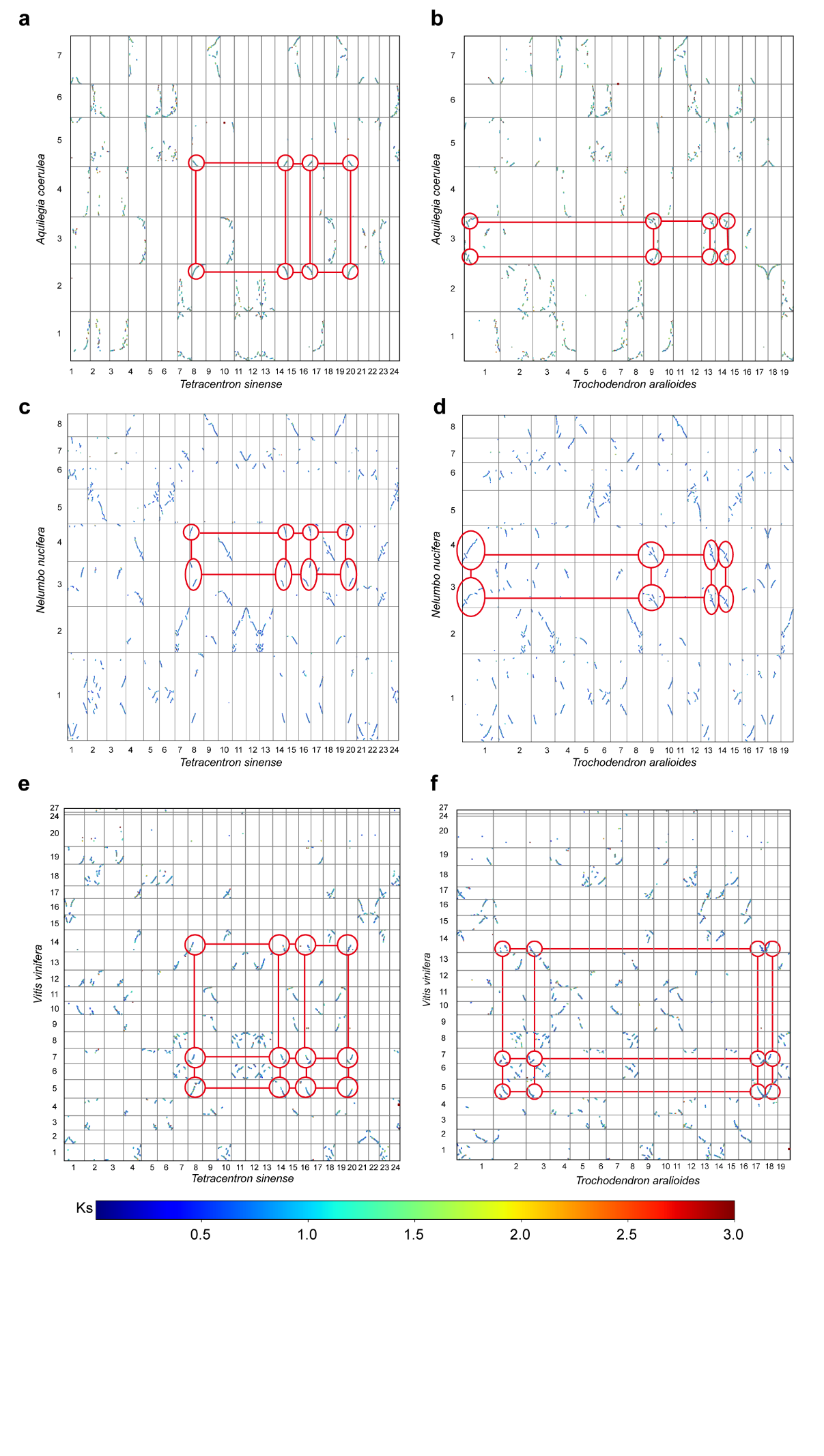


**Fig. S9 Synteny analyses** **between *T. sinense*/*T. aralioides* and *A. coerulea*, *N. nucifera* and *V. vinifera*.** **a**, **b** Synteny analyses and between *A.* *coerulea* and *T. sinense* and *T. aralioides* genomes, respectively. **c**, **d** Synteny analyses between the *N.* *nucifera* and *T. sinense* and *T. aralioides* genomes, respectively. **e, f** Synteny analyses between the *V. vinifera* and *T. sinense* and *T. aralioides* genomes, respectively.

*
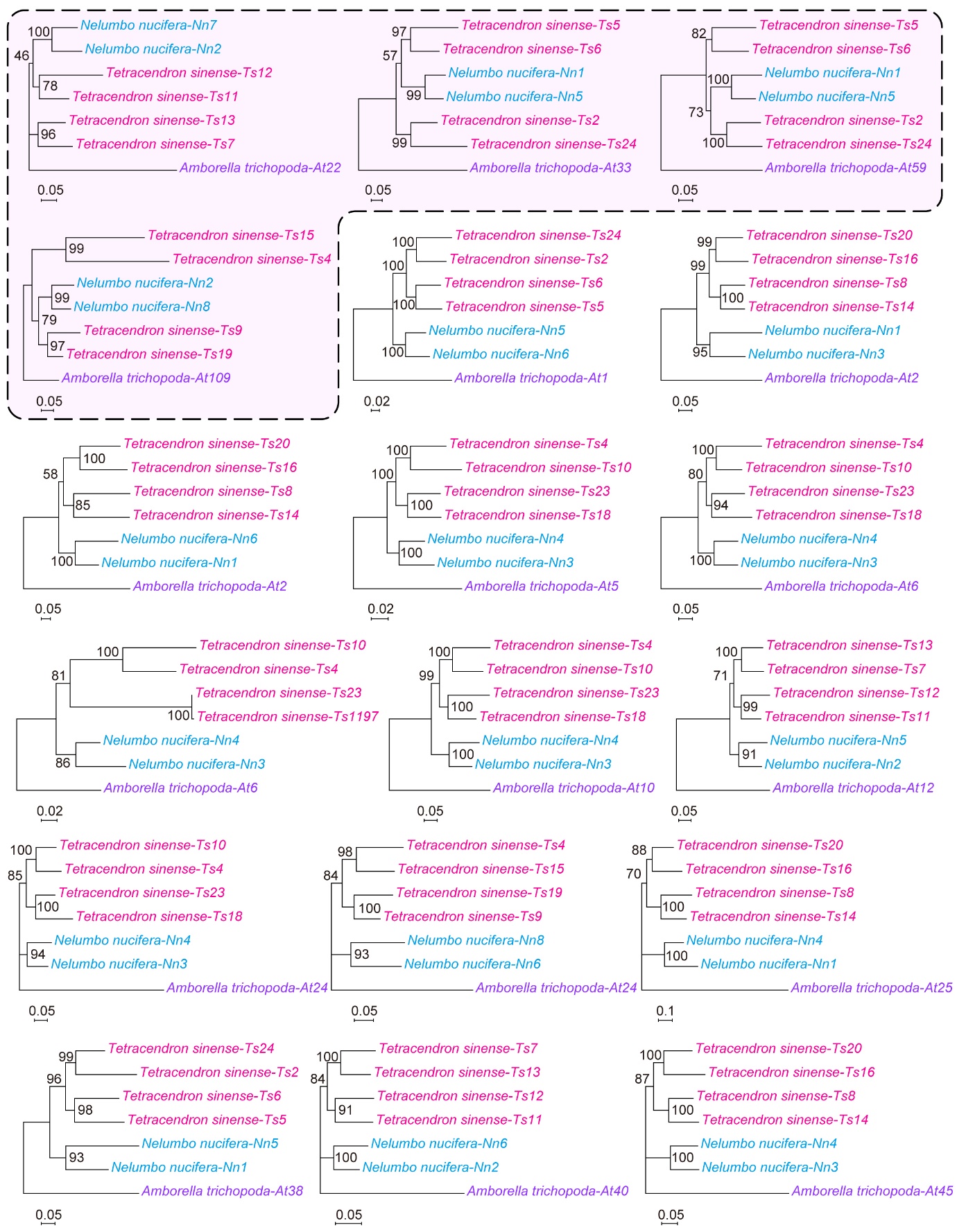
*


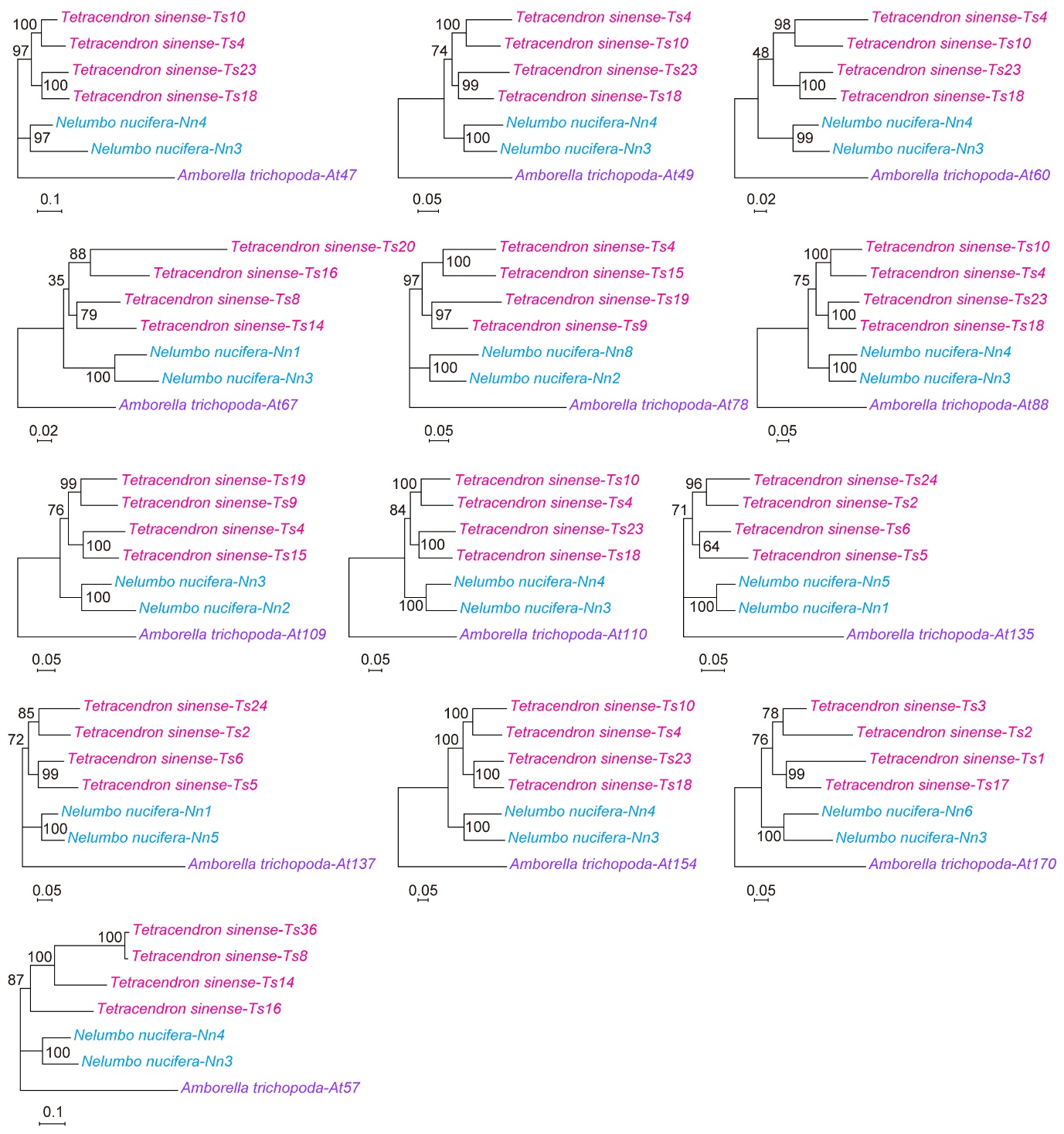


**Fig. S10 Phylogenomic analysis of anchor genes from the inter-genomic synteny blocks with ratio of 1:2:4 in *A. trichopoda*, *N. nucifera*, *and T. sinense*.** Each ML tree was constructed based on a concatenated data set. All trees except four (shadowed with pink) supported that the WGDs were lineage-specific in *N. nucifera*, *and T. sinense*.

**
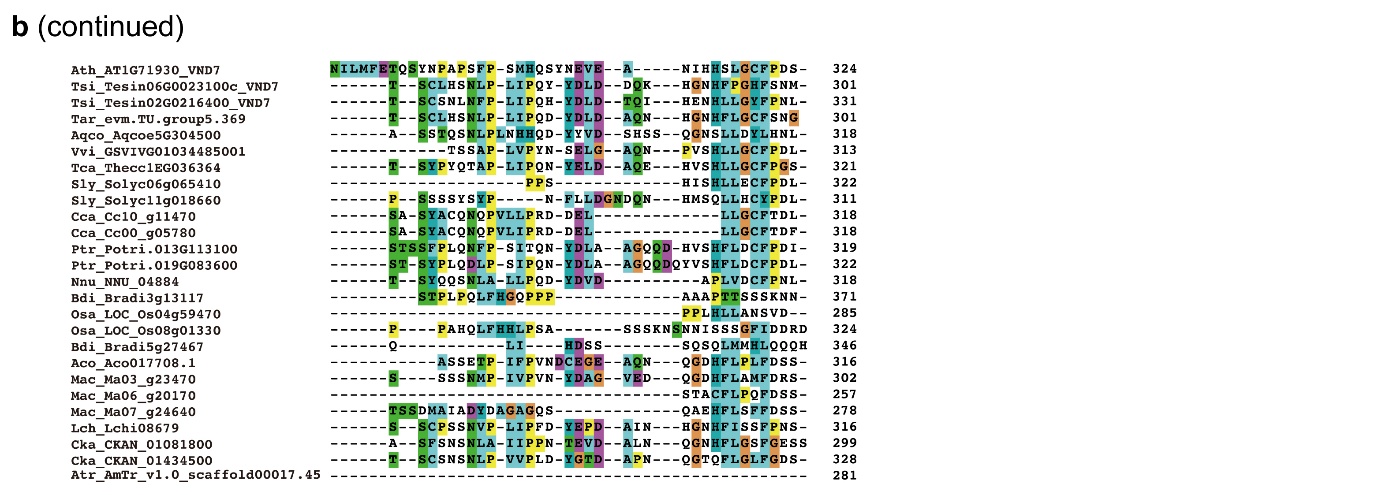
**

**Fig. S11. Phylogenetic analysis of *VNS* gene family and the amino-acid sequence alignment of VND7 proteins. a**, Maximum likelihood (ML) tree of genes from the *VNS* gene family. Bootstrap values (>50%) of the major clade are labeled next to the branches. **b**, Alignment of deduced amino-acid sequences of VND7. Alignments were calculated by MAFFT. Conserved NAC sub-domains are indicated by black lines. Red lines indicate conserved C-terminal sub-domains only occurring in VND proteins. (↓) denotes residues that are important for stabilizing the dimerization interface, and (#) denotes residues that are important for DNA binding. Species name abbreviations: Aco, *Ananas comosus*; Aqco, *Aquilegia* *coerulea*; Ath, *Arabidopsis* *thaliana*; Atr, *Amborella* *trichopoda*; Bdi, *Brachypodium* *distachyon*; Cca, *Coffea* *canephora*; Cka, *Cinnamomum* *kanehirae*; Lch, *Liriodendron* *chinense*; Mac, *Musa* *acuminate*; Nnu, *Nelumbo* *nucifera*; Osa, *Oryza* *sativa*; Ptr, *Populus* *trichocarpa*; Sly, *Solanum* *lycopersicum*; Tca, *Theobroma* *cacao*; Tsi, *Tetracentron* *sinense*; Tar, *Trochodendron aralioides*; Vvi, *Vitis* *vinifera*.

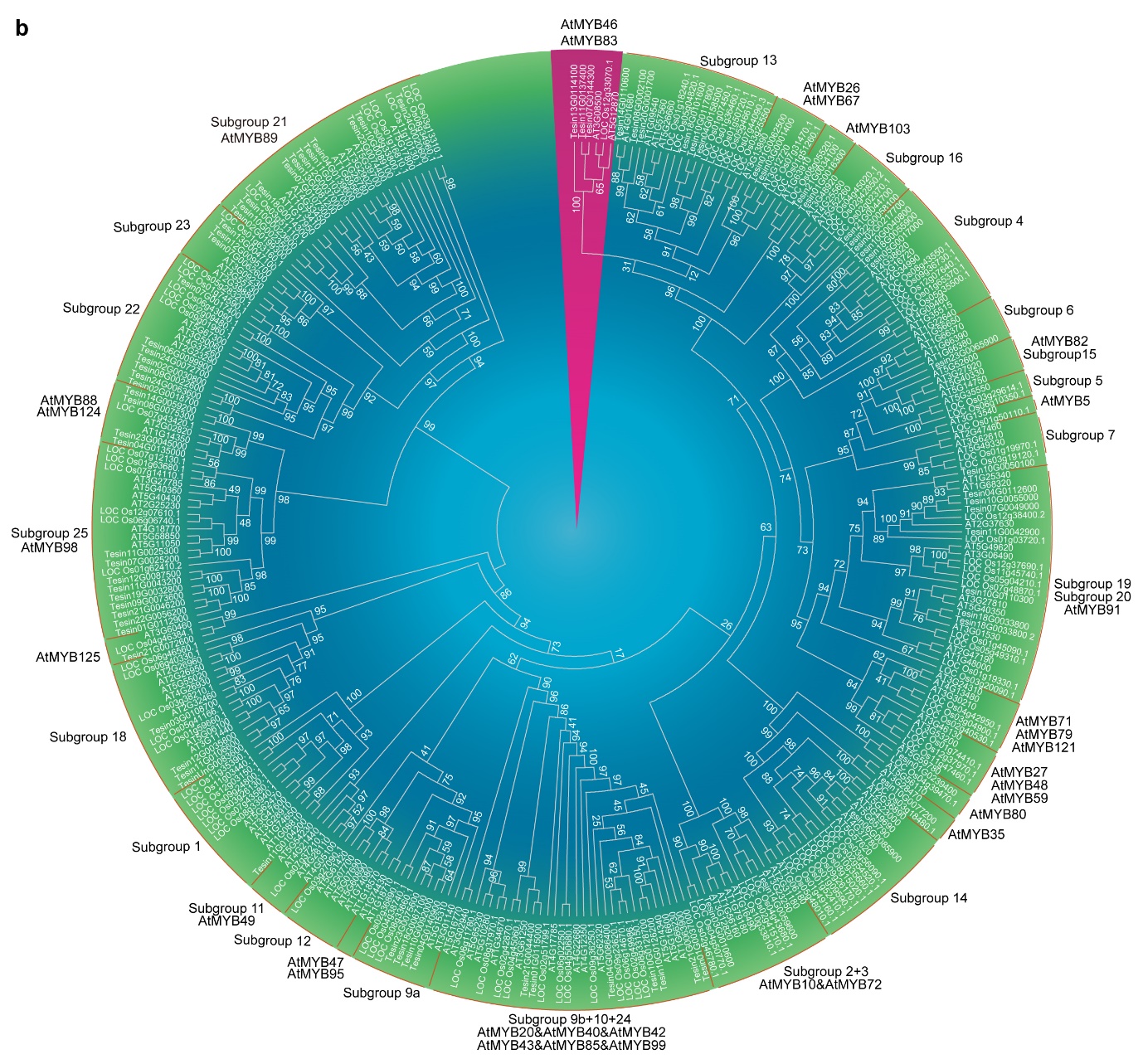

**Fig. S12 Maximum likelihood tree of genes from the *LBD*, *MYB*, *CesA* and PLCP gene families. a**, Maximum likelihood tree of genes from the *LBD* gene family. Numbers next to branches are bootstrap percentages (>50%) from Maximum Likelihood analysis. **b** ML tree of genes from the *MYB* gene family. The ML tree was based on the amino acids of conserved regions of the *MYB* gene family. Numbers next to branches are bootstrap percentages from Maximum Likelihood analysis. **c** ML tree of genes from the *CesA* gene family. The ML tree was based on the amino acids of conserved regions of the *CesA* gene family. Bootstrap values (>50%) of the major clade are labeled next to the branches. **d** The unrooted phylogenetic tree of 561 plant PLCPs is subdivided into nine PLCP subfamilies. Key bootstrap values are indicated. **e** Expanded phylogenetic tree of 561 plant PLCP proteases showing accession numbers. Numbers next to branches are bootstrap percentages (>50%) from Maximum Likelihood analysis. Species name abbreviations: Aco, *Ananas comosus*; Aqco, *Aquilegia* *coerulea*; Ath, *Arabidopsis* *thaliana*; Atr, *Amborella* *trichopoda*; Bdi, *Brachypodium* *distachyon*; Cca, *Coffea* *canephora*; Cka, *Cinnamomum* *kanehirae*; Lch, *Liriodendron* *chinense*; Mac, *Musa* *acuminate*; Nnu, *Nelumbo* *nucifera*; Osa, *Oryza* *sativa*; Ptr, *Populus* *trichocarpa*; Sly, *Solanum* *lycopersicum*; Tca, *Theobroma* *cacao*; Tsi, *Tetracentron* *sinense*; Tar, *Trochodendron aralioides*; Vvi, *Vitis* *vinifera*.


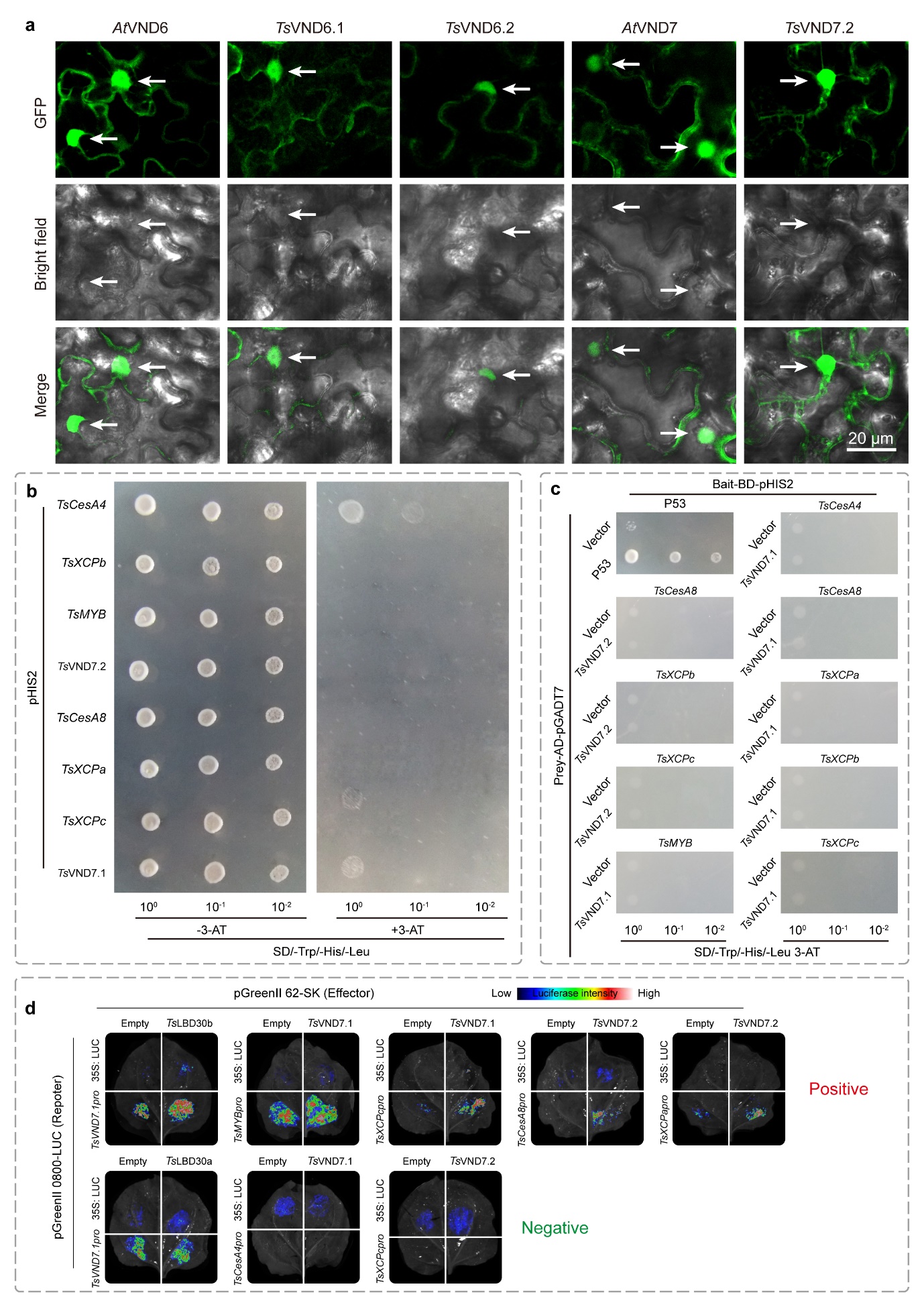


**Fig. S13 The cellular localization and function of *VND* genes. a** The VND genes product localize in the cell nucleus. Confocal images of *Nicotiana benthamiana* leaves transiently transformed with green fluorescent protein (GFP) fused to *At*VND6, *Ts*VND6.1, *Ts*VND6.2, *At*VND7, *Ts*VND7.2. The green fluorescence signal of the VND-GFP fusion proteins is accumulated in the cell nucleus. Scale bar represents 20 μm. **b**, **c** Yeast one-hybrid assay for interaction detection between transcription factor of *Ts*LBD30b, *Ts*VND7.1 or *Ts*VND7.2 and promoter of *TsVND7.2*, *TsMYB*, *TsCesA4*, *TsCesA8*, *TsXCPa*, *TsXCPb*, *or TsXCPc*. **b** *Ts*VND7.1, *Ts*VND7.2, *Ts*MYB, *Ts*CesA4, *Ts*CesA8, *Ts*XCPa, *Ts*XCPb, and *Ts*XCPc can grow without 3-amino-1,2,4-triazole (3-AT), but are inhibited by the application of the 3-AT. **c** Yeast one-hybrid assays showing that *Ts*VND7.1 does not interact with *Ts*MYB, *Ts*CesA4, *Ts*CesA8, *Ts*XCPa, *Ts*XCPb, or *Ts*XCPc, and that *Ts*VND7.2 cannot bind to the promoter of *TsCesA8*, *TsXCPb*, and *TsXCPc*. **d** Luciferase activity assay showing positive and negative interactions between transcription factors and promoters indicated in the figs.

**
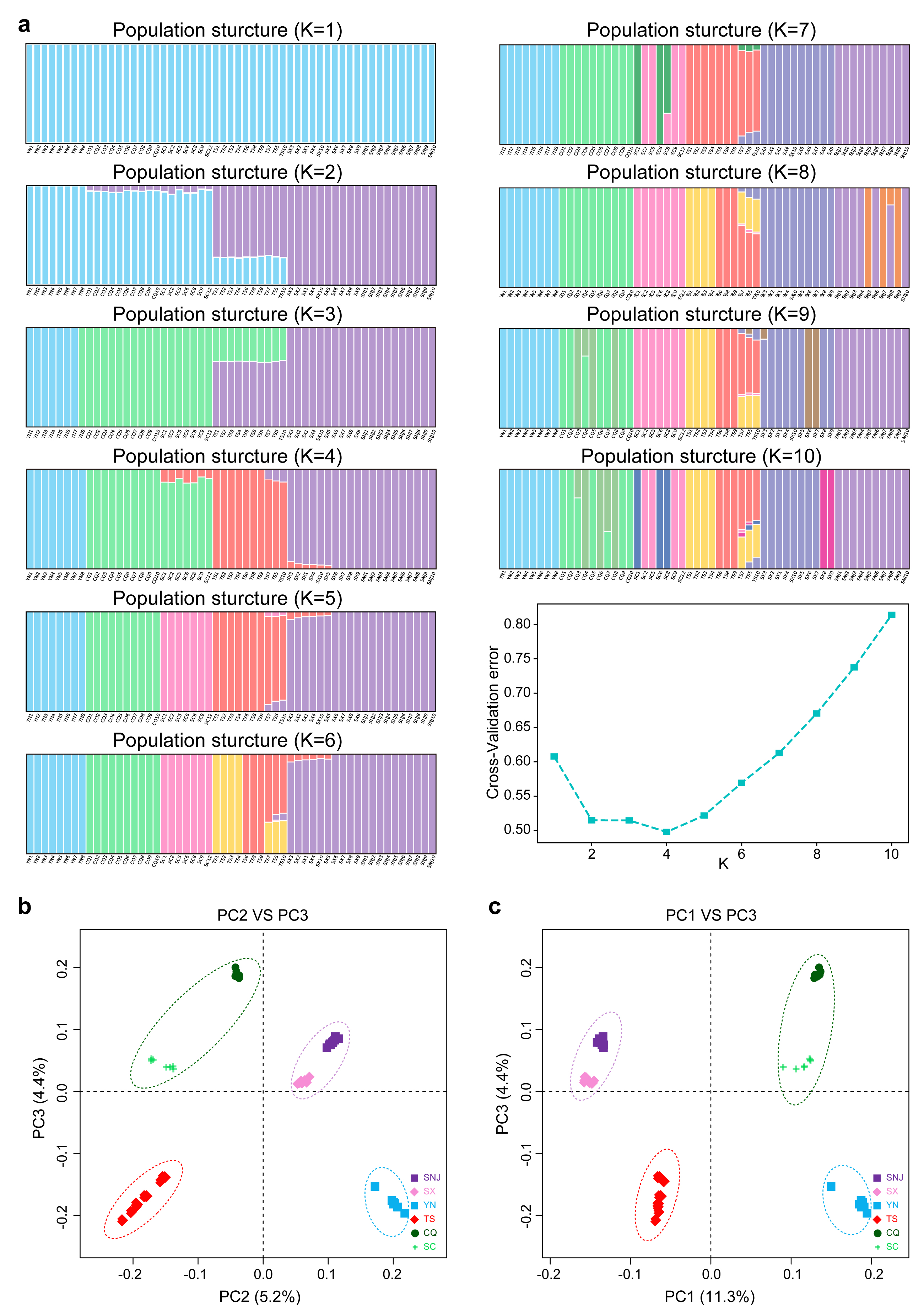
**

**Fig. S14 ADMIXTURE and principal component analysis of *Tetracentron sinense*. a**, ADMIXTURE results (K=1 to 10) and Cross validation (CV) error value for different model K. **b**, **c** Principal component analysis plot of 55 *Tetracentron sinense* samples from China. **b** the second and third principal components. **c** the first and third principal components.

**Supplementary Tables**

**Table S1: The statistics of repetitive sequences**

| **Repeat type** | **Length (bp)** | **Percentage of genome** |
| --- | --- | --- |
| Class I: Retrotransposons | **213,719,047** | 43.68% |
| LTR-Retrotransposons | 374,365,387 | 32.23% |
| LTR/Copia | 127,596,305 | 10.99% |
| LTR/Gypsy | 238,150,865 | 20.51% |
| LTR-other | 4,421,689 | 0.38% |
| Non-LTR Retrotransposons | 24,385,565 | 4.98% |
| LINE | 52,956,410 | 4.56% |
| SINE | 1,279,792 | 0.11% |
| Class II: DNA Transposons | **73,282,512** | 6.31% |
| CMC-EnSpm | 16,580,815 | 1.43% |
| hAT-Ac | 19,545,428 | 1.68% |
| hAT-Tag1 | 16,056,733 | 1.38% |
| DNA-other | 21,099,536 | 1.82% |
| Low Complexity | **1,728,293** | 0.15% |
| other | **19,861,869** | 1.71% |
| Unknown | **262,857,564** | 22.63% |
| Total content | **787,499,157** | 67.81% |

**Table S2: GO term enrichment of *T. sinense* rapid evolved gene families.**

| **GO ID** | **GO term** | **class** | **q_value** |
| --- | --- | --- | --- |
| GO:0043531 | ADP binding | MF | 2.34E-131 |
| GO:0031625 | ubiquitin protein ligase binding | MF | 1.72E-65 |
| GO:0004675 | transmembrane receptor protein serine/threonine kinase activity | MF | 1.87E-63 |
| GO:0007178 | transmembrane receptor protein serine/threonine kinase signaling pathway | BP | 1.87E-63 |
| GO:0001653 | peptide receptor activity | MF | 1.87E-63 |
| GO:0009755 | hormone-mediated signaling pathway | BP | 2.01E-61 |
| GO:0005524 | ATP binding | MF | 1.11E-50 |
| GO:0030247 | polysaccharide binding | MF | 3.25E-50 |
| GO:0045735 | nutrient reservoir activity | MF | 2.49E-49 |
| GO:0030145 | manganese ion binding | MF | 2.82E-49 |
| GO:0004674 | protein serine/threonine kinase activity | MF | 5.36E-48 |
| GO:0004364 | glutathione transferase activity | MF | 3.71E-46 |
| GO:0004672 | protein kinase activity | MF | 3.71E-46 |
| GO:0009506 | plasmodesma | CC | 1.94E-43 |
| GO:0046777 | protein autophosphorylation | BP | 1.65E-40 |
| GO:0009626 | plant-type hypersensitive response | BP | 3.30E-35 |
| GO:0043295 | glutathione binding | MF | 2.77E-29 |
| GO:0030246 | carbohydrate binding | MF | 8.11E-29 |
| GO:0016788 | hydrolase activity, acting on ester bonds | MF | 1.63E-28 |
| GO:0015074 | DNA integration | BP | 8.85E-27 |
| GO:0009407 | toxin catabolic process | BP | 1.42E-26 |
| GO:0009751 | response to salicylic acid | BP | 1.72E-25 |
| GO:0009636 | response to toxic substance | BP | 2.88E-25 |
| GO:0005576 | extracellular region | CC | 4.94E-25 |
| GO:0038023 | signaling receptor activity | MF | 1.15E-24 |
| GO:0006952 | defense response | BP | 1.24E-20 |
| GO:0008146 | sulfotransferase activity | MF | 4.10E-20 |
| GO:0009812 | flavonoid metabolic process | BP | 2.67E-19 |
| GO:0010018 | far-red light signaling pathway | BP | 5.08E-19 |
| GO:0009615 | response to virus | BP | 9.23E-19 |
| GO:0004601 | peroxidase activity | MF | 2.57E-18 |
| GO:0033383 | geranyl diphosphate metabolic process | BP | 4.84E-18 |
| GO:0043693 | monoterpene biosynthetic process | BP | 1.45E-17 |
| GO:0047036 | codeinone reductase (NADPH) activity | MF | 1.86E-17 |
| GO:0051762 | sesquiterpene biosynthetic process | BP | 4.78E-17 |
| GO:0046256 | 2,4,6-trinitrotoluene catabolic process | BP | 9.46E-17 |
| GO:0009820 | alkaloid metabolic process | BP | 1.55E-16 |
| GO:0010623 | developmental programmed cell death | BP | 1.64E-16 |
| GO:0050551 | myrcene synthase activity | MF | 2.09E-16 |
| GO:0016740 | transferase activity | MF | 2.69E-16 |
| GO:0033095 | aleurone grain | CC | 3.42E-16 |
| GO:0009311 | oligosaccharide metabolic process | BP | 4.22E-16 |
| GO:0005886 | plasma membrane | CC | 7.01E-16 |
| GO:0010334 | sesquiterpene synthase activity | MF | 1.17E-15 |
| GO:0048046 | apoplast | CC | 1.64E-15 |
| GO:0004867 | serine-type endopeptidase inhibitor activity | MF | 3.94E-15 |
| GO:0004462 | lactoylglutathione lyase activity | MF | 3.95E-15 |
| GO:0010282 | senescence-associated vacuole | CC | 4.65E-15 |
| GO:0042753 | positive regulation of circadian rhythm | BP | 1.86E-14 |
| GO:0020037 | heme binding | MF | 2.35E-14 |
| GO:0048527 | lateral root development | BP | 1.47E-13 |
| GO:0019897 | extrinsic component of plasma membrane | CC | 9.79E-13 |
| GO:0010333 | terpene synthase activity | MF | 2.93E-12 |
| GO:0060862 | negative regulation of floral organ abscission | BP | 5.45E-12 |
| GO:0009559 | embryo sac central cell differentiation | BP | 6.30E-12 |
| GO:0000325 | plant-type vacuole | CC | 8.10E-12 |
| GO:0042744 | hydrogen peroxide catabolic process | BP | 8.71E-12 |
| GO:0009816 | defense response to bacterium, incompatible interaction | BP | 1.24E-11 |
| GO:0005509 | calcium ion binding | MF | 1.24E-11 |
| GO:1901868 | ecgonine methyl ester catabolic process | BP | 1.44E-11 |
| GO:1901869 | ecgonine methyl ester biosynthetic process | BP | 1.44E-11 |
| GO:1901871 | ecgonone methyl ester catabolic process | BP | 1.44E-11 |
| GO:1901872 | ecgonone methyl ester biosynthetic process | BP | 1.44E-11 |
| GO:1990135 | flavonoid sulfotransferase activity | MF | 1.53E-11 |
| GO:1990169 | stress response to copper ion | BP | 1.53E-11 |
| GO:0006979 | response to oxidative stress | BP | 1.71E-11 |
| GO:0016682 | oxidoreductase activity, acting on diphenols and related substances as donors, oxygen as acceptor | MF | 2.82E-11 |
| GO:0010017 | red or far-red light signaling pathway | BP | 7.47E-11 |
| GO:0003690 | double-stranded DNA binding | MF | 9.90E-11 |
| GO:0048658 | anther wall tapetum development | BP | 1.25E-10 |
| GO:0007165 | signal transduction | BP | 1.49E-10 |
| GO:0002020 | protease binding | MF | 2.30E-10 |
| GO:0033834 | kaempferol 3-O-galactosyltransferase activity | MF | 2.48E-10 |
| GO:0004784 | superoxide dismutase activity | MF | 2.99E-10 |
| GO:0004714 | transmembrane receptor protein tyrosine kinase activity | MF | 3.47E-10 |
| GO:0080187 | floral organ senescence | BP | 4.04E-10 |
| GO:0008171 | O-methyltransferase activity | MF | 4.62E-10 |
| GO:0009734 | auxin-activated signaling pathway | BP | 6.30E-10 |
| GO:0051603 | proteolysis involved in cellular protein catabolic process | BP | 8.10E-10 |
| GO:0009639 | response to red or far red light | BP | 2.35E-09 |
| GO:0033303 | quercetin O-glucoside biosynthetic process | BP | 3.27E-09 |
| GO:0033330 | kaempferol O-glucoside biosynthetic process | BP | 3.27E-09 |
| GO:0003676 | nucleic acid binding | MF | 3.71E-09 |
| GO:0009266 | response to temperature stimulus | BP | 5.65E-09 |
| GO:0009617 | response to bacterium | BP | 9.19E-09 |
| GO:0003918 | DNA topoisomerase type II (ATP-hydrolyzing) activity | MF | 9.39E-09 |
| GO:0043078 | polar nucleus | CC | 9.91E-09 |
| GO:0033793 | aureusidin synthase activity | MF | 1.46E-08 |
| GO:0009992 | cellular water homeostasis | BP | 1.69E-08 |
| GO:0009055 | electron carrier activity | MF | 2.63E-08 |
| GO:0033485 | cyanidin 3-O-glucoside biosynthetic process | BP | 2.77E-08 |
| GO:0004197 | cysteine-type endopeptidase activity | MF | 3.43E-08 |
| GO:0090332 | stomatal closure | BP | 3.83E-08 |
| GO:0004803 | transposase activity | MF | 4.07E-08 |
| GO:0006313 | transposition, DNA-mediated | BP | 4.07E-08 |
| GO:0047052 | (S)-stylopine synthase activity | MF | 4.07E-08 |
| GO:0047053 | (S)-cheilanthifoline synthase activity | MF | 4.07E-08 |
| GO:0060416 | response to growth hormone | BP | 4.07E-08 |
| GO:0007568 | aging | BP | 5.51E-08 |
| GO:0004097 | catechol oxidase activity | MF | 6.17E-08 |
| GO:0005496 | steroid binding | MF | 8.52E-08 |
| GO:0006265 | DNA topological change | BP | 1.06E-07 |
| GO:0005764 | lysosome | CC | 1.13E-07 |
| GO:0009269 | response to desiccation | BP | 1.27E-07 |
| GO:0010497 | plasmodesmata-mediated intercellular transport | BP | 1.58E-07 |
| GO:0033075 | isoquinoline alkaloid biosynthetic process | BP | 3.15E-07 |
| GO:0047787 | delta4-3-oxosteroid 5beta-reductase activity | MF | 3.15E-07 |
| GO:0004383 | guanylate cyclase activity | MF | 3.96E-07 |
| GO:0006182 | cGMP biosynthetic process | BP | 3.96E-07 |
| GO:2000280 | regulation of root development | BP | 4.45E-07 |
| GO:0008506 | sucrose:proton symporter activity | MF | 4.94E-07 |
| GO:0015770 | sucrose transport | BP | 4.94E-07 |
| GO:0050630 | (iso)eugenol O-methyltransferase activity | MF | 5.24E-07 |
| GO:0008234 | cysteine-type peptidase activity | MF | 6.53E-07 |
| GO:0016045 | detection of bacterium | BP | 6.62E-07 |
| GO:0060867 | fruit abscission | BP | 8.77E-07 |
| GO:2000692 | negative regulation of seed maturation | BP | 8.77E-07 |
| GO:0005775 | vacuolar lumen | CC | 9.77E-07 |
| GO:0009729 | detection of brassinosteroid stimulus | BP | 1.10E-06 |
| GO:0048657 | anther wall tapetum cell differentiation | BP | 1.10E-06 |
| GO:0008202 | steroid metabolic process | BP | 1.28E-06 |
| GO:0010290 | chlorophyll catabolite transmembrane transporter activity | MF | 1.28E-06 |
| GO:0015431 | glutathione S-conjugate-exporting ATPase activity | MF | 1.28E-06 |
| GO:0016984 | ribulose-bisphosphate carboxylase activity | MF | 1.28E-06 |
| GO:0016114 | terpenoid biosynthetic process | BP | 1.33E-06 |
| GO:0046148 | pigment biosynthetic process | BP | 2.56E-06 |
| GO:0071456 | cellular response to hypoxia | BP | 2.90E-06 |
| GO:0080118 | brassinosteroid sulfotransferase activity | MF | 3.48E-06 |
| GO:0010187 | negative regulation of seed germination | BP | 4.42E-06 |
| GO:0040008 | regulation of growth | BP | 4.48E-06 |
| GO:1900140 | regulation of seedling development | BP | 4.67E-06 |
| GO:0004497 | monooxygenase activity | MF | 5.98E-06 |
| GO:0009821 | alkaloid biosynthetic process | BP | 6.95E-06 |
| GO:0009826 | unidimensional cell growth | BP | 7.18E-06 |
| GO:0051607 | defense response to virus | BP | 9.18E-06 |
| GO:0045487 | gibberellin catabolic process | BP | 1.05E-05 |
| GO:0033799 | myricetin 3'-O-methyltransferase activity | MF | 1.37E-05 |
| GO:0035671 | enone reductase activity | MF | 1.37E-05 |
| GO:0009555 | pollen development | BP | 1.55E-05 |
| GO:0009505 | plant-type cell wall | CC | 1.56E-05 |
| GO:0009295 | nucleoid | CC | 2.28E-05 |
| GO:0009742 | brassinosteroid mediated signaling pathway | BP | 2.37E-05 |
| GO:0008219 | cell death | BP | 2.37E-05 |
| GO:0060548 | negative regulation of cell death | BP | 2.37E-05 |
| GO:0009960 | endosperm development | BP | 2.44E-05 |
| GO:0016023 | cytoplasmic membrane-bounded vesicle | CC | 2.44E-05 |
| GO:0015749 | monosaccharide transport | BP | 3.49E-05 |
| GO:0010260 | organ senescence | BP | 3.49E-05 |
| GO:0033808 | 6'-deoxychalcone synthase activity | MF | 3.89E-05 |
| GO:0004715 | non-membrane spanning protein tyrosine kinase activity | MF | 4.89E-05 |
| GO:0030761 | 8-hydroxyquercitin 8-O-methyltransferase activity | MF | 8.19E-05 |
| GO:0030786 | (RS)-norcoclaurine 6-O-methyltransferase activity | MF | 8.19E-05 |
| GO:0033800 | isoflavone 7-O-methyltransferase activity | MF | 8.19E-05 |
| GO:0034002 | (R)-limonene synthase activity | MF | 8.19E-05 |
| GO:0034340 | response to type I interferon | BP | 8.19E-05 |
| GO:0034342 | response to type III interferon | BP | 8.19E-05 |
| GO:0047364 | desulfoglucosinolate sulfotransferase activity | MF | 8.19E-05 |
| GO:0047366 | quercetin-3-sulfate 4'-sulfotransferase activity | MF | 8.19E-05 |
| GO:0050552 | (4S)-limonene synthase activity | MF | 8.19E-05 |
| GO:0009687 | abscisic acid metabolic process | BP | 9.58E-05 |
| GO:0010218 | response to far red light | BP | 0.000100374 |
| GO:0016772 | transferase activity, transferring phosphorus-containing groups | MF | 0.000111992 |
| GO:0005840 | ribosome | CC | 0.000116701 |
| GO:0015145 | monosaccharide transmembrane transporter activity | MF | 0.000133242 |
| GO:0080148 | negative regulation of response to water deprivation | BP | 0.000137793 |
| GO:0048577 | negative regulation of short-day photoperiodism, flowering | BP | 0.000265601 |
| GO:0009910 | negative regulation of flower development | BP | 0.000286423 |
| GO:0032440 | 2-alkenal reductase [NAD(P)] activity | MF | 0.000361652 |
| GO:0016705 | oxidoreductase activity, acting on paired donors, with incorporation or reduction of molecular oxygen | MF | 0.000409562 |
| GO:0008270 | zinc ion binding | MF | 0.000463633 |
| GO:0010212 | response to ionizing radiation | BP | 0.000646036 |
| GO:0090558 | plant epidermis development | BP | 0.000646036 |
| GO:0042803 | protein homodimerization activity | MF | 0.000652246 |
| GO:0004709 | MAP kinase activity | MF | 0.000927729 |
| GO:0000900 | translation repressor activity, nucleic acid binding | MF | 0.001365594 |
| GO:0005385 | zinc ion transmembrane transporter activity | MF | 0.001365594 |
| GO:0010214 | seed coat development | BP | 0.001608979 |
| GO:0006749 | glutathione metabolic process | BP | 0.001638912 |
| GO:0050474 | (S)-norcoclaurine synthase activity | MF | 0.001666081 |
| GO:1901708 | (+)-3'-hydroxylarreatricin biosynthetic process | BP | 0.001666081 |
| GO:1901709 | (+)-larreatricin metabolic process | BP | 0.001666081 |
| GO:0005788 | endoplasmic reticulum lumen | CC | 0.001787093 |
| GO:0010268 | brassinosteroid homeostasis | BP | 0.001897327 |
| GO:0050593 | N-methylcoclaurine 3'-monooxygenase activity | MF | 0.002135052 |
| GO:0008970 | phosphatidylcholine 1-acylhydrolase activity | MF | 0.002845348 |
| GO:0009410 | response to xenobiotic stimulus | BP | 0.003087885 |
| GO:0010262 | somatic embryogenesis | BP | 0.003087885 |
| GO:0045071 | negative regulation of viral genome replication | BP | 0.003262223 |
| GO:0047763 | caffeate O-methyltransferase activity | MF | 0.003327154 |
| GO:0005506 | iron ion binding | MF | 0.003922374 |
| GO:0050162 | oxalate oxidase activity | MF | 0.004779847 |
| GO:0071215 | cellular response to abscisic acid stimulus | BP | 0.008291704 |
| GO:0009650 | UV protection | BP | 0.008463788 |
| GO:0031349 | positive regulation of defense response | BP | 0.008463788 |
| GO:0008080 | N-acetyltransferase activity | MF | 0.008587914 |
| GO:0009808 | lignin metabolic process | BP | 0.010549128 |
| GO:0071446 | cellular response to salicylic acid stimulus | BP | 0.013087668 |
| GO:0008298 | intracellular mRNA localization | BP | 0.013087668 |
| GO:0016592 | mediator complex | CC | 0.013188714 |
| GO:0005355 | glucose transmembrane transporter activity | MF | 0.013246889 |
| GO:0035428 | hexose transmembrane transport | BP | 0.013246889 |
| GO:0046323 | glucose import | BP | 0.013246889 |
| GO:0047056 | (S)-canadine synthase activity | MF | 0.014014801 |
| GO:0080059 | flavonol 3-O-arabinosyltransferase activity | MF | 0.014014801 |
| GO:0005618 | cell wall | CC | 0.016736618 |
| GO:0016301 | kinase activity | MF | 0.017278152 |
| GO:0022891 | substrate-specific transmembrane transporter activity | MF | 0.017378215 |
| GO:0010942 | positive regulation of cell death | BP | 0.018624597 |
| GO:0047714 | galactolipase activity | MF | 0.018624597 |
| GO:0071365 | cellular response to auxin stimulus | BP | 0.018769221 |
| GO:0016628 | oxidoreductase activity, acting on the CH-CH group of donors, NAD or NADP as acceptor | MF | 0.019270716 |
| GO:0051091 | positive regulation of sequence-specific DNA binding transcription factor activity | BP | 0.019270716 |
| GO:0000166 | nucleotide binding | MF | 0.019294468 |
| GO:0043024 | ribosomal small subunit binding | MF | 0.019901261 |
| GO:0019199 | transmembrane receptor protein kinase activity | MF | 0.020403187 |
| GO:2000815 | regulation of mRNA stability involved in response to oxidative stress | BP | 0.026489161 |
| GO:0009704 | de-etiolation | BP | 0.028004526 |
| GO:0000956 | nuclear-transcribed mRNA catabolic process | BP | 0.032476928 |
| GO:0009834 | plant-type secondary cell wall biogenesis | BP | 0.033107889 |
| GO:0016099 | monoterpenoid biosynthetic process | BP | 0.033441635 |
| GO:0005615 | extracellular space | CC | 0.033462731 |
| GO:0009620 | response to fungus | BP | 0.03440185 |
| GO:0048438 | floral whorl development | BP | 0.034572601 |
| GO:0005623 | cell | CC | 0.043861137 |
| GO:0031012 | extracellular matrix | CC | 0.044119383 |
| GO:0030795 | jasmonate O-methyltransferase activity | MF | 0.04661717 |
| GO:2000030 | regulation of response to red or far red light | BP | 0.04661717 |
| GO:0044530 | supraspliceosomal complex | CC | 0.047410708 |
| GO:0034009 | isoprene synthase activity | MF | 0.047410708 |
| GO:0009543 | chloroplast thylakoid lumen | CC | 0.049947507 |

**Table S3: Sample size and genetic diversity of different populations of *T. sinense***

| **Populations** | **Trees** | **Region** | **π (10^−3^)** | **θ_w_ (10^-3^)** |
| --- | --- | --- | --- | --- |
| YN | 8 | Southwestern China | 6.48 | 6.30 |
| CQ | 10 | Central China | 10.79 | 12.53 |
| SC | 7 | Central China | 10.11 | 10.14 |
| TS | 10 | Central China | 10.77 | 10.74 |
| SNJ | 10 | Central China | 11.25 | 13.22 |
| SX | 10 | Central China | 10.79 | 11.38 |
| Total | 55 | China | 12.8 | 22.4 |

π: average number of pairwise nucleotide differences per site. θ_W_: Watterson’s estimator of θ per base pair.

**Table S4: Matrix of pairwise *F*st value of six *T. sinense* populations**

|  | **CQ** | **SC** | **TS** | **SX** | **SNJ** |
| --- | --- | --- | --- | --- | --- |
| **SC** | 0.195 |  |  |  |  |
| **TS** | 0.267 | 0.257 |  |  |  |
| **SX** | 0.292 | 0.316 | 0.181 |  |  |
| **SNJ** | 0.273 | 0.306 | 0.201 | 0.102 |  |
| **YN** | 0.293 | 0.336 | 0.355 | 0.382 | 0.367 |

**Table S5: The primer used in this study**

| Target  vector |  | Target | Primer name | Primer sequence (5′ to 3′) |
| --- | --- | --- | --- | --- |
| Prey-AD-pGADT7 |  | *Ts*LBD30a | *Ts*LBD30a-F | TGGCCATGGAGGCCAGTGAATTCATGCCCGGTAACAGTGGT |
|  |  |  | *Ts*LBD30a-R | CTACGATTCATCTGCAGCTCGAGTCAGTCTAATTTAGAGAGTGAAAGT |
|  |  | *Ts*LBD30b | *Ts*LBD30b-F | TGGCCATGGAGGCCAGTGAATTCATGAGCGTAAATAGCAGTGG |
|  |  |  | *Ts*LBD30b-R | CTACGATTCATCTGCAGCTCGAGTCAAGTAAAGTCTCTTTTAGGAG |
|  |  | *Ts*VND6.1 | *Ts*VND6.1-F | TGGCCATGGAGGCCAGTGAATTCATGAATGTCTTTTCACATGT |
|  |  |  | *Ts*VND6.1-R | CTACGATTCATCTGCAGCTCGAGTCACTTCCACAGATCAATTT |
|  |  | *Ts*VND7.1 | *Ts*VND7.1-F | TGGCCATGGAGGCCAGTGAATTCATGGAAGTGGAGTCTTGTG |
|  |  |  | *Ts*VND7.1-R | CTACGATTCATCTGCAGCTCGAGTTACAAGTTGGGAAAGTAGC |
|  |  | *Ts*VND7.2 | *Ts*VND7.2-F | TGGCCATGGAGGCCAGTGAATTCATGGAAGTGGAGTCCTGTG |
|  |  |  | *Ts*VND7.2-R | CTACGATTCATCTGCAGCTCGAGTTACATGTTAGAAAAATGTCCAG |
| Bait-BD-pHIS2 |  | *Ts*VND7.2 | *Ts*VND7.2-F | GACTCACTATAGGGCGAATTCATTTTCTGACATTTCATTCTATCTA |
|  |  |  | *Ts*VND7.2-R | GATTCGCGAACGCGTGAGCTCTGATGATGCTGATCACCTGAG |
|  |  | *Ts*MYB-F | *Ts*MYB-F | GACTCACTATAGGGCGAATTCACGTATGAATGTACATATGAAAGAA |
|  |  |  | *Ts*MYB-R | GATTCGCGAACGCGTGAGCTCTTTGAATTTTTCTTATCCTATCCCT |
|  |  | *Ts*CesA4 | *Ts*CesA4-F | GACTCACTATAGGGCGAATTCTTATCTGTTTGTGTCCATTTGTATA |
|  |  |  | *Ts*CesA4-R | GATTCGCGAACGCGTGAGCTCGGATGGCAGATCAACAAGAAG |
|  |  | *Ts*CesA8 | *Ts*CesA8-F | GACTCACTATAGGGCGAATTCTTTTATAAAAAAAAAAAATAACTGCAAGGG |
|  |  |  | *Ts*CesA8-R | GATTCGCGAACGCGTGAGCTCCTCGACCGATTATCGTTGCA |
|  |  | *Ts*XCPa | *Ts*XCPa-F | GACTCACTATAGGGCGAATTCTGATACAGATGGATGGTCA |
|  |  |  | *Ts*XCPa-R | GATTCGCGAACGCGTGAGCTCCTCTGTTGAGTGTCGGGATTC |
|  |  | *Ts*XCPb | *Ts*XCPb-F | GACTCACTATAGGGCGAATTCATATTAATGTAATATTTGTGGGC |
|  |  |  | *Ts*XCPb-R | GATTCGCGAACGCGTGAGCTCTTGTGTTGGGATTCAGGAT |
|  |  | *Ts*XCPc | *Ts*XCPc-F | GACTCACTATAGGGCGAATTCTCGGTCAACTGTAAAATCCT |
|  |  |  | *Ts*XCPc-R | GATTCGCGAACGCGTGAGCTCTTCTTTTGGGGCTCTGGATT |
| pCAMBIA-2300-GFP |  | 35S-*Ts*VND6.1-GFP | 35S-*Ts*VND6.1-F | GGGGTACCATGAATGTCTTTTCACATGTTCCCCC |
|  |  |  | 35S-*Ts*VND6.1-R | GCGTCGACCTTCCACAGATCAATTTGGCAAC |
|  |  | 35S-*Ts*VND6.2-GFP | 35S-*Ts*VND6.2-F | GGGGTACCATGAATACCTTTACGCATGTTCCACC |
|  |  |  | 35S-*Ts*VND6.2-R | GCGTCGACCTTCCACAGATCAATTTGGCAAC |
|  |  | 35S-*At*VND6-GFP | 35S-*At*VND6-F | GGGGTACCATGGTGAACCAAAGGAGAAGAC |
|  |  |  | 35S-*At*VND6-R | GCGTCGACTCCAACGAAATTGACAATC |
|  |  | 35S-*At*VND7-GFP | 35S-*At*VND7-F | GGGGTACCATGGATAATATAATGCAATCGTCAATG |
|  |  |  | 35S-*At*VND7-R | GCGTCGACCGAGTCAGGGAAGCATCCAAGAG |
|  |  | 35S-*Ts*VND7.1-GFP | 35S-*Ts*VND7.1-F | GGGGTACCATGGAAGTGGAGTCTTGTGTC |
|  |  |  | 35S-*Ts*VND7.1-R | GCGTCGACCAAGTTGGGAAAGTAGCCAAG |
|  |  | 35S-*Ts*VND7.2-GFP | 35S-*Ts*VND7.2-F | GGGGTACCATGGAAGTGGAGTCCTGTGT |
|  |  |  | 35S-*Ts*VND7.2-R | GCGTCGACTGCCAAACTTGGAGAAGAT |
| pGreenII 0800-LUC |  | *Ts*LBD30apro-LUC | *Ts*LBD30apro-F | TCCCCCGGGCTGCAGGAATTCATGCCCGGTAACAGTGGT |
|  |  |  | *Ts*LBD30apro-R | GTCGACGGTATCGATAAGCTTTCAGTCTAATTTAGAGAGTGAAAGT |
|  |  | *Ts*LBD30bpro-LUC | *Ts*LBD30bpro-F | TCCCCCGGGCTGCAGGAATTCATGAGCGTAAATAGCAGTGG |
|  |  |  | *Ts*LBD30bpro-R | GTCGACGGTATCGATAAGCTTTCAAGTAAAGTCTCTTTTAGGAG |
|  |  | *Ts*VND6.1pro-LUC | *Ts*VND6.1pro-F | TCCCCCGGGCTGCAGGAATTCATGAATGTCTTTTCACATGT |
|  |  |  | *Ts*VND6.1pro-R | GTCGACGGTATCGATAAGCTTTCACTTCCACAGATCAATTT |
|  |  | *Ts*VND7.1pro-LUC | *Ts*VND7.1pro-F | TCCCCCGGGCTGCAGGAATTCATGGAAGTGGAGTCTTGTG |
|  |  |  | *Ts*VND7.1pro-R | GTCGACGGTATCGATAAGCTTTTACAAGTTGGGAAAGTAGC |
|  |  | *Ts*VND7.2pro-LUC | *Ts*VND7.2pro-F | TCCCCCGGGCTGCAGGAATTCATGGAAGTGGAGTCCTGTG |
|  |  |  | *Ts*VND7.2pro-R | GTCGACGGTATCGATAAGCTTTTACATGTTAGAAAAATGTCCAG |
| pGreenII 62-SK |  | pGreenII 62-SK-*Ts*VND7.1 | *Ts*VND7.1-F | AGGTCGACGGTATCGATAAGCTTCAAGGAGAACCATAATCTGCAACAT |
|  |  |  | *Ts*VND7.1-R | CGCTCTAGAACTAGTGGATCCTGAATATGTTCACCTGAGAATCATAG |
|  |  | pGreenII 62-SK-*Ts*VND7.2 | *Ts*VND7.2-F | AGGTCGACGGTATCGATAAGCTTATTTTCTGACATTTCATTCTATCTA |
|  |  |  | *Ts*VND7.2-R | CGCTCTAGAACTAGTGGATCCTGATGATGCTGATCACCTGAG |
|  |  | pGreenII 62-SK-*Ts*MYB | *Ts*MYB-F | AGGTCGACGGTATCGATAAGCTTACGTATGAATGTACATATGAAAGAA |
|  |  |  | *Ts*MYB-R | CGCTCTAGAACTAGTGGATCCTTTGAATTTTTCTTATCCTATCCCT |
|  |  | pGreenII 62-SK-*Ts*CesA4 | *Ts*CesA4-F | AGGTCGACGGTATCGATAAGCTTTTATCTGTTTGTGTCCATTTGTATA |
|  |  |  | *Ts*CesA4-R | CGCTCTAGAACTAGTGGATCCGGATGGCAGATCAACAAGAAG |
|  |  | pGreenII 62-SK-*Ts*CesA8 | *Ts*CesA8-F | AGGTCGACGGTATCGATAAGCTTTTTTATAAAAAAAAAAAATAACTGCAAGGG |
|  |  |  | *Ts*CesA8-R | CGCTCTAGAACTAGTGGATCCCTCGACCGATTATCGTTGCA |
|  |  | pGreenII 62-SK-*Ts*XCPa | *Ts*XCPa-F | AGGTCGACGGTATCGATAAGCTTTGATACAGATGGATGGTCA |
|  |  |  | *Ts*XCPa-R | CGCTCTAGAACTAGTGGATCCCTCTGTTGAGTGTCGGGATTC |
|  |  | pGreenII 62-SK-*Ts*XCPc | *Ts*XCPc-F | AGGTCGACGGTATCGATAAGCTTTCGGTCAACTGTAAAATCCT |
|  |  |  | *Ts*XCPc-R | CGCTCTAGAACTAGTGGATCCTTCTTTTGGGGCTCTGGATT |
