# Supplementary material for: The Tetracentron genome provides insight into the early evolution of eudicots and the formation of vessel elements
Source: Genome Biol. 2020 Dec 2;21:291. doi: 10.1186/s13059-020-02198-7 (PMC7709256; doi:10.1186/s13059-020-02198-7)
Supplement: Supplementary file 2 — Additional file 2. Review history. [file 13059_2020_2198_MOESM2_ESM.docx]

**1^st^ round**

**Reviewer 1**

The evolution of early eudicots and core eudicots is an interesting scientific issue. For example, core eudicots account for 75% of angiosperm species. Therefore, sequencing more early eudicots can help us further understand what happened when the early eudicots and core eudicots diversification and provide us with a deeper understanding of the core eudicot species expansion. But this article seems to want to reveal more about the early evolution of eudicot, but there are no more relevant results to show and did not dig deep.

The main problems are as follows:

1.early evolution of eudicots is really important, because the number of core eudicots species is 75% of angiosperms, and its early origin is worth further analysis. Unfortunately, there is very little research on early evolution of eudicots in this article, and no new ideas have been seen. The article said that it revealed early evolution of eudicots, but did not see the results that were not found before.

2. Especially with regard to the gamma event, if it is doubled and hybridized again according to the previous article (The Aquilegia genome reveals a hybrid origin of core eudicots), the collinear relationship between T. sinense and the grape is exactly 4: 3, and it cannot be denied that the gamma event did not participate T. sinense genome.

3. Regarding the vessels elements, it should be reported from the article that T. sinense has vessels-like elements. If vessel-like elements are not vessel elements, how did T. sinense lose vessel elements? What is the difference between this vessel-like elements and vessel elements? Another possibility is that vessel-like elements are the ancestors of vessel elements? So how do vessel-like elements become vessel elements?

4.The water lily genome has recently been sequenced. By comparing water lilies with amborella, water lilies are more like mesangiosperms. Recently, some magnolia genomes have also been sequenced. It is worth comparing the water lily and these magnolia genomes with the early eudicots for comparative genomic analysis to find more evolutionary characteristics of early evolution of eudicots.

5.Basal eudicots do not exist, they have long disappeared, and now seen early diverging lineages of eudicots is just a group after diversification of basal eudicots, so it is not appropriate to call basal eudicots.

**Reviewer 2**

The authors report the sequencing and assembly of the genome of Tetracentron sinense, a representative of the early diverging dicot lineage Trochodendrales known for lacking a key angiosperm trait, secondary xylem with vessels. Besides important insights into genome evolution in this lineage (two lineage specific whole-genome duplications and absence of the paleohexaploidy event shared by core eudicots), the authors present evidence that T. sinense in fact possesses vessel-like elements in its secondary xylem, confirming the findings of an earlier study (Ren et al. 2007) and providing further support for the notion that T. sinense may represent an intermediate form between tracheids and vessel elements.

How is the development of these "vessel-like" elements regulated? The authors present evidence that the T. sinense genome contains a putative ortholog of VND7, a master regulator of angiosperm vessel formation which appears to function in a similar transcriptional network in T. sinense than what has been well characterized in dicot models such as Arabidopsis. Similarly, the authors present evidence for the presence of other transcription factors, secondary cell wall genes and programmed cell death related genes that are commonly associated with vessel development in Angiosperms.

This paper is an important contribution to knowledge of genome biology and evolution in the early dicots. The finding of an apparently functional vessel master regulator, vessel cell wall transcriptional network and downstream vessel-related targets is novel and important for understanding the evolution of evolutionary important angiosperm specialized traits such as xylem vessels (vs tracheids in the gymnosperms).

Overall, the experimental work is expertly executed and conclusions well supported by the evidence. However, I think there is a need for greater clarity regarding the authors' hypothesis for the possible involvement of gene or genome duplications in the development of vessel-like elements in T. sinense. Is this a gain-of-function? In the discussion (line 409) the authors mention that differences in factors between A. thaliana and T. sinense may be responsible for an "anatomical reversal of tracheary elements". This is confusing because tracheary elements would be the ancestral state.

I also think the last result section, resequencing of 55 individuals of T. sinense, does not contribute much to the main topic of the paper and could be omitted without affecting the impact of the paper. The demographics of T. sinense may be the focus of a separate paper.

Below, I identify a number of issues for consideration by the authors, some of which are minor in nature:

line 41 - "known for having"

line 42 - gymnosperms

line 51 - "that vessel-like elements [are] present in T. sinense".

line 51-53. I propose that the authors split the sentence as follows: "There are two genes orthologous to the master regulator of vessel formation AtVND7. Gene and genome duplications play a role in the expansion of VNDs."

line 54 - "regulating TsVND7.2"

line 54 - "regulatory relationship"

line 55 - "Population genomics [reveals] high [levels] of genetic diversity of T. sinense and [identifies] four refugia in China.

line 72 - Except for the Buxales, Trochodendrales and the core eudicots share the most recent common ancestor. I propose that the authors redraft for clarity.

line 77 - a feature that [it shares with]

line 80 - rather than "essence" the authors can say "regulation" or "developmental biology"

Line 81 - The authors should clarify here the main hypothesis regarding the absence of vessels in the Trochodendrales. Does "reversal" here means that the hypothesis is that vessels were once present but have been lost in this Angiosperm order?

line 86 Before mentioning resequencing, the authors should include some information here on the genome (size, number of chromosomes) and cite previous genomic analyses.

line 89 - I miss an explicit statement on the main aims and hypotheses of the study. In general, the background section seems inadequate to introduce the relevant questions and cite prior work.

Line 96 - the authors should cite the genome size estimation here.

line 101 - the genome size estimation is not shown in Figure S1a or elsewhere in Additional file 1.

line 102 - The heterozygosity of the sequenced individual cannot be extrapolated to the entire species. Also, the estimation of heterozygosity is not explained in Additional file 1.

Figure 1 - The meaning of the color coding should be explained in the figure legend. Also, there seems to be some typos in the annotated divergence ages of clades (e.g. between Solanum and Coffea 47.0 - 99.32 My?)

line 190 - The authors should clarify in the text (and in Figure 3g and h) whether "the cells" here refer only to the fusiform peculiar cells or to all of the tracheary elements in T. sinense.

line 194 "hereafter" rather than "thereafter"

Fig 4a is not adequately described in the figure legend.

line 209 - Figure S4a rather than Figure 4Sa.

line 212 - It will help the reader if the authors can point out the gymnosperm sequences using a dedicated symbol or colour.

Figure S4b - the last part of the figure legend seems out of place. The "cellular localization and function of VND genes" are not shown in the figure. Also, the two down arrows are not explained in the legend.

line 220-221 This statement needs a reference(s).

Line 228 - I would say "putative orthologs" in the absence of functional evidence.

line 229 In total, ...

Figure S5b - It is very hard to read the names of the protein sequences an in some cases the text seems to be distorted.

line 231-232 - This statement is a bit confusing. It appears that in many or most clades there were sequences from two or three of the species.

line 245 - Congruent with...

line 258 nucleus vs nuclear - also it appears that there is some cytoplasm localization for all of the proteins?

line 259 - bright field

line 261 - It is not clear here why TsVND6.1 was used to represent the VNDs. Can it really represent all of the VNDs? And why not use TsVND7.1 or TsVND7.2?

Figure 269 - found not to interact with…

line 269-272 and Figure S6c. The Y1H results seem correct and in agreement with prior findings in Arabidopsis, but some some caution should be noted that false negatives are possible with Y1H experiments, especially when the full length and structure of the promoters are not known and when other, plant-specific factors are required for binding. A second line of evidence (e.g. trans-activation assays) would have been good to support the proposed networks. In the absence of such evidence, it would be prudent to present the networks as hypotheses to be confirmed by additional experiments.

line 275 - 281 - The VNS gene family does not seem to be expanded in T. sinense. The proportion of genes generated by tandem duplication vs WGD seem similar to most other dicot plant lineages. I think this section does not contribute much to the overall paper.

line 283 - 307 The section on resequencing of 55 individuals representing six populations does not add to the central question and story of this paper and could be omitted. Also the number of individuals per population may be too low for robust population genomics inferences.

line 362 - The authors should add a reference here to previous findings that at least one gymnosperm lineage (gnetophytes) contains vessels. How would that affect the interpretation of their results? Could those gymnosperms similarly have intermediary forms of the transcriptional network and a functional VND7 homolog?

line 369 - from the Results text and Figure 3 it is not clear whether the authors mean that ALL tracheary elements in T. sinense are "vessel-like", or only some of the tracheary elements (the peculiar cells?).

line 391 - directly regulate many genes

line 396 - that these genes belong to originated before the divergence of the angiosperms. We found that...

line 409 - It is not clear what is meant by "anatomical reversal" here. The cells either develop into tracheids or vessel elements. Also, is the suggestion here that there is a reversal from tracheary elements to vessel elements? This would not make sense since tracheary elements are the ancestral state.

line 417 -419 - This statement does not really follow from the previous sentences. Are the authors suggesting that duplication of specific genes already involved in tracheary element development resulted in the conversion of tracheary elements into vessel-like elements? Is it linked to the functional divergence of the two VND7 paralogs in T. sinense?

Line 421 - 450 This section can be omitted. It does not really add much to the main theme of the paper and may in fact detract from the main focus. There is also no critical discussion of sample size and whether 55 individuals can represent the diversity and dynamics of 6 populations. I would suggest expanding that part of the work and publishing the resequencing analysis and population genomics in a separate focused paper.

line 466 - Are the authors suggesting here that gene or genome duplication resulted in a "gain of function" that caused a transition from tracheary elements to vessel-like elements?

line 488 - please give a reference for GCE software.

line 529 and 532 - it is not clear what manual adjustments were made to the assembly and what improvements resulted.

line 665-666 What length promoter fragment was cloned and did it include the 5'UTR?

**Author response**

Reviewer 1

The evolution of early eudicots and core eudicots is an interesting scientific issue. For example, core eudicots account for 75% of angiosperm species. Therefore, sequencing more early eudicots can help us further understand what happened when the early eudicots and core eudicots diversification and provide us with a deeper understanding of the core eudicot species expansion. But this article seems to want to reveal more about the early evolution of eudicot, but there are no more relevant results to show and did not dig deep.

**Question 1:** early evolution of eudicots is really important, because the number of core eudicots species is 75% of angiosperms, and its early origin is worth further analysis. Unfortunately, there is very little research on early evolution of eudicots in this article, and no new ideas have been seen. The article said that it revealed early evolution of eudicots, but did not see the results that were not found before.

**Reply:** We thank you for the expert comments here, which prompted us to dig deeper into the early evolution of eudicots. As suggested, we compared the genomes of water lily, one Magnoliid species *Cinnamomum* *kanehirae*, along with the two early diverging eudicot species (*Aquilegia coerulea* and *Nelumbo nucifera*) and the two species of Trochodendrales (*T. sinense/T. aralioides*). Extensive inter- and intra-genomic comparisons including synteny, *Ks*, and phylogenomic analyses were made to give further insight into the early evolutionary history of eudicots. The new analyses (Figure 2 and Figure S8, 9 in Additional file 1) revealed that the WGDs that occurred in *A. coerulea*, *N. nucifera*, and the two species of Trochodendrales are lineage-specific, and the gamma paleohexaploidy event was not shared with Trochodendrales (lines 231-280).

We extensively analyzed the evolution of gene families during the early diversification of eudicots (lines 192-216). By employing CAFE (De Bie et al., 2006), five times as many gene families expanded in the lineage leading to all eudicots (551), as compared to core eudicots (110). However, twice as many gene families contracted in the lineage leading to core eudicots compared with the lineage leading to all eudicots (Figure 1 and Figure S4 in Additional file 1). Although there are differences in the flower structure between early diverging eudicots and core eudicots, the ABCE gene families did not expand and contract in the lineages leading to eudicots and core eudicots, suggesting that the copy number of ABCE model genes may not contribute to the flower structure difference between early diverging eudicots and core eudicots. When the rapidly evolved gene families to lineage of *T. sinense* or *T. aralioides* were used to perform GO and KEGG enrichment (Figure S6 and Table S2 in Additional file 1), we found that there are many genes enriched in defense, toxin catabolic process, response to salicylic acid, and oxidative stress (Figure S5 and Table S2 in Additional file 1).

In making inter- and intra-genomic comparisons about the genomic structure and *Ks* analyses, we found that the nucleotide substitution rate of *T. sinensis* is much lower than that of *N. nucifera* and *V. vinifera* (Figure 2e)*.* Extensive collinearity within itself, as well as with other plant genomes, makes *T. sinense* a good reference for comparative genomic analysis.

With these analyses, we added the new data into the current version and revised the original manuscript (lines 191-215, 231-280 431-458 and 459-479).

**Question 2:** Especially with regard to the gamma event, if it is doubled and hybridized again according to the previous article (The Aquilegia genome reveals a hybrid origin of core eudicots), the collinear relationship between *T. sinense* and the grape is exactly 4: 3, and it cannot be denied that the gamma event did not participate T. sinense genome.

**Reply:** We thank the reviewer for this question about the process of the gamma event, and we agree with the reviewer that the collinear relationship between *T. sinense* and grape is 4: 3 no matter whether the gamma (part of gamma) was shared by *T. sinense*. Below we provided additional solid evidence to support the idea that the gamma event was not shared with *T. sinense.*

(1) We performed additional inter-genomic comparisons using several other outgroup species, including one basal angiosperm (*N. colorata*), one magnoliid (*C. kanehirae*), and two early diverging eudicots (*A. coerulea*, *N. nucifera*)*.* The genomic collinearity relationship showed a 4: 2, 4:4 syntenic pattern between *T. sinense/T. aralioides and N. colorata*, and between *T. sinense*/*T. aralioides* and *C. kanehirae* (Figure 2b, c and Figure S8c, d, e, f in Additional file 1). Given the existence of one *N. colorata* lineage-specific WGD and two *C. kanehirae* lineage-specific WGDs (Zhang et al., 2020; Chaw et al., 2019), our results suggested two WGDs in Trochodendrales occurred after the divergence of magnoliids. In addition, the synteny relationship between *N. colorata* and *N. nucifera*, *C. kanehirae* and *N. nucifera* is also 2:2 and 4:2 (Figure S8g, h in Additional file 1), indicating one WGD in *N. nucifera* after the divergence from magnoliids*.* Several earlier studies showed that the WGD in *N. nucifera* is lineage-specific rather than eudicot-wide (Ming et al., 2013; Gui et al., 2018; Shi et al., 2020), suggesting that the eudicot-wide WGD proposed by Akoez and Nordborg (2019) is likely inappropriate, and the two WGDs in *T. sinense/T. aralioides* seem to be lineage-specific.

(2) We carried out *Ks*-based dating analyses to confirm the timing and nature of these WGDs. As shown in Figure 2e, the peaks of *K*s distribution of *T. sinense/T. aralioides* paralogs (retained from two WGD events) are much smaller than those of orthologs comparing *T. sinense/T. aralioides* to *A. coerulea* and to *N. nucifera* respectively, strongly suggesting the two WGDs in *T. sinense/T. aralioides* occurred after their divergence from *A. coerulea* and *N. nucifera*. In addition, the *N. nucifera* paralogs also displayed a lower peak *Ks* value than orthologs comparing *N. nucifera* to *T. sinense/ T. aralioides* and to *A. coerulea*, further confirming that the WGD in *N. nucifera* is lineage-specific. Moreover, our absolute dating of the paralogues of *T. sinense*/*T. aralioides* in the original manuscript revealed that the two WGDs identified in the genomes of Trochodendrales occurred approximately 87–78 and 59–54 MYA, suggesting that the two WGDs occurred after the divergence of Trochodendrales from Proteales (*N. nucifera*) (approximately 125 MYA).

(3) We performed integrated syntenic and phylogenomic analyses to confirm the timing of these WGDs in early diverging eudicots. We obtained those anchor genes from the inter-genomic synteny blocks with ratio of 1:2:4 in *A. trichopoda*, *N. nucifera*, and *T. sinense*. All genes in the same sets of syntenic block were concatenated and used to construct phylogenetic trees. In total, we obtained 31 groups of concatenated genes (a total of 94 genes) and 87% of the trees based on the concatenated genes supported the idea that the WGDs in *N. nucifera* and *T. sinense* are lineage specific.

Based on all the results mentioned above, we can safely conclude that tetraploidy did not occur in the ancestry of all eudicots and the gamma event was not shared with the *T. sinense* genome (lines 231-280 and lines 431-458).

**Question 3:** Regarding the vessel elements, it should be reported from the article that *T. sinense* has vessels-like elements. If vessel-like elements are not vessel elements, how did *T. sinense* lose vessel elements? What is the difference between this vessel-like elements and vessel elements? Another possibility is that vessel-like elements are the ancestors of vessel elements? So how do vessel-like elements become vessel elements?

**Reply:** We apologize for the improper definition in the original manuscript. In the past few weeks, we showed the macerated tracheary elements, SEM photographs, and three-dimensional sections (Figure 3) to the plant anatomists at Peking University and the Institute of Botany in Chinese Academy of Sciences, as well as wood anatomists in the Chinese Academy of Forestry, and had many discussions of the term used here. All the experts disagreed with using “vessel-like elements” and suggested we should use “vessel elements”, as reported in previous publications (Ren et al., 2007; Li et al., 2011). In particular, we found this species has VND7, which is required for vessel formation (Figure 3 and Figure S11 in additional file). In the revised manuscript, we therefore changed “vessel-like elements” to “vessel elements” in this part and revised the related sentences accordingly.

**Question 4:** The water lily genome has recently been sequenced. By comparing water lilies with *amborella*, water lilies are more like mesangiosperms. Recently, some magnolia genomes have also been sequenced. It is worth comparing the water lily and these magnolia genomes with the early eudicots for comparative genomic analysis to find more evolutionary characteristics of early evolution of eudicots.

**Reply**: Thank you for your excellent suggestions. Indeed, the genomes of water lily and several magnoliid species were published recently and are helpful to clarify the early evolution of eudicots. In the course of revising the manuscript, we compared the water lily and *C. kanehirae* genomes with the early diverging eudicots for dating the gamma event as well as for characterizing the gene family evolution. We used *C. kanehirae* to represent Magnoliids because it is the sole available genome with chromosome-level assembly in the NCBI public database. The new results were incorporated into the revised manuscript (lines 232-281, lines 196-216 and lines 431-479) as shown in the responses to question 1 and 2.

**Question 5:** Basal eudicots do not exist, they have long disappeared, and now seen early diverging lineages of eudicots is just a group after diversification of basal eudicots, so it is not appropriate to call basal eudicots.

**Reply:** Thanks for your careful explanation of the problem. We changed the terminology of “basal eudicots” to “early diverging lineages of eudicots” in the revised manuscript.

**References**

Akoez G, Nordborg M. The *Aquilegia* genome reveals a hybrid origin of core eudicots. *Genome Biol*. 2019; 20:1.

Chaw S-M, Liu Y-C, Wu Y-W, Wang H-Y, Lin C-YI, Wu C-S, et al. Stout camphor tree genome fills gaps in understanding of flowering plant genome evolution. Nature Plants 2019; 5(1):63-73.

De Bie T, Cristianini N, Demuth JP, Hahn MW. CAFE: a computational tool for the study of gene family evolution. Bioinformatics 2006; 22(10):1269-1271.

Gui S, Peng J, Wang X, Wu Z, Cao R, Salse J, Zhang H, Zhu Z, Xia Q, Quan Z et al. Improving *Nelumbo* nucifera genome assemblies using high-resolution genetic maps and BioNano genome mapping reveals ancient chromosome rearrangements. Plant J. 2018; 94(4):721-734.

Li H-F, Chaw S-M, Du C-M, Ren Y. Vessel elements present in the secondary xylem of *Trochodendron* and *Tetracentron* (Trochodendraceae). Flora 2011; 206(6):595-600.

Ming R, Leebens-Mack J, Ayyampalayam S, Bowers JE, McKain MR, McNeal J, et al. Genome of the long-living sacred lotus (*Nelumbo nucifera* Gaertn.). Genome Biol. 2013; 14: R41.

Ren Y, Chen L, Tian XH, Zhang XH, Lu AM*.* Discovery of vessels in *Tetracentron* (Trochodendraceae) and its systematic significance. Plant Syst Evol. 2007; 267;155–61.

Shi T, Rahmani RS, Gugger PF, Wang M, Li H, Zhang Y, et al. Distinct expression and methylation patterns for genes with different fates following a single whole-genome duplication in flowering plant. Mol. Biol. Evol. 2020.

Zhang L, Chen F, Zhang X, Li Z, Zhao Y, Lohaus R et al. The water lily genome and the early evolution of flowering plants. Nature 2020;577(7788):79-+.

Reviewer 2

The authors report the sequencing and assembly of the genome of *Tetracentron sinense*, a representative of the early diverging dicot lineage Trochodendrales known for lacking a key angiosperm trait, secondary xylem with vessels. Besides important insights into genome evolution in this lineage (two lineage specific whole-genome duplications and absence of the paleohexaploidy event shared by core eudicots), the authors present evidence that *T. sinense* in fact possesses vessel-like elements in its secondary xylem, confirming the findings of an earlier study (Ren et al. 2007) and providing further support for the notion that *T. sinense* may represent an intermediate form between tracheids and vessel elements.

How is the development of these "vessel-like" elements regulated? The authors present evidence that the *T. sinense* genome contains a putative ortholog of VND7, a master regulator of angiosperm vessel formation which appears to function in a similar transcriptional network in T. sinense than what has been well characterized in dicot models such as *Arabidopsis*. Similarly, the authors present evidence for the presence of other transcription factors, secondary cell wall genes and programmed cell death related genes that are commonly associated with vessel development in Angiosperms.

This paper is an important contribution to knowledge of genome biology and evolution in the early dicots. The finding of an apparently functional vessel master regulator, vessel cell wall transcriptional network and downstream vessel-related targets is novel and important for understanding the evolution of evolutionary important angiosperm specialized traits such as xylem vessels (vs tracheids in the gymnosperms).

Overall, the experimental work is expertly executed and conclusions well supported by the evidence. However, I think there is a need for greater clarity regarding the authors' hypothesis for the possible involvement of gene or genome duplications in the development of vessel-like elements in *T. sinense*. Is this a gain-of-function? In the discussion (line 409) the authors mention that differences in factors between *A. thaliana* and *T. sinense* may be responsible for an "anatomical reversal of tracheary elements". This is confusing because tracheary elements would be the ancestral state.

I also think the last result section, resequencing of 55 individuals of T. sinense, does not contribute much to the main topic of the paper and could be omitted without affecting the impact of the paper. The demographics of T. sinense may be the focus of a separate paper.

**Reply:** Many thanks for the positive comments and we are delighted to see your conclusion that the experimental work is expertly executed and the conclusions well supported by the evidence. We found the suggestions valuable and we carried out new experiments, using luciferase activity assays, to make a satisfactory reply.

Below, I identify a number of issues for consideration by the authors, some of which are minor in nature:

**Question 1:** line 41 - "known for having"

**Reply:** We have revised as suggested (line 40).

**Question 2:** line 42 – gymnosperms

**Reply:** The sentence has been deleted.

**Question 3:** line 51 - "that vessel-like elements [are] present in *T. sinense*".

**Reply:** This has been corrected as suggested (line 50).

**Question 4:** line 51-53. I propose that the authors split the sentence as follows: "There are two genes orthologous to the master regulator of vessel formation AtVND7. Gene and genome duplications play a role in the expansion of VNDs."

**Reply:** We have revised the sentence (lines 50-51).

**Question 5:** line 54 - "regulating TsVND7.2"

**Reply:** We changed it as suggested (line 51).

**Question 6:** line 54 - "regulatory relationship"

**Reply: “**regulation relationship” has been changed to "regulatory relationship" (line 52).

**Question 7:** line 55 - "Population genomics [reveals] high [levels] of genetic diversity of T. sinense and [identifies] four refugia in China.

**Reply:** This has been corrected by Plant Editors (line 53).

**Question 8:** line 72 - Except for the Buxales, Trochodendrales and the core eudicots share the most recent common ancestor. I propose that the authors redraft for clarity.

**Reply:** We reworded these sentences to make them clearer (Lines 72-73). “Within eudicots, the four extant eudicot lineages that diverged the earliest are the Ranunculales, Proteales, Trochodendrales, and Buxales.”

**Question 9:** line 77 - a feature that [it shares with]

**Reply:** The sentence has been deleted.

**Question 10:** line 80 - rather than "essence" the authors can say "regulation" or "developmental biology"

**Reply:** The sentence has been deleted.

**Question 11**: Line 81 - The authors should clarify here the main hypothesis regarding the absence of vessels in the Trochodendrales. Does "reversal" here means that the hypothesis is that vessels were once present but have been lost in this Angiosperm order?

**Reply:** Yes, we agree with the comment. In the revised manuscript, we changed the vessel-like element into vessel elements in this part, because we found VND7 (the master regulator of vessel formation) is present in the species (Figure 3 and Figure S11). Moreover, we showed the results of macerated tracheary elements, SEM photographs, and three-dimensional sections (Figure 3) to the plant anatomists at Peking University and the Institute of Botany in Chinese Academy of Sciences as well as wood anatomists at the Chinese Academy of Forestry, and discussed the term used here. All the experts agreed that we should not use “vessel-like elements” and suggested we should use “vessel elements”, as reported in previous publications (Ren et al., 2007; Li et al., 2011). Therefore, we deleted the sentence in the original manuscript since it is inappropriate.

**Question 12:** line 86 Before mentioning resequencing, the authors should include some information here on the genome (size, number of chromosomes) and cite previous genomic analyses.

**Reply:** Thank you for your suggestion. Since there was no information on genome size of this species previously, we added only the number of chromosomes. We also introduced and cited some previous studies on population genetics of *T. sinense* before mentioning resequencing (lines 103-106 and 108-112)

**Question 13:** line 89 - I miss an explicit statement on the main aims and hypotheses of the study. In general, the background section seems inadequate to introduce the relevant questions and cite prior work.

**Reply**: We agreed with the comments and suggestions are very helpful. The main aims of this study are: (1) to explore what happened in the genomes following the early evolution of eudicots through a genomic study comparing *T. sinense* with early diverging angiosperms, magnoliids, and other early diverging core eudicots; (2) to investigate whether there are vessel elements in *T. sinense*, whether the ortholog of master regulator of vessel element formation VND7 is present in *T. sinense* and whether its regulatory network functions as in *A. thaliana*; (3) to investigate the genetic diversity and evolutionary history of *T. sinense*. We added an explicit statement of these aims into the revised manuscript and cited prior work accordingly (lines 65-121).

**Question 14:** Line 96 - the authors should cite the genome size estimation here.

**Reply:** Yes, we agree with the suggestion and we cited the genome size as suggested (lines 134-137).

**Question 15:** line 101 - the genome size estimation is not shown in Figure S1a or elsewhere in Additional file 1.

**Reply:** We apologize for not showing the genome size estimation in Figure S1. As suggested, we added one paragraph about the estimation of genome size and heterozygosity in Additional file 1 (lines 65-90).

**Question 16:** line 102 - The heterozygosity of the sequenced individual cannot be extrapolated to the entire species. Also, the estimation of heterozygosity is not explained in Additional file 1.

**Reply:** Thank you very much for pointing out this problem. We have changed “the heterozygosity of the species” to “the heterozygosity of the sequenced individual” in the revised manuscript (lines 135-136). Moreover, we added a paragraph about the estimation of genome size and heterozygosity in Additional file 1.

**Question 17:** Figure 1 - The meaning of the color coding should be explained in the figure legend. Also, there seems to be some typos in the annotated divergence ages of clades (e.g. between Solanum and Coffea 47.0 - 99.32 My?)

**Reply:** Thank you for the careful reading. We have revised Figure 1. The meaning of the color coding has been added in the figure legend (page 40).

**Question 18**: line 190 - The authors should clarify in the text (and in Figure 3g and h) whether "the cells" here refer only to the fusiform peculiar cells or to all of the tracheary elements in *T. sinense*.

- **Reply:** We apologize for the unclear explanation here. The cells in Figure 3g and h referred to many tracheary elements rather than to the fusiform peculiar cells in *T. sinense*. Therefore, we changed the sentence to “many tracheary elements often have scalariform bordered pits on their radial walls” (line 289)

**Question 19:** line 194 "hereafter" rather than "thereafter"

**Reply:** The relevant sentence was deleted in the revised manuscript.

**Question 20:** Fig 4a is not adequately described in the figure legend.

**Reply:** We have added more descriptions to the figure legend of Fig 4a (page 46) “A maximum likelihood tree of genes from the *VNS* gene family. Bootstrap percentages for the large clades are shown next to the nodes. Species name abbreviations given in Figure S11 in additional file 1. Angiosperm sequences of different subfamilies are shown in different colors and the gymnosperm sequences are shown in black.”

**Question 21:** line 209 - Figure S4a rather than Figure 4Sa.

**Reply:** This has been revised (line 307).

**Question 22:** line 212 - It will help the reader if the authors can point out the gymnosperm sequences using a dedicated symbol or colour.

**Reply:** Thank you for your suggestion. We pointed out the gymnosperm sequences are shown in black (lines 309-310).

**Question 23:** Figure S4b - the last part of the figure legend seems out of place. The "cellular localization and function of VND genes" are not shown in the figure. Also, the two down arrows are not explained in the legend.

**Reply:** We deleted these words in the revised manuscript. We also explained the two down arrows (Figure 11b in revised Additional file 1, marked in blue).

**Question 24:** line 220-221 This statement needs a reference(s).

**Reply:** As suggested, a reference (Soyano et al., 2008) has been added (line 319).

**Question 25:** Line 228 - I would say "putative orthologs" in the absence of functional evidence.

**Reply:** We agreed and now “orthologous” has been changed to “putative orthologs” (line 325)

**Question 26:** line 229 In total, ...

**Reply:** “Totally” has been changed to “In total” (line 327)

**Question 27:** Figure S5b - It is very hard to read the names of the protein sequences an in some cases the text seems to be distorted.

**Reply:** Yes, this is indeed a problem. Now we have reconstructed the Figure S5b (Figure S12b in the revised Additional file 1) and the names of the protein sequences are better presented now.

**Question 28:** line 231-232 - This statement is a bit confusing. It appears that in many or most clades there were sequences from two or three of the species.

**Reply:** Sorry for the unclear description here. Now we changed the sentence to “All subgroups (except for 2 subgroups only including sequences from *A. thaliana*) contain sequences from *A. thaliana/T. sinense* and *O. sativa*, suggesting these subgroups originated before the splitting of monocots and dicots” (lines 330-331).

**Question 29:** line 245 - Congruent with...

**Reply:** “In well agreement with” has been changed to “Congruent with” (line 344)

**Question 30:** line 258 nucleus vs nuclear - also it appears that there is some cytoplasm localization for all of the proteins?

**Reply:** We have changed the ‘nuclear’ to ‘nucleus’ in the revised manuscript (line 357). Since we employed the 35S promoter (rather than the native promoter) to drive *Ts*VND genes for transient expression experiments, the over-expressed *Ts*VNDs may locate not only in the nucleus but in the cytoplasm as well. The fluorescent signal of GFP largely accumulated in the nucleus instead of in the cytoplasm; therefore, we believed that the sublocalization of *Ts*VNDs are in the nucleus, as reported in Arabidopsis.

**Question 31:** line 259 - bright field

**Reply:** It has been changed as suggested (line 358).

**Question 32:** line 261 - It is not clear here why TsVND6.1 was used to represent the VNDs. Can it really represent all of the VNDs? And why not use TsVND7.1 or TsVND7.2?

**Reply:** Sorry for the unclear explanation here. We delete this confusing sentence in the revised manuscript. In fact, we selected and focused on *TsVND7.1* and *TsVND7.2*. During the experiments, we tested whether *TsVND7.1/TsVND7.2* can be regulated by TsVND6.1, TsLAB30a and TsLAB30b, and then whether TsVND7.1/TsVND7.2 can regulate *MYB*, *CES4*, *CES8*, *XCPa*, *XCPb*, and *XCPc*. TsVND6.1 was used to represent the upstream regulators, because a maximum likelihood tree of genes from the *VNDs* gene family showed that *TsVND6.1* is homologous to *AtVND4-6* and a previous study showed that *AtVND4-6* positively regulated *AtVND7.* Our yeast one-hybrid and luciferase activity assays showed that TsVND6.1 indeed positively regulates *TsVND7*.

**Question 33:** Figure 269 - found not to interact with…

**Reply:** Now “found to be not interact with” has been changed to “did not interact with” as corrected by Plant Editors (line 368).

**Question 34:** line 269-272 and Figure S6c. The Y1H results seem correct and in agreement with prior findings in Arabidopsis, but some caution should be noted that false negatives are possible with Y1H experiments, especially when the full length and structure of the promoters are not known and when other, plant-specific factors are required for binding. A second line of evidence (e.g. trans-activation assays) would have been good to support the proposed networks. In the absence of such evidence, it would be prudent to present the networks as hypotheses to be confirmed by additional experiments.

**Reply:** Thank you for the expert suggestion. As suggested, we carried out a new experiment using luciferase activity assays. The new results not only supported the interactions between TsVND7.2 and relevant genes as shown by Y1H, but also identified the interaction between TsVND7.2 and *TsLAB30b*/*TsCesA8*, which was not found in the Y1H assay. In addition, we identified an interaction between TsVND7.1 and other genes as *TsVND7.2*. False negatives in Y1H experiments and the functional divergence between *TsVND7.1* and *TsVND7.2* may contribute to the discordance between Y1H experiments and luciferase activity assays. Overall, both lines of evidence suggested that the regulatory network of *VNDs* and relevant genes is largely similar to that in *A. thaliana.*

**Question 35:** line 275 - 281 - The VNS gene family does not seem to be expanded in *T. sinense*. The proportion of genes generated by tandem duplication vs WGD seem similar to most other dicot plant lineages. I think this section does not contribute much to the overall paper.

**Reply:** We agree with this comment and have removed this part from the manuscript.

**Question 36:** line 283 - 307 The section on resequencing of 55 individuals representing six populations does not add to the central question and story of this paper and could be omitted. Also the number of individuals per population may be too low for robust population genomics inferences.

**Reply:** Thank you for the suggestion. In the decision letter, the editor suggested we should extend the analysis further to improve its contribution to the central story. Therefore, we maintained this part and carried out more analyses. For example, by Mantel test analysis, we found a significant positive correlation between the genetic and geographic distance (*r* = 0.8417, *p* = 0.004). The Treemix v1.13. analysis suggested that the TS population from the Qinling Mountains in north-central China is the result of admixture between the SC population near Sichuan Basin and the SX population from Qinling Mountains in the North and East parts of central China. Accordingly, we revised this part in the manuscript (lines 398-405).

**Question 37:** line 362 - The authors should add a reference here to previous findings that at least one gymnosperm lineage (gnetophytes) contains vessels. How would that affect the interpretation of their results? Could those gymnosperms similarly have intermediary forms of the transcriptional network and a functional VND7 homolog?

**Reply:** Thank you for your expert suggestion. We added a reference in the revised manuscript (lines 484 and 499-504). In gnetophytes, there are vessel-like water-conducting cells in the secondary xylem. However, there has been a longstanding debate about whether gnetophyte 'vessels' are homologous to the 'vessels' of angiosperms. *Gnetum montanum* is a species of gnetophytes and no orthologues of *VND7* or *VND1-3* in the sister clade were identified in the genome of *G. montanum*. Since vessel formation in angiosperms requires genes from the *VND7* clade while gymnosperms (including gnetophytes) lack orthologues of *VND7*, we believe that the molecular mechanisms underpinning the origin and development of vessels in Gnetum and angiosperms are different, and vessels in gnetophytes and angiosperms are convergent characters.

**Question 38:** line 369 - from the Results text and Figure 3 it is not clear whether the authors mean that ALL tracheary elements in *T. sinense* are "vessel-like", or only some of the tracheary elements (the peculiar cells?).

**Reply:** We apologize for the unclear description here. From our observations, only some of the peculiar tracheary elements possess end walls, and most of the tracheary elements do not have end walls as reported previously (Ren et al, 2007; Li et al., 2011). In the revised manuscript, we reworded the sentence to make it clearer (lines 491-492).

**Question 39:** line 391 - directly regulate many genes

**Reply:** We have changed “regulated” to “regulate” as suggested (line 513)

**Question 40:** line 396 - that these genes belong to originated before the divergence of the angiosperms. We found that...

**Reply:** Thank you for the careful reading. In the new version, “of these genes belonged to originated before the divergence of the angiosperms, we found that...” has been changed to “that these genes belong to originated before the divergence of the angiosperms. We found that…” (lines 517-518)

**Question 41:** line 409 - It is not clear what is meant by "anatomical reversal" here. The cells either develop into tracheids or vessel elements. Also, is the suggestion here that there is a reversal from tracheary elements to vessel elements? This would not make sense since tracheary elements are the ancestral state.

**Reply:** Thank you for pointing this out––we did not use the term properly in the original manuscript. The anatomical reversal was a previous explanation for the observation that *T. sinense* lacks vessels. According to anatomical observations in two previous studies (Ren et al, 2007) and our present results, the vessel elements are indeed present in *T. sinense*. In particular, the *VND7* gene, which is required for vessel formation, is present in this species. Therefore, we revised the conclusion and deleted this sentence (lines 528-533).

**Question 42:** line 417 -419 - This statement does not really follow from the previous sentences. Are the authors suggesting that duplication of specific genes already involved in tracheary element development resulted in the conversion of tracheary elements into vessel-like elements? Is it linked to the functional divergence of the two VND7 paralogs in *T. sinense*?

**Reply:** We agree that this statement (line 417-419) does not really follow from the previous sentence. Since we do not have solid evidence about the role of duplication, the sentence was removed from the revised manuscript.

**Question 43:** Line 421 - 450 This section can be omitted. It does not really add much to the main theme of the paper and may in fact detract from the main focus. There is also no critical discussion of sample size and whether 55 individuals can represent the diversity and dynamics of 6 populations. I would suggest expanding that part of the work and publishing the resequencing analysis and population genomics in a separate focused paper.

**Reply:** Thanks very much for your suggestion. In the decision letter, the editor suggested that we keep this part and extend the analysis (lines 551-566). By Mantel test analysis, we found a significant positive correlation between the genetic and geographic distance (r = 0.8417, p = 0.004; Figure 5e). Treemix v1.13. analysis suggested that the TS population from Qinling Mountains in north-central China is the result of admixture between the SC population near Sichuan Basin and the SX population from Qinling Mountains in the North and East parts of central China (Figure 5f).

**Question 44:** line 466 - Are the authors suggesting here that gene or genome duplication resulted in a "gain of function" that caused a transition from tracheary elements to vessel-like elements?

**Reply:** Since we no longer used “vessel-like element” to describe the peculiar fusiform cells, the relevant sentences have been deleted and the conclusion has been reworded (lines 591-594).

**Question 45:** line 488 - please give a reference for GCE software.

**Reply:** We agreed with the suggestion and added a reference for GCE software (Liu et al., 2012) (line 616).

**Question 46:** line 529 and 532 - it is not clear what manual adjustments were made to the assembly and what improvements resulted.

**Reply:** We apologize for the unclear explanation here. To improve the chromosome-length assembly, we made manual adjustment with Juicebox (Durand et al., 2016), including correcting misjoins, changing the orders and orientation of draft scaffolds with translocation and inversion error, and adjusting chromosome boundaries (lines 660-661). Figure S2 shows the contact map before and after manual adjustments. As shown by the contact maps, the breaks in the bright band of elevated contact frequency along the diagonal indicate misjoins, vertical and symmetric horizontal bowtie-ish enrichment motifs away from the diagonal indicate errors in the order of draft scaffolds (translocation), and bowtie enrichment motifs in parallel to the diagonal indicate inversion errors obvious in the draft assembly. These errors were corrected manually in the final assembly.

**Question 47:** line 665-666 What length promoter fragment was cloned and did it include the 5'UTR?

**Reply:** According to previous studies, the promoter regions of *AtMYB83* (from -191 to -68 bp), *AtCes4* (-367 to -71 bp), *AtXCP1*(from -211 to -112 bp), *AtXCP2* (from -191 to -68 bp), are less than 500 bp in length (from the translation start site). In our analysis, a 500 bp fragment upstream of the translation start site of these genes was cloned (lines 827-828). The 5' UTR was located immediately upstream of the translation start site; therefore, the 5'UTR should be included in these sequences of promoter fragments.

**References**

Durand NC. Robinson JT, Shamim MS, Machol I, Mesirov JP, Lander ES, et al. Juicebox provides a visualization system for Hi-C contact maps with unlimited zoom. Cell Syst. 2016; 3:99–101.

Li H-F, Chaw S-M, Du C-M, Ren Y. Vessel elements present in the secondary xylem of Trochodendron and Tetracentron (Trochodendraceae). Flora 2011; 206(6):595-600.

Liu B, Shi Y, Yuan Y, Hu X, Zhang H, Li N, et. al. Estimation of genomic characteristics by analyzing k-mer frequency in de novo genome projects [J]. arXiv. 2012; 1308.

Ren Y, Chen L, Tian XH, Zhang XH, Lu AM. Discovery of vessels in *Tetracentron* (Trochodendraceae) and its systematic significance. Plant Syst Evol. 2007; 267;155–61.

Soyano T, Thitamadee S, Machida Y, Chua NH. *ASYMMETRIC LEAVES2*-*LIKE19*/*LATERAL* *ORGAN BOUNDARIES DOMAIN30* and *ASL20*/*LBD18* regulate tracheary element differentiation in *Arabidopsis*. Plant Cell 2008; 20:3359–73.

Wan T, Liu Z-M, Li L-F, Leitch AR, Leitch IJ, Lohaus R et al. A genome for gnetophytes and early evolution of seed plants. Nature Plants 2018; 4(2):82-89.

**2^nd^ round**

**Reviewer 1**

This revised draft has basically solved the question I raised.

There are some small suggestions,

For example, fig1 should put fig 1b in front, as fig 1a, a picture of Tetracentron sinense flower as fig 1b, and the species tree as fig 1c.

The fig 2h can easily lead to misunderstanding, so it is recommended to remove it.

**Reviewer 2**

I have reviewed the extensive revisions made by the authors in response to my comments and that of the other reviewer. These have greatly improved the manuscript and provided greater clarity on the implications of the genome sequence for understanding the early evolutionary history of the eudicots. The section on the observation of vessels in T. sinense supported by the presence of key regulators and transcriptional interactions among these regulators and with downstream secondary cell wall genes similar to the well-described network in Arabidopsis thaliana, has also been greatly improved. Finally, I am grateful that the editor recommended that the authors retain and expand the population genomics section.

I only have a few minor comments on the revised manuscript.

Figure S1 - delete "and Hi-C Interaction heatmap". The Hi-C interaction map has moved to Figure S2.

Figure S2 - The legend refers to an arrow which is not shown in the figure.

line 149 - This should refer to Figure S2b

Figure S6 - The rationale of including the gene families that have undergone contraction is not clear in this figure.

Figure S5a and c - please annotate y-axis. Are these gene numbers in families or numbers of gene families?

Figure S4 legend - it will help the reader here to shortly state the criteria for being considered "rapidly evolved gene family". Also insert "and" before "rapdly" and change to "families".

line 288 - The statement is still not clear. "many tracheary elements often have". I think the authors can go with either "many" or "often", but not both. In general the relative frequency of normal tracheary elements vs atypical tracheary elements (with scalariform bordered pits) vs peculiar, shorter (vessel) elements is not clear from the text or the figure, but perhaps this can be quantified in a future study. Are the authors suggesting the presence of these three classes of cells? In their response the authors stated " The cells in Figure 3g and h referred to many tracheary elements rather than to the fusiform peculiar cells in T. sinense" which suggest to me that there are three types of tracheary elements. This is not made clear in the Discussion.

line 290 - The section concludes with a statement on the presence of "vessel elements", but I think the authors should also conclude about the "normal" cells. Are they true tracheary elements, or do they all exhibit atypical features (line 289)? Are two parallel developmental pathways active and do the vessel elements play an active role in water transport? Are they evolutionary relics or are they emerging in this lineage as functional water transporting elements?

line 283 and 286 - Can the authors here cite the diameter and length distribution of these peculiar cells compared to that of the normal cells? Figure 3 does not specifically point out the diameter and length of the normal cells.

Figure 3a - It is not clear here whether the arrow is pointing to a file of "peculiar cells" or to normal tracheary elements. In the other figure panels, the arrows all point to peculiar cells which appear irregular in shape and not in a neat cell file. Panel a may not be informative in this regards.

line 391 - not clear here. Higher than what?

Figure 5f - The legend for Figure 5f refers to lines with arrows, but the arrows are not visible in the figure. It is possible that they are just too small to see at the reduced figure size.

line 465 - regulators
